# Sufficient reliability of the behavioral and computational readouts of a probabilistic reversal learning task

**DOI:** 10.3758/s13428-021-01739-7

**Published:** 2022-02-15

**Authors:** Maria Waltmann, Florian Schlagenhauf, Lorenz Deserno

**Affiliations:** 1 Department of Child and Adolescent Psychiatry, Psychosomatics and Psychotherapy, Centre of Mental Health, University of Würzburg, Margarete-Höppel-Platz 1, 97080 Würzburg, Germany; 2 Max Planck Institute for Human Cognitive and Brain Sciences, Stephanstraße 1, 04103 Leipzig, Germany; 3 Department of Psychiatry and Psychotherapy, Charité-Universitätsmedizin Berlin, Campus Charité Mitte, Berlin, Germany; 4 Neuroimaging Center, Technical University of Dresden, Dresden, Germany

**Keywords:** Probabilistic reversal learning, Reliability, Reinforcement learning, Computational modeling, Hierarchical modeling

## Abstract

Task-based measures that capture neurocognitive processes can help bridge the gap between brain and behavior. To transfer tasks to clinical application, reliability is a crucial benchmark because it imposes an upper bound to potential correlations with other variables (e.g., symptom or brain data). However, the reliability of many task readouts is low. In this study, we scrutinized the retest reliability of a probabilistic reversal learning task (PRLT) that is frequently used to characterize cognitive flexibility in psychiatric populations. We analyzed data from *N* = 40 healthy subjects, who completed the PRLT twice. We focused on how individual metrics are derived, i.e., whether data were partially pooled across participants and whether priors were used to inform estimates. We compared the reliability of the resulting indices across sessions, as well as the internal consistency of a selection of indices. We found good to excellent reliability for behavioral indices as derived from mixed-effects models that included data from both sessions. The internal consistency was good to excellent. For indices derived from computational modeling, we found excellent reliability when using hierarchical estimation with empirical priors and including data from both sessions. Our results indicate that the PRLT is well equipped to measure individual differences in cognitive flexibility in reinforcement learning. However, this depends heavily on hierarchical modeling of the longitudinal data (whether sessions are modeled separately or jointly), on estimation methods, and on the combination of parameters included in computational models. We discuss implications for the applicability of PRLT indices in psychiatric research and as diagnostic tools.

**Supplementary Information:**

The online version contains supplementary material available at 10.3758/s13428-021-01739-7.

## Introduction

The Research Domain Criteria (RDoC) framework was introduced as a stepping stone towards a transdiagnostic understanding of mental health and disease (Cuthbert, 2015; Cuthbert & Insel, 2013; Insel, 2014): the expectation was that probing core neurocognitive functions across diagnoses as well as multiple levels of analysis would provide a handle on etiology, pathophysiology, and eventually treatment of specific symptoms and symptom clusters (Cuthbert, 2015; Cuthbert & Insel, 2013; Insel, 2014).

A major roadblock towards clinical applicability has been the insufficient reliability of tasks assessing the core neurocognitive functions outlined in the RDoC (e.g., Elliott et al., 2020; Enkavi et al., 2019; Hedge et al., 2018; Rodebaugh et al., 2016). Poor reliability can arise as a consequence of different issues with measurements, which can be illustrated by looking at how it is typically calculated: the intra-class correlation (ICC) is proportional to the ratio of between-subject variance to overall variance (i.e., the sum of between-subject variance, within-subject variance, and error variance). Thus, reliability can be compromised by high within-subject variance, reflecting a true lack of stability; by high error variance, reflecting a true lack of measurement precision; or by low between-subject variance, reflecting a problem with the resolution of the task. Lately, much attention has been allocated to the latter source of poor reliability. As Hedge et al. (2018) note in their landmark paper, most tasks assessing core neurocognitive processes were developed for research in experimental psychology, where interindividual variance is considered a nuisance, and tasks are designed to reduce this variance. This threatens their reliability (Hedge et al., 2018). And indeed, recent investigations have shown that a wide range of tasks commonly used in neurocognitive research in psychiatry have limited reliability (Enkavi et al., 2019; Hedge et al., 2018). This substantially stymies progress towards clinical applicability because the reliability of a metric imposes an upper bound to potential correlations with other data, such as symptoms or neural data (Elliott et al., 2020; Hedge et al., 2018). It has thus become clear that tasks employed in neurocognitive research in psychiatry should be systematically probed for reliability and improved upon as needed.

In this study, we investigated the reliability of a probabilistic reversal learning task (PRLT). It is designed to measure flexible behavioral adaptation and is frequently used in the field in a transdiagnostic manner (e.g., Culbreth et al., 2016; Reiter et al., 2016, 2017; Tezcan et al., 2017). PRLTs tap into the cognitive control and reward learning constructs of the RDoC’s domains. In such tasks, consecutive choices out of two options are rewarded with a certain probability, such that participants can learn by trial and error which is the more lucrative stimulus. After a while, the contingencies are reversed. This setup is meant to mimic dynamic environments, where actions can have changing values. For example, the canteen’s lunch might be fantastic usually and then turn bland when the chef changes. Adapting to changes in action–outcome contingencies is a core function involving both learning about action–outcome contingencies and cognitively controlling responses, e.g., when previously learned action–reward associations are no longer valid.

The main outcome variables of PRLTs are accuracy, i.e., the number of choices of the currently better stimulus, as well as patterns of stay–switch behavior following feedback such as wins and losses, and the continued choice of an unrewarded stimulus after a reversal (perseveration). Reaction times can be analyzed, but this has been done less frequently for PRLTs. Reduced accuracy has been observed in many psychiatric conditions such as schizophrenia (Schlagenhauf et al., 2014), binge eating disorder (BED) (Reiter et al., 2017), and alcohol use disorder (Reiter et al., 2016). More specific patterns of stay–switch behavior have also been identified, e.g., enhanced perseveration in substance use disorder (Ersche et al., 2008; Reiter et al., 2016; Verdejo-Garcia et al., 2015), enhanced choice switching in BED (Reiter et al., 2017) and schizophrenia (Deserno et al., 2020), and reduced switching in obsessive-compulsive disorder (OCD) (Voon et al., 2015).

In addition to behavioral performance metrics, biologically plausible computational models of reinforcement learning (RL) can be harnessed to capture variation in the processes that underlie observed behavior (Huys et al., 2016). A whole range of models have been applied to PRLTs (e.g., Deserno et al., 2020; Nickchen et al., 2017; Reiter et al., 2017; Wiehler & Peters, 2020), the vast majority of which are based on Q-learning models (Sutton & Barto, 2018; Watkins & Dayan, 1992) in conjunction with a softmax decision policy. In brief, the algorithm learns values associated with the choice of a certain stimulus based on prediction errors—the difference between expected and obtained rewards—and the softmax function probabilistically chooses an action based on those values. Free parameters capture interindividual differences in how the task is performed, for example by means of the speed of learning and forgetting, or choice stochasticity (“noisiness”) and reinforcement sensitivity. The basic model can be extended, for example, to incorporate separate parameters for wins and losses or counterfactual learning, where the value of the unchosen option is updated in parallel with the chosen one. Like the behavioral performance metrics, alterations of model-derived parameters have been associated with psychopathology. Thus, for example, enhanced learning rates for losses have been observed in anorexia nervosa (Bernardoni et al., 2018) and higher choice stochasticity in BED (Reiter et al., 2017), attention-deficit/hyperactivity disorder (ADHD) (Hauser et al., 2014), and schizophrenia (Katthagen et al., 2020). However, RL models can be used not only to estimate parameters from the behavioral data, but also to simulate behavior under certain circumstances. This is a powerful tool for a priori task design and model implementation (Wilson & Collins, 2019). Further, RL models can be applied in the analysis of neural data, yielding trial-by-trial regressors that reflect differences in learning and choice parameters. For example, schizophrenia patients were shown to have reduced ventral striatal prediction error coding than controls (Katthagen et al., 2020; Schlagenhauf et al., 2014), and BED patients showed less engagement of the anterior insula during exploratory decisions (Reiter et al., 2017), reflecting enhanced choice stochasticity. Model-based analysis of neural data can thus help us understand how—in addition to where—the brain performs the PRLT, and how different brains differ in this respect (Gläscher & O’Doherty, 2010).

Based on the alarming reports of insufficient reliability but the great potential of RL modeling for transdiagnostic psychiatry, we probed the internal consistency and retest reliability of behavioral and computational RL metrics of a PRLT. We placed special emphasis on how individual metrics are derived, particularly on whether data were partially pooled across participants and whether priors were used to inform estimates. Thus, following Brown et al. (2020), we compare several analytical approaches in terms of the reliability of the metrics they yield. This is paramount because studies differ substantially in terms of such approaches. In addition, because previous work suggests that retest reliability estimates differ depending on whether the data from each session were modeled separately or jointly (Brown et al., 2020; Rouder & Haaf, 2019), we further compare estimates from joint and separate models. We also demonstrate the discrepancies between these approaches in terms of their ability to recover known reliabilities using simulations.

## Methods

### Participants and procedure

**Fig. 1:**
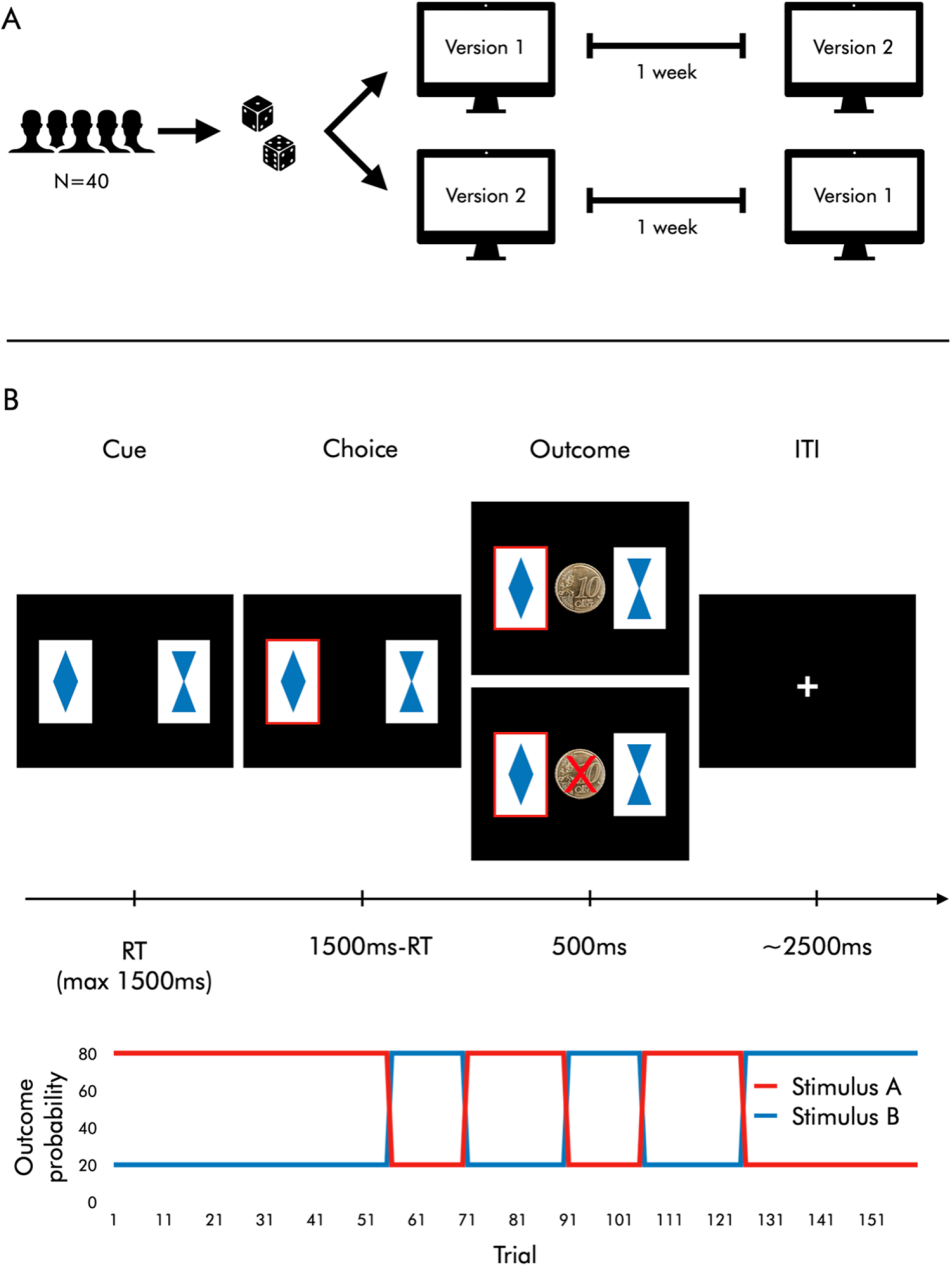
Study and task design. **a** Study design. Forty participants performed two versions of a probabilistic reversal learning task with sessions separated by one week. The order of versions was randomized. **b** *Upper panel*: Trial sequence. In the task, participants make 160 binary choices between abstract stimuli (cards) with different reward probabilities. They are instructed to accumulate as much money as possible. At each trial, the stimuli are shown for a maximum of 1500 ms or until the participant responds. A red frame then appears around the chosen card. This screen is shown for the remainder of 1500 ms, i.e., for 1500 ms minus the response time. Then, a feedback screen with either a picture of a 10-cent coin (win) or a crossed-out picture of a 10-cent coin (loss) is shown for 500 ms. Finally, participants see a fixation cross for a variable inter-trial interval (mean 2500 ms). *Lower panel*: Reward contingencies. In the first 55 trials, the same stimuli each have a 20% and 80% win probability, respectively. Their reward contingencies then reverse five times over the course of the task in a perfectly anti-correlated manner, which requires participants to flexibly adapt their behavior in order to gain money.

Forty healthy participants (20 male) aged 19 to 38 years (*M* = 26.45, *SD* = 3.88) completed a PRLT (Boehme et al., 2015; Deserno et al., 2020; Reiter et al., 2016, 2017) twice, with a one-week gap between sessions. Two different versions were counterbalanced across sessions, such that each participant played different versions on the two test days (Fig. 1a).

In each trial of the task (160 in total), participants had 1.5 seconds to choose between two cards that were associated with different probabilities of winning or losing 10 cents (80/20% and 20/80%, respectively). After making their choice, they were shown a feedback screen (a picture of a 10-cent coin for wins, a picture of a crossed-out 10-cent coin for losses) for 0.5 seconds. Feedback was drawn at each trial with replacement, i.e., if the card with 80% win probability was chosen, a random number between 0 and 1 was drawn from a uniform distribution—if it fell between 0 and 0.8, the participant received a win feedback; if it fell between 0.8 and 1, the participant received a loss feedback. This procedure meant that the actual ratio of wins and losses associated with the stimuli varied across individuals and sessions (from 73.13% to 86.25%). The feedback screen was followed by a variable inter-trial interval with a mean of 2.5 seconds, in which participants were shown a fixation cross. After an initial acquisition phase (1st to 55th trials) the cards’ reward contingencies flipped five times (after the 55th, 70th, 90th, 105th, and 125th trial), such that the previously more lucrative stimulus now became the more frequently losing one, and vice versa. For details, see Fig. 1b.

### Analysis

In our analysis, we systematically investigated the respective retest reliability of several approaches to processing the task data. In addition, we calculated within-session (split-half) reliabilities of the raw behavioral measures.

#### Behavioral metrics

Accuracy was calculated as the probability of choosing the more lucrative stimulus, regardless of actual feedback, estimated by mixed-effects logistic regression and as the proportion of correct trials per person and session. Similarly, stay–switch behavior was calculated as the probability of switching, overall and after wins and losses, respectively, and as the respective proportions per person and session. Perseveration was calculated as the probability of choosing the same incorrect stimulus after two consecutive losses, estimated by mixed-effects logistic regression and as the respective proportions per person and session. Reaction times, both overall and after wins and losses, respectively, were calculated as predicted values estimated by mixed-effects linear regression, and 𝛥RT accordingly, i.e., as the difference in predicted values between win RTs and loss RTs. In addition, we calculated simple averages for overall RTs, win and loss RTs, and 𝛥RT (win RT − loss RT). In the case of metrics derived from mixed-effects models, we compared estimates from separate models for each session with estimates derived from a single model which accounts for the data from both sessions jointly and includes session as a grouping variable nested within subject (Brown et al., 2020). Concretely, this means that the joint regression models took the general form Dependent variable ~ Intercept[*factor] + (Intercept[*factor] |Subject/Session). Because we employ maximal random-effects structures in all our models (Barr et al., 2013), covariances among and between random and fixed effects are taken into account.

We computed mixed-effects logistic and linear regressions in R (version 3.6.1), using the lme4 package (version 1.1-21). Results were considered significant at *p* ≤ .05.

#### Computational models

In order to identify individual differences in processes thought to underlie behavior on this task, we fitted different RL models from two families of models. The first model family included models based on Q-learning (Watkins & Dayan, 1992):

$${Q}_{a,t+1}={Q}_{a,t}+\upalpha \left(r-{Q}_{a,t}\right)$$

Here, *Q*_*a*, *t*_ refers to the expected value of an action *a* at trial t. It is updated at each trial based on the prediction error, i.e., the difference between the feedback just obtained after performing this action, *r*, and the previous expected value *Q*_*a*, *t*_, to form the new expected value *Q*_*a*, *t* + 1_. The learning rate 𝛼 determines how much recent feedback is weighted over the integrated feedback from previous trials (i.e., a learning rate of 1 would only take the last trial into account). The unchosen option is not updated (single update):

$${Q}_{a_{unchosen},t+1}={Q}_{a_{unchosen},t}$$

Learning might be differentially sensitive to wins and losses, resulting in different degrees of value updating after wins versus losses. This can be captured in models with different learning rates for wins and losses.

$${Q}_{a,t+1}={Q}_{a,t}+{\upalpha}_{{win}/{loss}}\left(r-{Q}_{a,t}\right)$$

Because the task has an anti-correlated structure, such that if the chosen stimulus yields a win, the other would have invariably yielded a loss, individuals can use this information to simultaneously update the expected values of both the chosen and the unchosen option. This can be captured in a double update (DU) model:

$${{Q}}_{{{a}}_{{unchosen}},{t}+1}={{Q}}_{{{a}}_{{unchosen}},{t}}+\upalpha \left(\left(-{r}\right)-{{Q}}_{{{a}}_{{unchosen}},{t}}\right)$$

Like the original single update (SU) model, this can be extended with separate learning rates for wins and losses. Finally, it is conceivable that individuals use their knowledge of the task structure and perform double updating but do not update the unchosen option as much as the chosen one. This can be captured using a discount weight 𝜅, which attenuates updating of the unchosen option.

$${{Q}}_{{{a}}_{{unchosen}},{t}+1}={{Q}}_{{{a}}_{{unchosen}},{t}}+\upkappa \upalpha \left(\left(-{r}\right)-{{Q}}_{{{a}}_{{unchosen}},{t}}\right)$$

𝜅 can be added to all DU models, changing only the equations for the unchosen option. In each model, we use a softmax response model to transform values to choice probabilities for each option:

$${p}\left({a}_i\right)=\frac{{\exp}\left(\upbeta {Q}_{a_i}\right)}{\sum_{j=1}^K{\exp}\left(\upbeta {Q}_{a_j}\right)}$$

The parameter β, the softmax inverse temperature or choice sensitivity, influences the extent to which a difference in values translates into a difference in choice probability by determining the steepness of the softmax sigmoid. If β is large, choices are more deterministic or exploitative; if it is small, choices are more stochastic or explorative, such that differences in values exert less influence on action selection. Like learning, choice stochasticity may be differentially sensitive to wins and losses, resulting in asymmetric staying and switching (e.g., with higher win β, a person’s tendency to stay after a win would be stronger than their tendency to switch after loss). This can be captured in separate softmax temperature parameters for trials after receiving wins or losses:

$${p}\left({a}_i\right)=\frac{{\exp}\left({\upbeta}_{{win}/{loss}}{Q}_{a_i}\right)}{\sum_{j=1}^K{\exp}\left({\upbeta}_{{win}/{loss}}{Q}_{a_j}\right)}$$

The different combinations of parameters—SU or DU, single or separate learning rates and temperature parameters for wins and losses—yield a total of 12 models.

In the second model family, we use a reinforcement sensitivity parameter ρ instead of a softmax temperature parameter (dropped in this family) to quantify choice stochasticity:

$${Q}_{a,t+1}={Q}_{a,t}+\upalpha \left(\uprho r-{Q}_{a,t}\right)$$

Unlike the inverse temperature parameter, which influences choice stochasticity by determining the steepness of the softmax, the reinforcement sensitivity ρ does this by determining the maximum difference between expected values, thus posing a lower bound to choice stochasticity. The effect on choice probabilities is essentially the same, but the models can have different estimation properties and may differ in their interpretation under certain circumstances (Huys et al., 2013; Katahira, 2015). As with the softmax temperature models, we fit 12 models in the reinforcement sensitivity family, iteratively including separate learning rates for wins and losses, separate reinforcement sensitivities for wins and losses, double updating, and weighted double updating.

#### Model fitting

We inverse-logit-transformed the learning rates and DU weights (𝛼, 𝜅) in both model families in order to constrain them to their natural range (0 and 1). For models with a single reinforcement sensitivity (𝜌) or softmax temperatures (𝛽), we used an exponential transform to ascertain that they were positive; for models with separate reinforcement sensitivities for wins and losses, the parameters were left in native space. Parameter estimation was performed in MATLAB R2020b using the emfit toolbox (Huys et al., 2011, 2012; Huys & Schad, 2015). We applied and compared three different approaches to parameter estimation (maximum likelihood [ML], maximum a posteriori estimation with uninformative priors [MAP0], and maximum a posteriori estimation with empirical priors [EM-MAP]). In standard ML estimation, the quantity to be maximized is *log*(*p*(*data| θ*)). In MAP estimation, a regularizing prior on 𝜃 is provided, such that the quantity to be maximized becomes *∝ log*(*p*(*data| θ) ∗ p*(*θ*)). For MAP0 estimation, we defined an uninformative Gaussian prior with a mean of 0 and variance of 10 (default in the emfit toolbox). For EM-MAP, we used empirical Gaussian priors on our parameters (*p*(*θ*| *μ*, *σ*)), inferred from the multivariate distribution of the estimates across subjects in an expectation maximization procedure (Huys et al., 2012).

We used all three estimation methods to fit the sessions separately, i.e., maximizing the (posterior) likelihood of the data from each session one at a time, as well as jointly, i.e., maximizing the overall (posterior) likelihood of the data pooled across the two sessions. For the joint estimation, we concatenated the data from both sessions but fitted separate parameters for each session. Concretely, this meant that we fit one set of parameters for the first 160 trials (from session one) and another set for the second 160 trials (from session two), resetting Q-values for the first trial of session two. Thus, both approaches yield separate parameters for the first and second session. However, when estimated jointly, covariances between parameters across sessions are taken into account in a multivariate prior in the case of EM. Note that this does not mean, for example, that the learning rate for sessions one and two shared a prior. Instead, each parameter is accounted for by its own mean and variance in addition to the covariances between parameters.

To minimize the risk of local minima, we restarted the optimization 10 times (for EM at each M step) at different random starting points, taking the best iteration forward in the case of EM. In addition, we repeated the estimation procedure 10 times, and used the final results with the maximum (posterior) likelihood for reliability analysis. We performed model selection on the estimated models based on the integrated Bayesian information criterion (Huys et al., 2012).

MATLAB’s *fminunc* function, which the emfit toolbox we employed utilizes, allows users to choose between a quasi-Newtonian and a trust-region algorithm for optimization. The latter requires the user to supply analytically calculated gradients to guide the search of the optimizer but can help improve the optimizer’s performance and the robustness of the results (Daw, 2011). All results reported in the main text are based on trust-region estimates. However, as a supplementary analysis, we also performed model fitting without supplying gradients, i.e., using a quasi-Newtonian algorithm for optimization, for comparison. Detailed results are reported in Supplementary Fig. 1 and Supplementary Table 2.

#### Parameter recoverability

In order to ensure good fit on a qualitative level, we extracted the parameters of the best-fitting models from each family and generated 100 simulated datasets for 38 participants based on the respective algorithms. We then plotted the behavioral metrics derived from the generated data (averaged across simulations) against those derived from the original data for visual comparison. Further, we probed the recoverability of the parameter estimates by refitting the models to the generated data and computing the average correlation between the resulting estimates and the underlying true values.

In order to show recoverability across the model space, we further extracted the parameters of eight models of varying complexity (four from each model family), and simulated 10 datasets for 38 participants for each model based on the respective algorithms. We then refitted the same eight models to each dataset to probe model and parameter recoverability. In the interest of space, the results of this analysis are reported in the supplement.

#### Reliability assessment

In order to assess the retest reliability of the behavioral metrics and model-derived parameters, we computed intra-class correlations (ICCs), more specifically ICCs(A,1) in McGraw and Wong’s (1996) notation, between the metrics across time points (McGraw & Wong, 1996; Qin et al., 2019). As Qin et al. (2019) note, this type of ICC (i.e., a two-way mixed, single-measure, absolute-agreement ICC) is appropriate for estimations of retest reliability in which time is a design factor, the space between time points is identical across subjects, there is only one observation per subject and time point, and parameter values are assumed to be constant across time points. Calculations were performed using the irr package (version 0.84.1) in R (version 4.1.0) for the raw behavioral metrics and the ICC toolbox (version 1.3.1.0) in MATLAB 2019b for the indices derived from computational modeling. This was done for convenience; however, the packages employ the same formulae and produce equivalent results.

We calculated ICCs(A,1) for means, predicted values, and fitted parameters (i.e. point estimates); however, certain ICCs can also be obtained directly from variances estimated as part of the model (e.g., Brown et al., 2020). The advantage of this approach is that variances that are estimated as part of a model contain information as to the precision of the predicted values or fitted parameters. Specifically, model-calculated variances reflect the sum of the variance of point estimates and the mean standard error around them. This latter term is absent from the variances calculated on predicted values. To our knowledge, the variance components estimated as part of logistic and linear regressions using lme4 and as part of the computational model fitting using emfit do not permit the calculation of ICCs(A,1), but the former allow the calculation of ICCs(1) and the latter Pearson correlations. We therefore report model-calculated ICCs(1) for the behavioral metrics and model-calculated Pearson correlations for the EM-MAP-estimated parameters, alongside the respective metrics based on point estimates for comparability. We computed model-derived ICCs(1) in McGraw and Wong’s (1996) notation for the behavioral metrics on the basis of the variance components accessed using the *get_variance* function as part of the *insight* package for lme4 model fits. Specifically, we took the ratio of the variance explained by the random effect of subject (between-subject variance) and the sum of that variance and the variance explained by session within subject (within-subject variance). Model-derived Pearson correlations between parameters were calculated by dividing the covariance of the equivalent parameters from each session by the product of the square roots of the variances of the individual parameters.

We interpreted the ICC coefficients according to Cicchetti’s (1994) guidelines (“[W]hen the reliability coefficient is below .40, the level of clinical significance is poor; when it is between .40 and .59, the level of clinical significance is fair; when it is between .60 and .74, the level of clinical significance is good; and when it is between .75 and 1.00, the level of clinical significance is excellent.”) (Cicchetti, 1994).

In order to assess internal consistency of the behavioral metrics, we re-estimated them in separate logistic and linear regressions for the odd and even trials of each session and calculated their correlation. We report the correlations with and without Spearman–Brown correction (${r}_{SB}=\frac{2r}{1+r}$), which accounts for deflated correlations due to the reduced number of observations.

#### Simulations: recoverability of reliability

In order to probe different approaches of index estimation in terms of their ability to recover true reliabilities, we simulated correlated binary (choice-like) and continuous (reaction time-like) data (500 datasets with 160 trials for 38 subjects on two sessions each). Specifically, for each dataset, we simulated normally distributed indices for 38 subjects that were correlated at *r* = .3, *r* = .5, *r* = .7, and *r* = .9 across sessions. We then took those indices forward and simulated trial-by-trial data. For binary data, we drew a random number between 0 and 1 at each trial and compared it to the simulated index; if it was smaller, the trial was coded 1, and if it was larger, the trial was coded 0. For continuous data, we sampled, at each trial, from a normal distribution around the index. For each dataset, we then computed ICCs based on means, predicted values from logistic/linear regressions for each session separately, predicted values from logistic/linear regressions for both sessions simultaneously, and based on variance components as extracted from the joint model, as we did for the original data. We then compared the resulting ICCs to the true correlations.

#### Data and code availability

The data and the scripts underlying the analyses in this article are available on the Open Science Framework (osf.io/4ng3e).

## Results

As confirmed by a binomial test, two participants performed at chance level (percent correct responses ~ 50%) in one or both sessions. We excluded them from all further analyses for two reasons: first, because their task data may be suggestive of noncompliance; second, and most importantly, because they deviate > 3 standard deviations from the other participants in terms of accuracy. Thus, including their data substantially increases between-subject variance and might thereby inflate reliability estimates in a non-meaningful manner.

### Raw behavioral analysis

#### Retest reliability

##### Accuracy

**Fig. 2:**
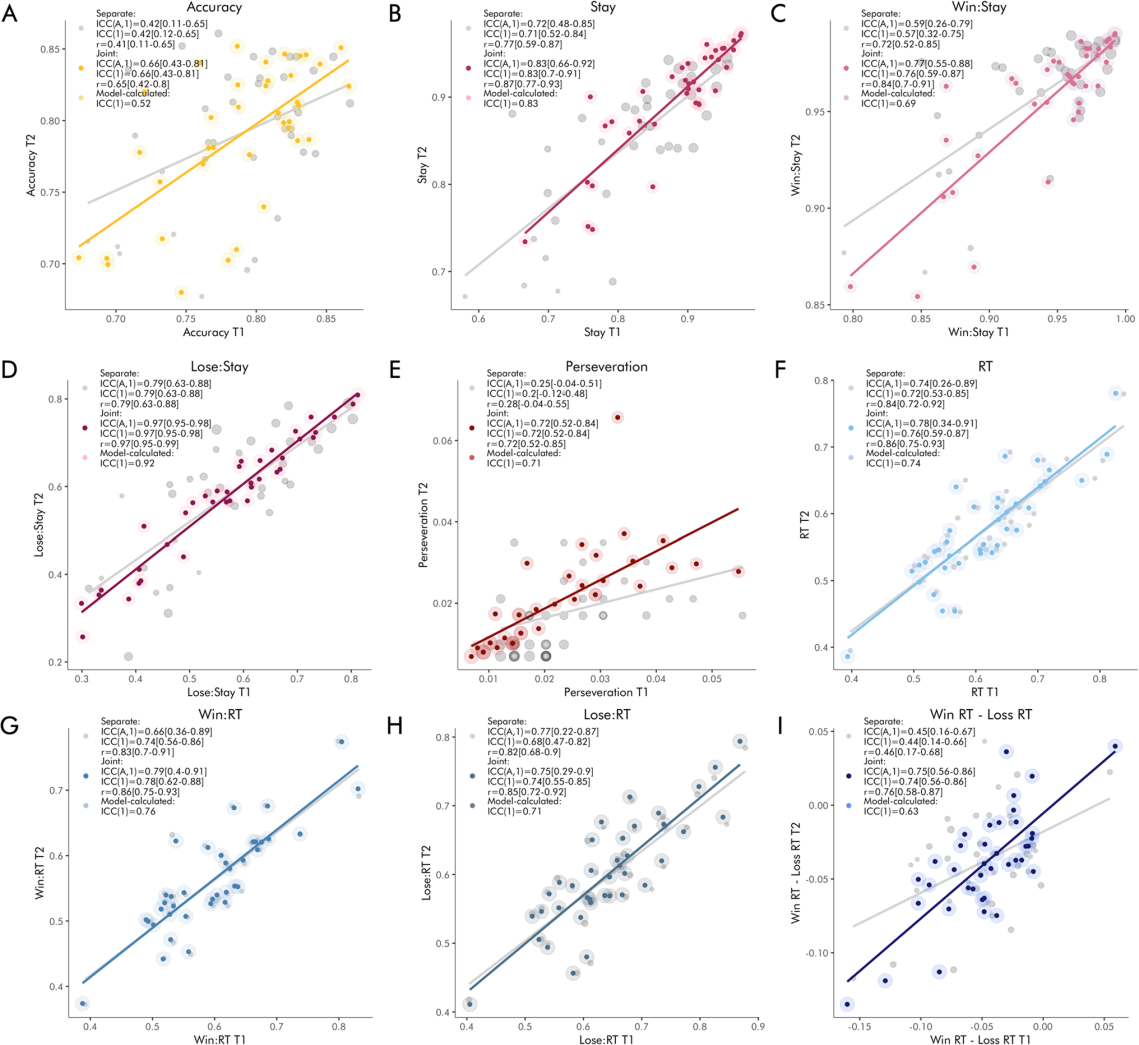
Retest reliability of raw behavioral performance indices by joint versus separate modeling of the two sessions. The scatterplots reflect the association between mixed-model-derived performance indices for the two sessions. Gray dots and lines represent estimates based on separate modeling of sessions; colored dots represent estimates based on joint modeling of sessions. The size of the shaded area around the point estimates is proportional to their standard error. We report ICCs (A,1), ICCs (1), and Pearson correlations in the legend of each panel, with confidence intervals in square brackets, as well as model-derived ICCs(1) which take standard errors into account, for **a** accuracy, **b** stay behavior overall, **c** stay behavior after wins, **d** stay behavior after losses, **e** perseveration, **f** reaction times overall, **g** reaction times after wins, **h** reaction times after losses, **i** difference in reaction times after wins and losses.

Accuracy had fair reliability when estimated based on the proportion of correct choices (ICC(A,1) = .41, ICC(1) = .42, *r* = .41) and when sessions were modeled separately (ICC(A,1) = .42, ICC(1) = .42, *r* = .41), and good reliability when they were modeled jointly (ICC(A,1) = .66, ICC(1) = .66, *r* = .65) (Fig. 2a). The model-calculated ICC was somewhat lower, at ICC(1) = .53.

##### Stay–switch behavior

Mixed-effects logistic regression revealed a main effect of previous feedback on switching (*β* = 1.486, z = 24.25, *p* < .0001), such that participants switched more after losses than wins. The reliabilities of switching overall and after wins and losses respectively differed depending on estimation methods: they were good to excellent when estimated based on the mean proportion of switches (ICC(A,1)_overall_ = .72, ICC(1)_overall_ = .72, r_overall_ = .73; ICC(A,1)_win_ = .60, ICC(1)_win_ = .58, r_win_ = .67; ICC(A,1)_loss_ = .77, ICC(1)_loss_ = .77, r_loss_ = 0.77); fair to excellent when estimated based on separate models for each session (ICC(A,1)_overall_ = .72, ICC(1)_overall_ = .71, r_overall_ = 0.77; ICC(A,1)_win_ = .59, ICC(1)_win_ = .57, r_win_ = 0.72; ICC(A,1)_loss_ = .79, ICC(1)_loss_ = .79, r_loss_ = 0.79); and good to excellent when based on the joint model (ICC(A,1)_overall_ = .83, ICC(1)_overall_ = .83, r_overall_ = 0.87; ICC(A,1)_win_ = .77, ICC(1)_win_ = .67, r_win_ = 0.84; ICC(A,1)_loss_ = .97, ICC(1)_loss_ = .97, r_loss_ = 0.97) (Fig. 2c–e). The model-calculated ICCs were close to the ICCs based on the predicted values of the joint model (ICC(1)_overall_ = .83, ICC(1)_win_ = .69, ICC(1)_loss_ = .92)

##### Perseveration

Perseveration had poor reliability when based on the proportion of perseverative errors (ICC(A,1) = .35, ICC(1) = .34, *r* = .36), poor reliability when sessions were modeled separately (ICC(A,1) = .25, ICC(1) = .2, *r* = .28), and good reliability when they were modeled jointly (ICC(A,1) = .72, ICC(1) = .72, *r* = .72) (Fig. 2, Panel B). The model-calculated ICC was nearly identical to the ICC based on the predicted values of the joint model (ICC(1) = .71).

##### Reaction times

Mixed-effects linear regression revealed significant main effects of previous feedback on reaction times (*β* = −0.022, *t*(37.04) = −6.211, *p* < .0001), such that people responded faster after positive feedback. The reliability of reaction times overall, after wins and losses respectively, and of the difference between them, differed depending on estimation methods: the reliabilities ranged from fair to excellent when estimated based on average RTs (ICC(A,1)_overall_ = .74, ICC(1)_overall_ = .73, r_overall_ = 0.84; ICC(A,1)_win_ = .75, ICC(1)_win_ = .74, r_win_ = 0.82; ICC(A,1)_loss_ = .70, ICC(1)_loss_ = .68, r_loss_ = 0.80; ICC(A,1)_delta_ = .47, ICC(1)_delta_ = .47, r_delta_ = 0.48); from fair to excellent when estimated based on separate models (ICC(A,1)_overall_ = .74, ICC(1)_overall_ = .72, r_overall_ = 0.84; ICC(A,1)_win_ = .66, ICC(1)_win_ = .74, r_win_ = 0.83; ICC(A,1)_loss_ = .77, ICC(1)_loss_ = .68, r_loss_ = 0.82; ICC(A,1)_delta_ = .45, ICC(1)_delta_ = .44, r_delta_ = 0.46); and from good to excellent when estimated based on the joint model (ICC(A,1)_overall_ = .78, ICC(1)_overall_ = .76, r_overall_ = 0.86; ICC(A,1)_win_ = .79, ICC(1)_win_ = .78, r_win_ = 0.86; ICC(A,1)_loss_ = .75, ICC(1)_loss_ = .74, r_loss_ = 0.85; ICC(A,1)_delta_ = .75, ICC(1)_delta_ = .74, r_delta_ = 0.76) (Fig. 2f–i). The model-calculated ICCs lay between the ICCs based on the predicted values of the separate models and the joint model (ICC(1)_overall_ = .74, ICC(1)_win_ = .76, ICC(1)_loss_ = .71, ICC(1)_delta_ = .63).

##### Variance components

**Fig. 3:**
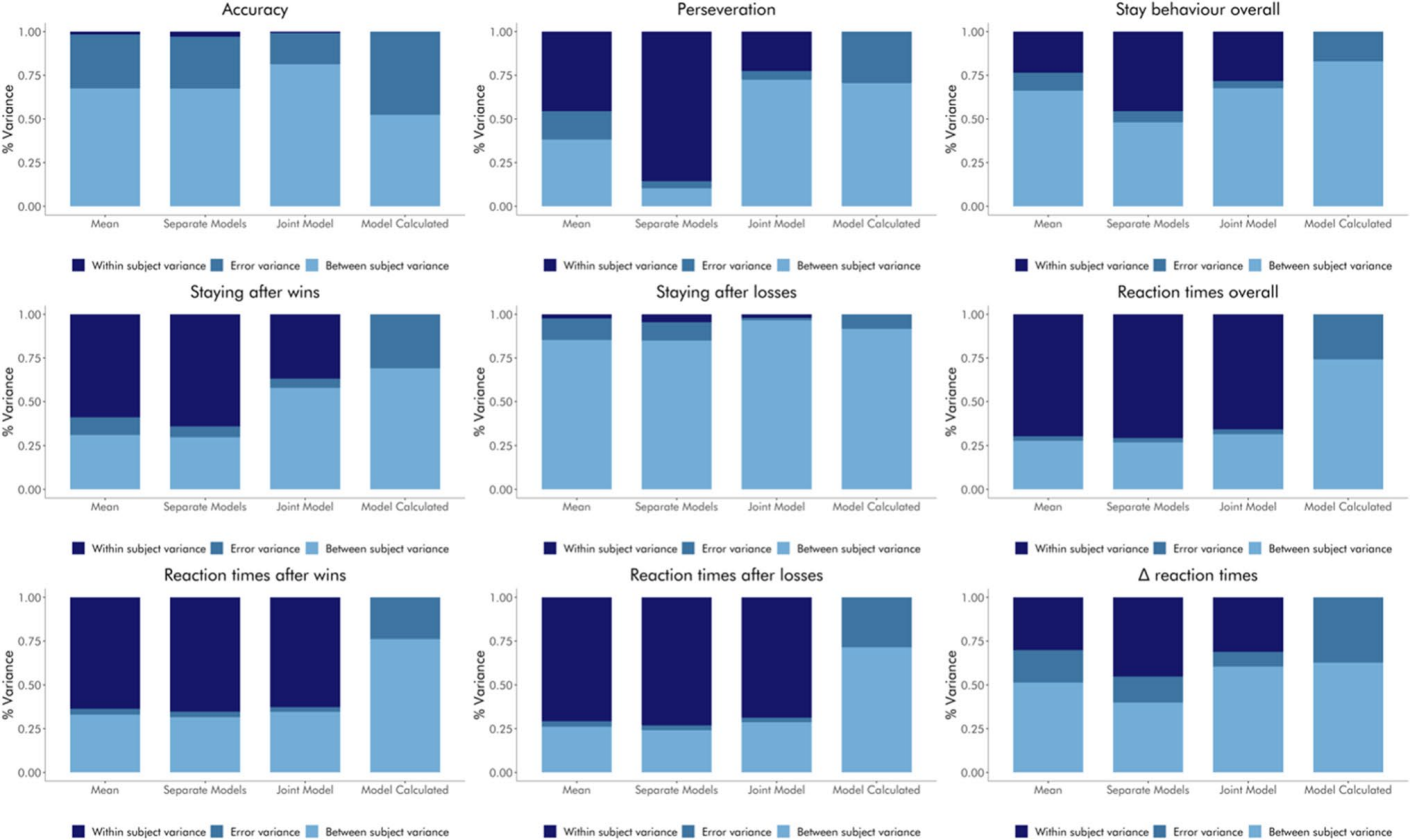
Relative variance components of the raw behavioral performance indices by estimation methods. In each plot, the left bar represents means, the second to left bar represents estimates based on separate modeling of sessions, the second to right bar represents estimates based on joint modeling of sessions, and the right bar represents model-calculated variance components. Midnight blue sections of the bars reflect within-subject variance (session effects), steel blue sections of the bars reflect error variance, and sky blue sections of the bars reflect between-subject variance. Note that because of the way the model is set up, it is not possible to extract session variance from the model output; this component is therefore missing in the rightmost bars.

Partitioning the variance of the metrics into within-subject variance (systematic effects of session), between-subject variance, and error variance, we see that the relative amount of error variance decreases depending on the estimation method (Fig. 3). Thus, the error variance is largest for means, somewhat smaller for estimates based on separate models for each session, and substantially smaller for estimates based on the joint models.

#### Recoverability of retest reliability

**Fig. 4:**
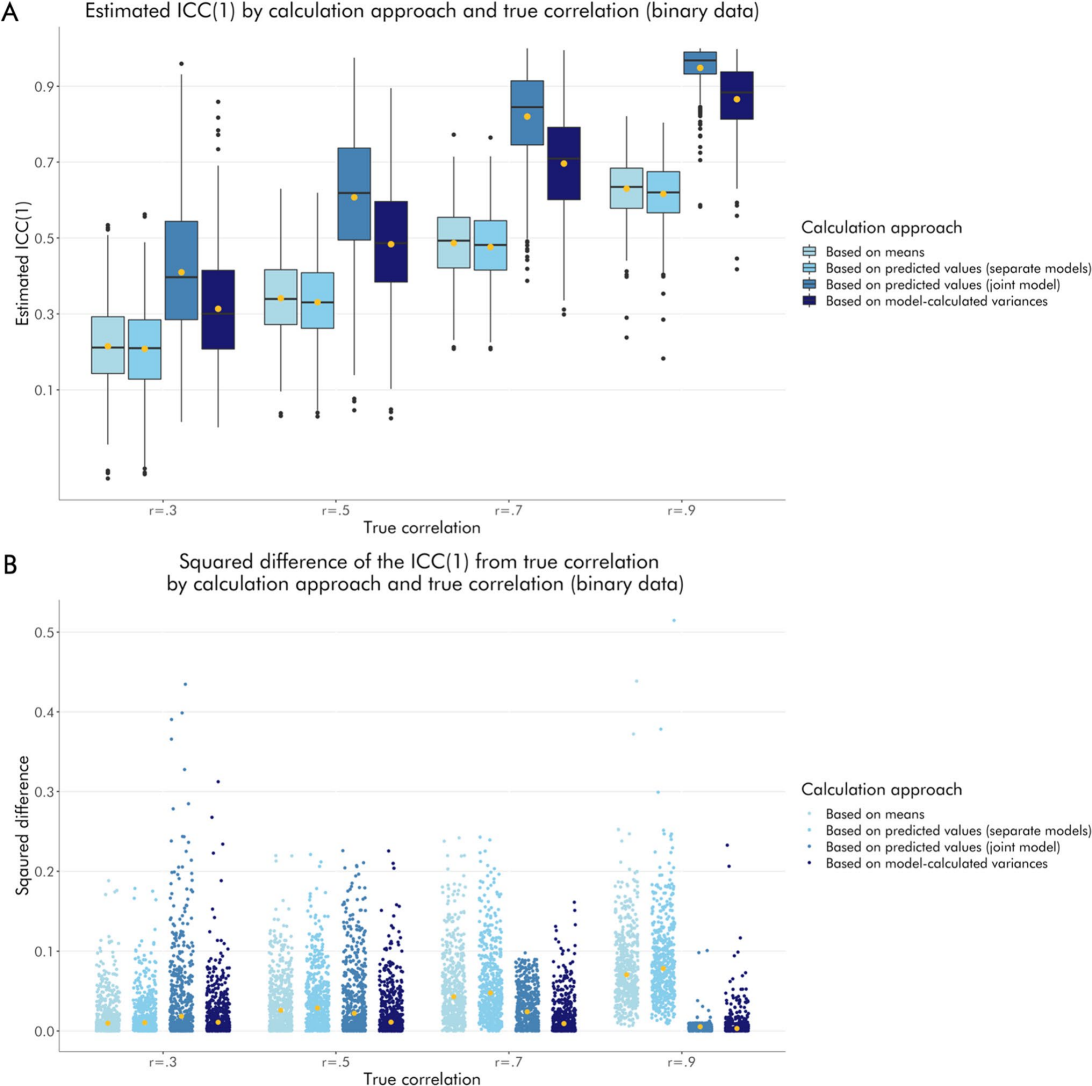
Recoverability of retest reliability in simulated binary data by estimation method. **a** Boxplots show the distribution of estimated ICCs across 500 simulations. Light blue boxes reflect ICCs based on means, sky blue boxes reflect ICCs based on estimates based on separate modeling of sessions, steel blue boxes reflect ICCs based on estimates based on joint modeling of sessions, and midnight blue boxes reflect ICCs based on model-calculated variances. Yellow dots represent mean values. **b** scatterplots showing the squared difference between the true correlation and the estimated ICC, per dataset and estimation method. Light blue dots reflect ICCs based on means, sky blue dots reflect ICCs based on estimates based on separate modeling of sessions, steel blue dots reflect ICCs based on estimates based on joint modeling of sessions, and midnight blue dots reflect ICCs based on model-calculated variances. Yellow dots represent mean values.

Using simulations (500 simulated datasets, each containing 160 trials for each of two sessions for 38 subjects), we investigated differences in recoverability of retest reliability between estimation methods for both binary and continuous data. For binary data, we show that means and estimates based on separate modeling of sessions tend to substantially underestimate the true retest reliability, while the estimates based on joint modeling of sessions tend to moderately overestimate it (Fig. 4a). Model-calculated ICCs (from the joint model) appear to be the most accurate, with no obvious bias towards over- or underestimation. The absolute difference between true and estimated reliability tends to be relatively small for all approaches, although there is a distinctive advantage of reliabilities derived from models that account for both sessions simultaneously when the true values are high (Fig. 4b).

**Fig. 5:**
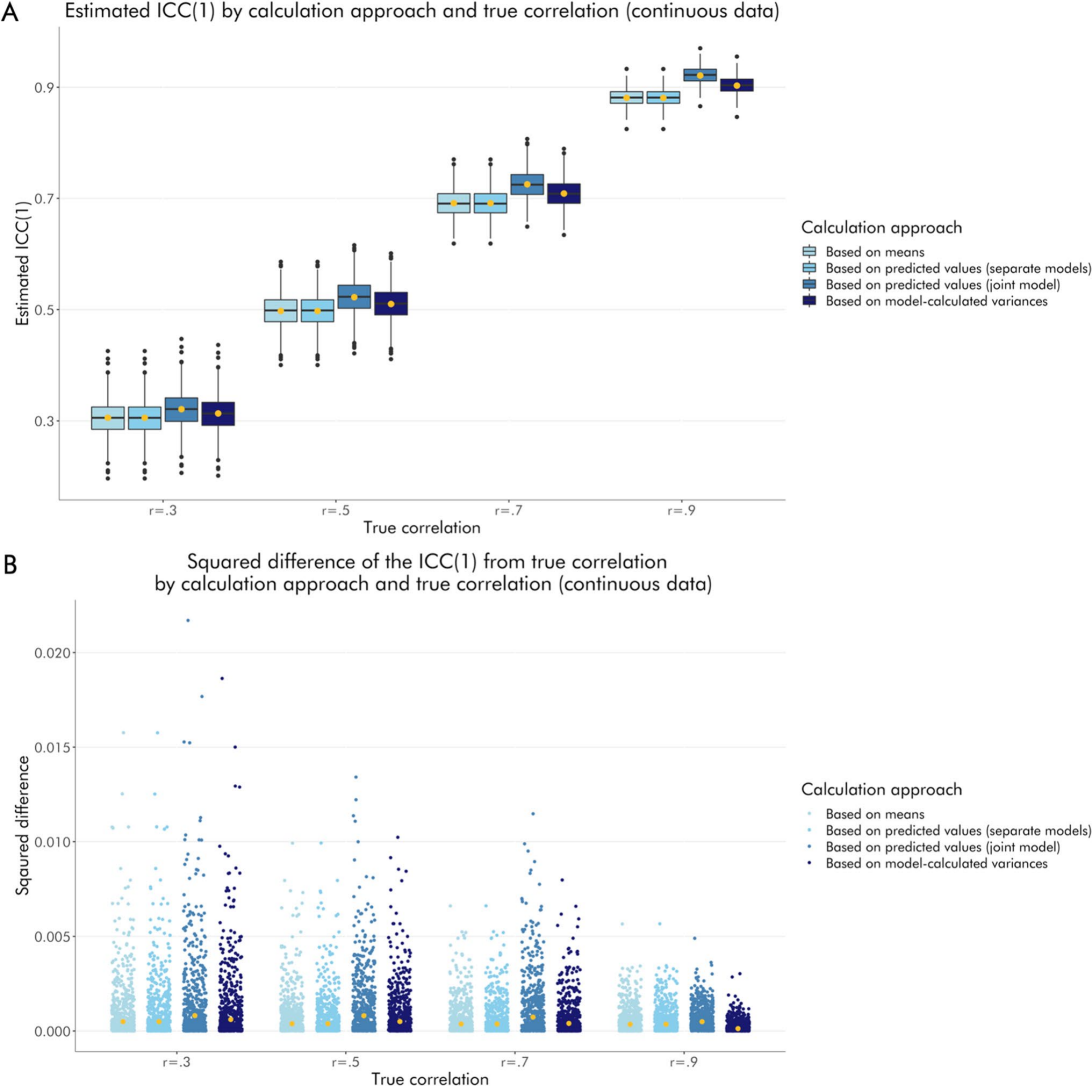
Recoverability of retest reliability in simulated continuous data by estimation method. **a** Boxplots show the distribution of estimated ICCs across 500 simulations. Light blue boxes reflect ICCs based on means, sky blue boxes reflect ICCs based on estimates based on separate modeling of sessions, steel blue boxes reflect ICCs based on estimates based on joint modeling of sessions, and midnight blue boxes reflect ICCs based on model-calculated variances. Yellow dots represent mean values. **b** Scatterplots showing the squared difference between the true correlation and the estimated ICC, per dataset and estimation method. Light blue dots reflect ICCs based on means, sky blue dots reflect ICCs based on estimates based on separate modeling of sessions, steel blue dots reflect ICCs based on estimates based on joint modeling of sessions, and midnight blue dots reflect ICCs based on model-calculated variances. Yellow dots represent mean values.

For continuous data, we show that all estimation approaches yield ICCs that are reasonably close to the true correlations (Fig. 5a). Similar to our observations in the binary data, we see that means and estimates based on separate modeling of sessions tend to slightly underestimate reliability, especially when the true correlation is high. Likewise, estimates based on joint modeling of sessions tend to moderately overestimate true correlations. Model-calculated ICCs again show no obvious bias towards over- or underestimation. Again, similar to our observation in the binary data, the absolute difference between true and estimated reliability tends to be small for all approaches (Fig. 5b).

#### Internal consistency

**Fig. 6:**
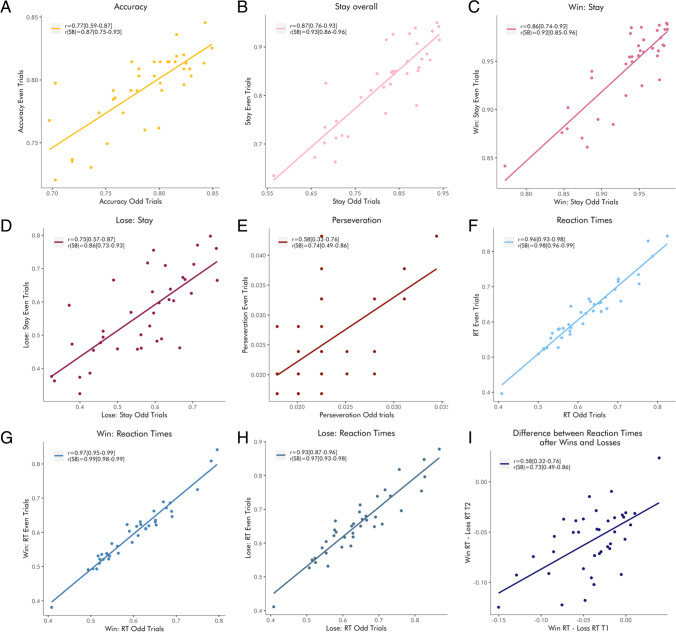
Internal consistency of raw behavioral performance indices. The scatterplots reflect the association between performance indices based on estimates from a mixed model including only even trials and those based on estimates from a mixed model including only odd trials. We report Pearson correlations with and without Spearman–Brown correction in the legend of each panel, with confidence intervals in brackets for **a** accuracy, **b** stay behavior overall, **c** stay behavior after wins, **d** stay behavior after losses, **e** perseveration, **f** reaction times overall, **g** reaction times after wins, **h** reaction times after losses, and **i** difference in reaction times after wins and losses.

The behavioral metrics proved to have good or excellent internal consistency (i.e., split-half reliability) for the first session, with the exception of the perseveration index and the difference in reaction times between wins and losses, whose internal consistency was merely fair before Spearman–Brown correction (Fig. 6).

In the second session, split-half reliabilities were similarly good to excellent, with the exception of the index quantifying the difference in reaction times after wins and losses, whose internal consistency was merely fair before Spearman–Brown correction (accuracy *r* = .84 [.71–.92], *r*_SB_ = 0.91 [0.83–.96]; stay *r* = .76 [.58–.87], *r*_SB_ = 0.86 [0.74–.93]; win stay *r* = .74 [.54–.85], *r*_SB_ = 0.85 [0.7–.92]; lose stay *r* = .70 [.49–.83], *r*_SB_ = 0.82 [0.66–.91]; perseveration *r* = .65 [.41–.79], *r*_SB_ = 0.78 [0.58–.89]; RT *r* = .97 [.93–.98], *r*_SB_ = 0.98 [0.97–.99]; win RT: *r* = .97 [.95–.99], *r*_SB_ = 0.99 [0.97–.99]; loss RT: *r* = .90 [.82–.95], *r*_SB_ = 0.95 [0.90–.97]; delta RT: *r* = .35 [.03–0.60], *r*_SB_ = 0.52 [0.07–.75]).

## Computational modeling

### Model comparison

**Fig. 7:**
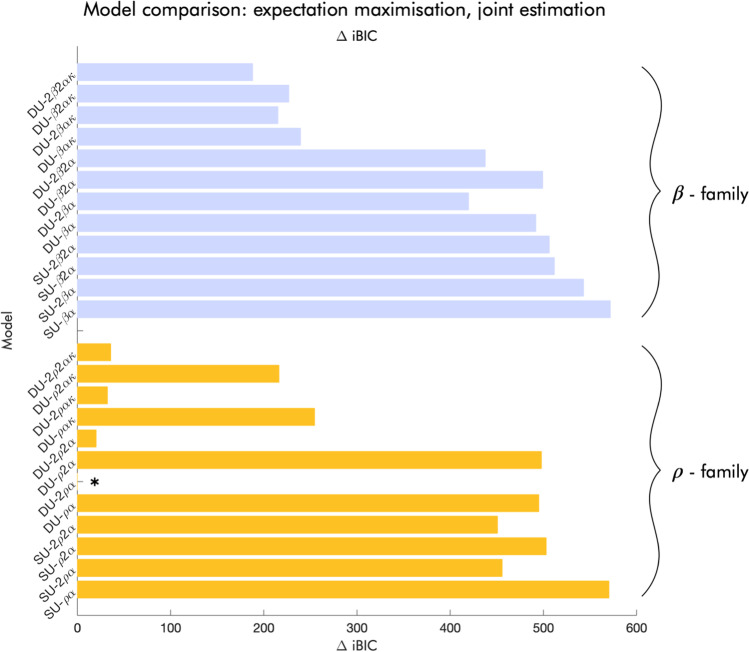
Model comparison based on the integrated Bayesian information criterion (iBIC). Bars represent the distance from the model with the best evidence (lowest iBIC). Blue bars at the top of the plot represent models from the softmax (𝛽) family; yellow bars at the bottom of the plot represent models from the reinforcement sensitivity (𝜌) family. As represented by an asterisk, the overall winning model is a double update model with separate reinforcement sensitivities for wins and losses and a single learning rate (DU-2𝜌𝛼). Within the softmax family, the most complex model, a weighted double update with separate softmax temperatures and learning rates for wins and losses (DU-2𝛽2𝛼𝜅), had the best evidence. *SU* single update, *DU* double update, 𝛽 softmax temperature, 𝛼 learning rate, 𝜅 double update weight, 𝜌 reinforcement sensitivity.

Within the softmax family, model comparison using the integrated BIC (iBIC) showed that a weighted DU model with separate learning rates and softmax temperatures for wins and losses (DU-2𝜌2𝛼𝜅) had the best evidence, as reported previously (Reiter et al., 2016, 2017) (Fig. 7). Within the reinforcement sensitivity family, a full DU model with a single learning rate and separate reinforcement sensitivities for wins and losses (DU-2𝜌𝛼) proved the most parsimonious in accounting for the data, as also reported previously on a slightly different task (Schlagenhauf et al., 2014). This model was also superior across both model families. We report the respective reliabilities of the parameters of the winning model from each family. For the reliabilities of the other models, see Supplementary Tables 1 through 3.

### Parameter reliability

**Fig. 8:**
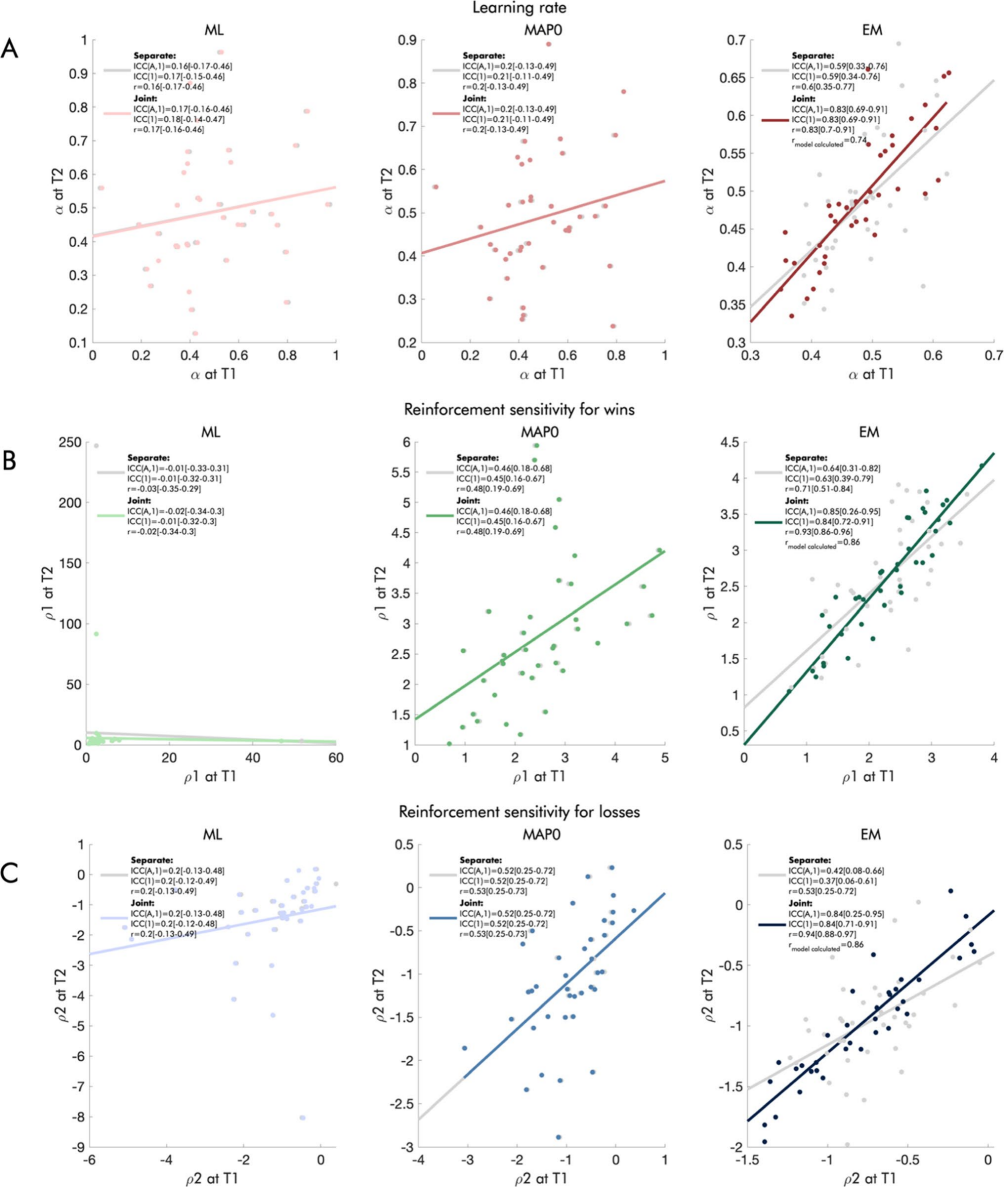
Reliability of the parameters derived from the DU-2𝜌𝛼 model, by estimation method and joint versus separate modeling of the two sessions. The scatterplots show the association between the parameters derived from the DU-2𝜌𝛼 model for each session. In the left column, we show maximum likelihood (ML) estimates; in the middle column, we show maximum a posteriori estimates with weakly informative priors (MAP0); in the right column, we show maximum a posteriori estimates with empirical group-level priors (EM). Gray dots show estimates from separate models for each session; colored dots show estimates from the joint models. We report ICCs(A,1), ICCs (1), and Pearson correlations in the legend of each panel, with confidence intervals in square brackets, as well as model-calculated Pearson correlations where possible, for **a** learning rate, **b** reinforcement sensitivity for wins, and **c** reinforcement sensitivity for losses.

**Fig. 9:**
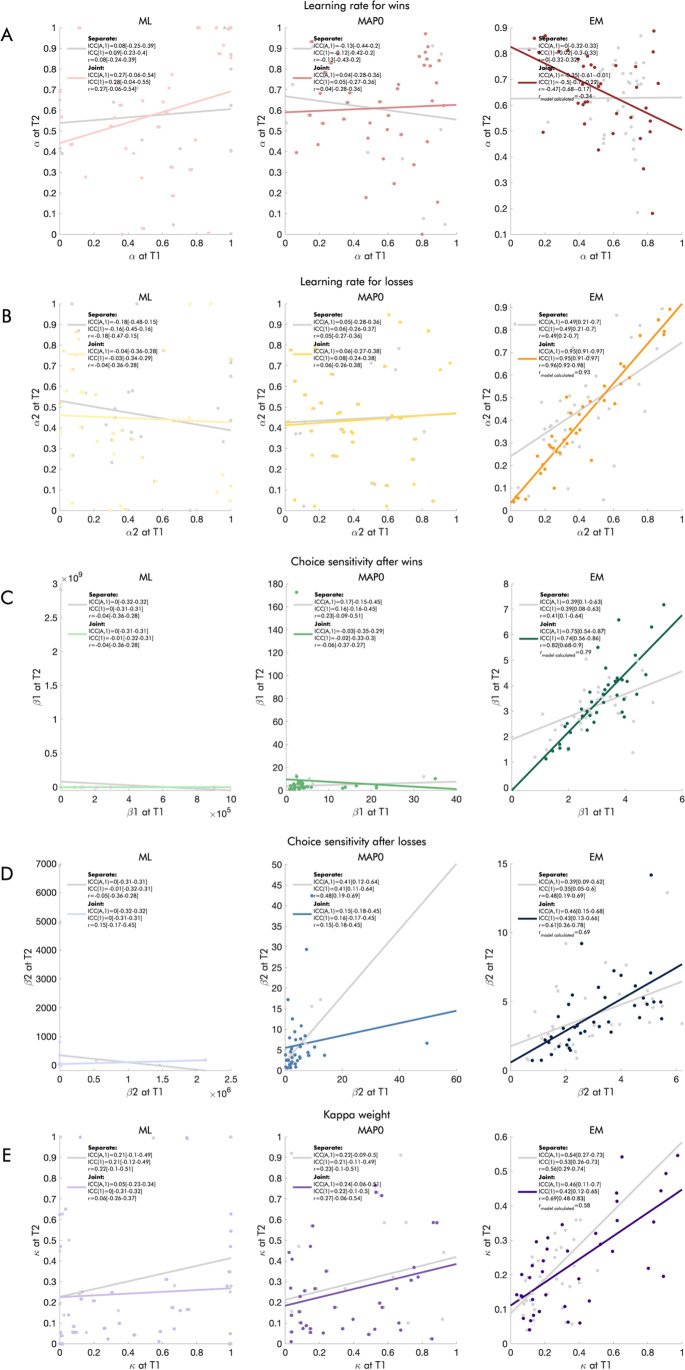
Reliability of the parameters derived from the DU-2𝛽2𝛼𝜅 model, by estimation method and joint versus separate modeling of the two sessions. The scatterplots show the association between the parameters derived from the DU-2𝛽2𝛼𝜅 model for each session. In the left column, we show maximum likelihood (ML) estimates; in the middle column, we show maximum a posteriori estimates with weakly informative priors (MAP0); in the right column, we show maximum a posteriori estimates with empirical group-level priors (EM). Gray dots show estimates from separate models for each session; colored dots show estimates from the joint models. Because covariances between parameters are not taken into account in ML and MAP0 estimation, we expected the parameter values from the joint and separate estimations, and hence their reliabilities, to be the same. This was true for the DU-2𝜌𝛼 model (Fig. 8) but not the DU-2𝛽2𝛼𝜅 model. Here, some variation was apparent, pointing towards lower identifiability due to a higher correlation between parameters. We report ICCs (A,1), ICCs (1), and Pearson correlations in the legend of each panel, with confidence intervals in square brackets, as well as model-calculated Pearson correlations where possible, for **a** learning rate for wins, **b** learning rate for losses, **c** choice sensitivity after wins, **d** choice sensitivity after losses, and **e** double update weight.

In both the DU-2𝜌𝛼 and the DU-2𝛽2𝛼𝜅 models, the estimation method had a considerable effect on reliability. Thus, parameters estimated using ML generally had very poor reliabilities, those estimated using MAP0 had poor or moderate reliabilities, and parameters estimated using EM had fair to excellent reliabilities in the reinforcement sensitivity family (Fig. 8) and poor to excellent reliabilities in the softmax family (Fig. 9). The reliability of the EM-MAP estimates, where covariances between parameters are included in the multivariate prior structure, benefitted considerably from joint estimation of both sessions. Pearson correlations based on model-calculated variances yielded good to excellent reliability for all parameters in the reinforcement sensitivity family (Fig. 8), and poor to excellent reliabilities in the softmax family (Fig. 9).

### Variance components

**Fig. 10:**
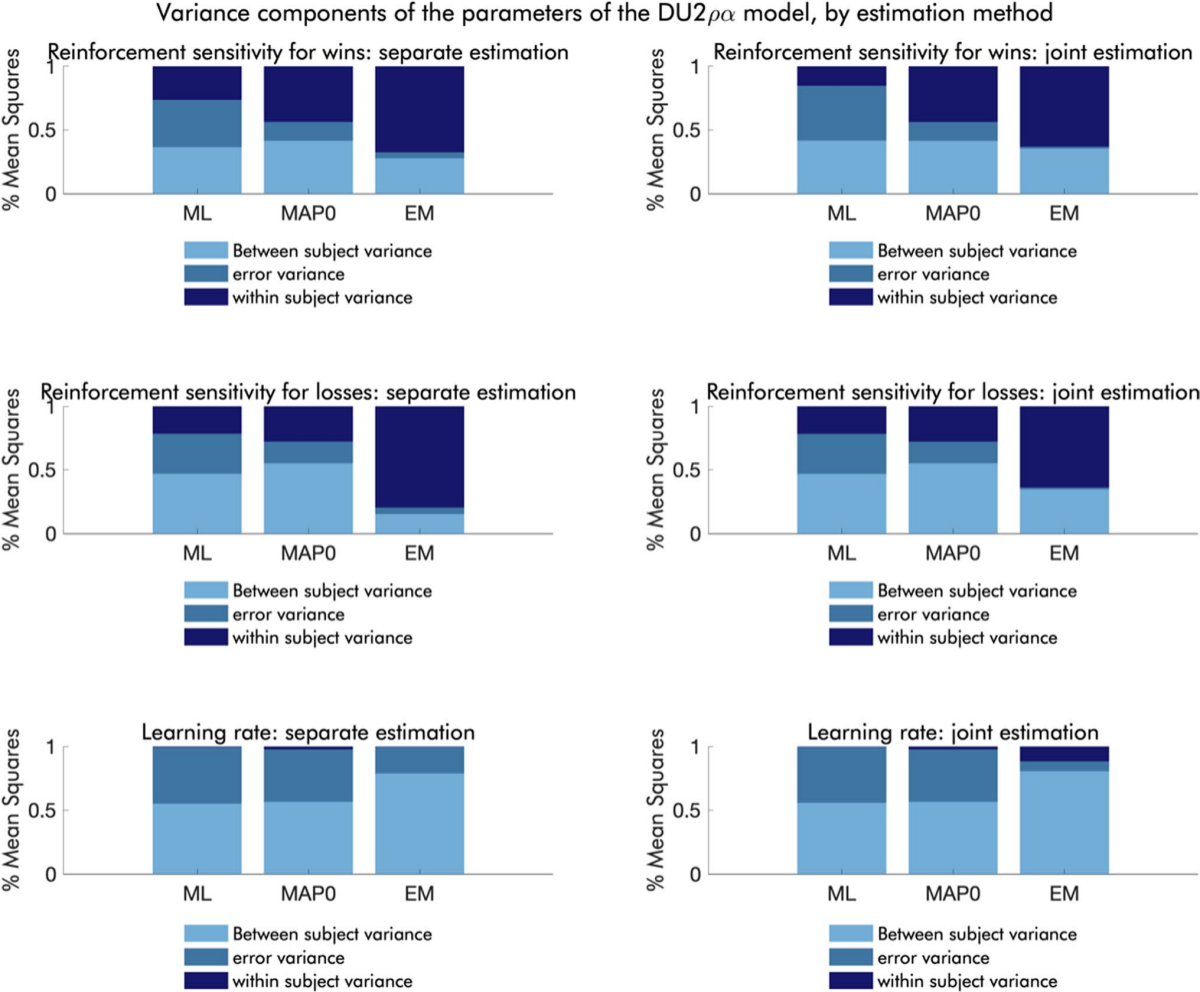
Relative variance components of the parameters derived from the DU-2𝜌𝛼 model, by estimation method and joint versus separate modeling of the two sessions. The left column reflects estimates based on separate modeling of the two sessions; the right column reflects estimates based on joint modeling of the two sessions. In each plot, the left bar represents maximum likelihood (ML) estimation, the middle bar represents estimates based on maximum a posteriori (MAP) estimation with uninformative priors (MAP0), and the right bar represents estimates based on MAP estimation with empirical priors (EM). Midnight blue sections of the bars reflect within-subject variance (session effects), steel blue sections of the bars reflect error variance, and sky blue sections of the bars reflect between-subject variance.

Partitioning the variance of the parameters from the DU-2𝜌𝛼 model into within-subject variance (systematic effects of session), between-subject variance, and error variance, we see that the relative amount of error variance decreases depending on the estimation method (Fig. 10). Thus, the error variance is largest for ML, smaller for MAP0, and smallest for EM. Additionally, we see a discernable effect of joint estimation on the relative amount of error variance in the EM estimates, such that it is smaller in parameters from the joint estimation.

#### Effect of analytically supplied gradients

Reliabilities were similar whether or not parameters were estimated using analytically supplied gradients, i.e., using a quasi-Newtonian or trust-region algorithm for optimization. In particular, they were exactly equivalent for the best-fitting model as well as a number of other models (Supplementary Tables 4 through 6). Variation was larger for ML and MAP0 estimates than EM-MAP estimates and for more complex models.

#### Recoverability

##### Behavior and parameters

**Fig. 11:**
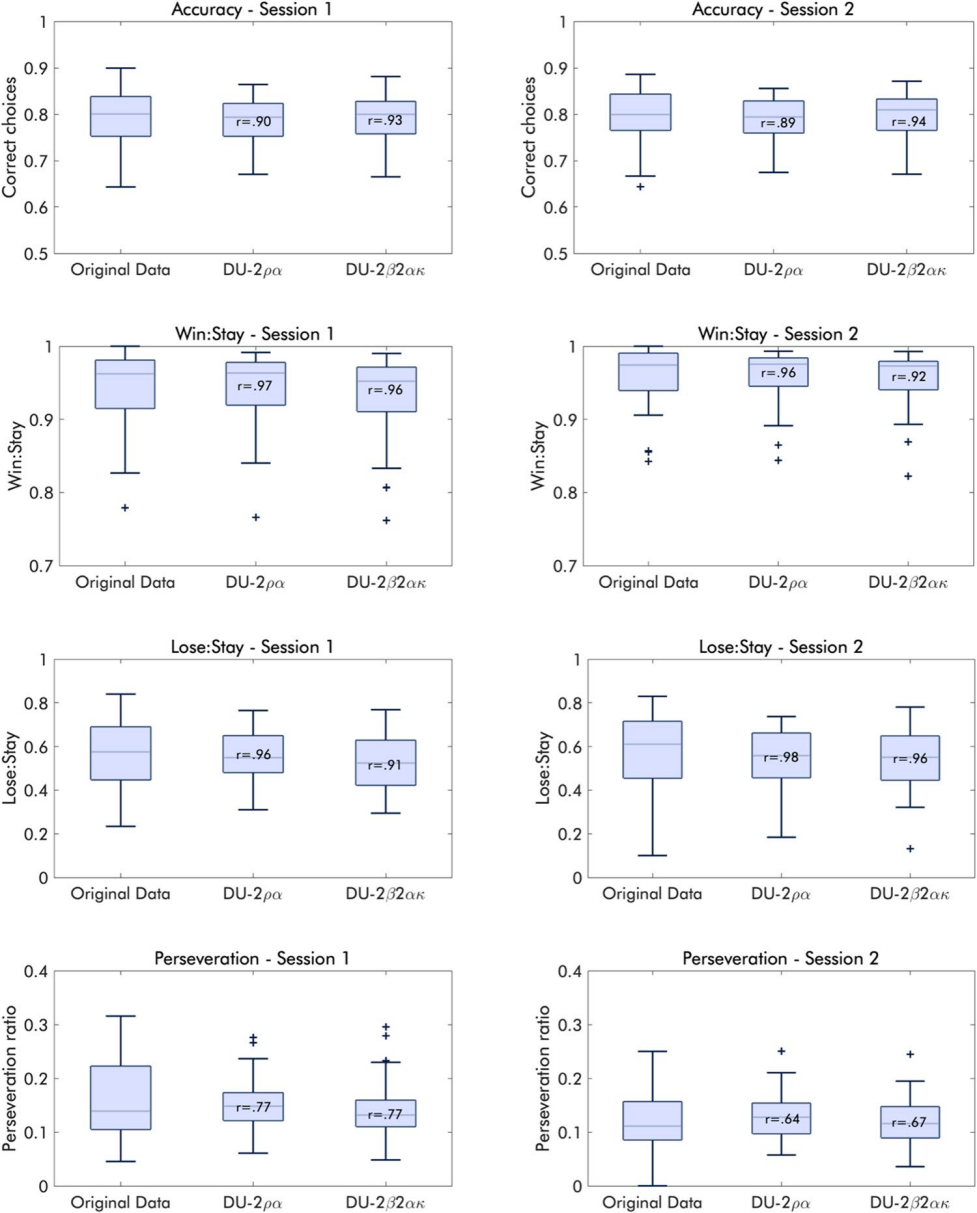
Distribution of the behavioral performance indices in the original sample and generated data based on the DU-2𝜌𝛼 and the DU-2𝛽2𝛼𝜅 models. Boxplots show the range, interquartile range, median, and outliers of the central performance indices of the PRLT; accuracy, win–stay probability, lose–stay probability, and the ratio of perseverative choices. The left column reflects the first session, the right column reflects the second session. Pearson correlation coefficients in each box representing model-generated data reflect the correlation with the original data.

Because the parameters estimated using EM and with both sessions modeled jointly produced the highest reliabilities, we took those estimates from the best-fitting models forward to probe them regarding their ability to reproduce the behavioral characteristics of the original sample. Both the DU-2𝜌𝛼 and the DU-2𝛽2𝛼𝜅 were able to reproduce behavior well on both the group and the individual level, albeit with somewhat less variance than the original data (Fig. 11). When refit to the generated data, the DU-2𝜌𝛼 showed very good recoverability, with high average correlations between the true parameters and the generated parameters (Table 1). The DU-2𝛽2𝛼𝜅 similarly showed good recoverability overall, albeit considerably less so for the learning rate for wins and the DU weight.

**Table 1:** Average correlation between true and recovered parameters

		Session 1	Session 2
DU-2𝜌𝛼	𝜌_win_	.93	.92
	𝜌_loss_	.80	.80
	𝛼	.76	.88
DU-2𝛽2𝛼𝜅	𝛽_win_	.82	.83
	𝛽_loss_	.79	.84
	𝛼_win_	.73	.55
	𝛼_loss_	.86	.87
	𝜅	.77	.66

##### Models

For completeness, we also performed model recovery. We simulated data from and refit a selection of other models from both families, covering different model complexities. These data are summarized in Supplementary Table 7 and Supplementary Fig. 2. Briefly, our simulations suggest that models with fewer parameters generally had better recoverability than more complex ones, and that models from the reinforcement sensitivity family generally had better recoverability than those from the softmax temperature family. This was expected, as both limiting the number of parameters and using a reinforcement sensitivity as opposed to a softmax temperature improves identifiability (Daw, 2011). Interestingly, they also indicate that separate learning rates for wins and losses might be particularly detrimental to recoverability. The DU-2𝛽2𝛼𝜅 model showed very poor model recoverability, suggesting that it might be hard to identify.

##### Reliability of recovered parameters and behavior

The recovered parameters reproduced the pattern of reliability we observed in our original parameters reasonably well. Thus, EM-MAP estimates were far more reliable than MAP0 and ML estimates. Likewise, the parameters derived from the DU-2𝜌𝛼 model were good to excellent and more reliable than those derived from the DU-2𝛽2𝛼𝜅 model (Table 2 for ICCs(A,1), Supplementary Tables 8 and 9 for ICCs(1) and model-calculated Pearson correlations).

**Table 2:** Mean ICC(A,1) between parameters for sessions 1 and 2, recovered using ML, MAP0, and EM-MAP estimation, based on 100 simulated datasets

		ML	MAP0	EM-MAP
DU-2𝜌𝛼	𝜌_win_	.27 [−.03–.53]	.64 [.37–.80]	.87 [.17–.96]
	𝜌_loss_	.18 [−.13–.46]	.30 [−.01–.56]	.77 [.38–.90]
	𝛼	.19 [−.11–.47]	.24 [−.07–.51]	.71 [.43–.85]
DU-2𝛽2𝛼𝜅	𝛽_win_	.00 [−.31–.32]	.07 [−.24–.37]	.64 [.33–.80]
	𝛽_loss_	.00 [−.32–.32]	.12 [−.19–.41]	.54 [.21–.74]
	𝛼_win_	−.04 [−.34–.27]	−.10 [−.39–.21]	−.18 [−.42–.13]
	𝛼_loss_	.29 [−.02–.55]	.48 [.20–.69]	.87 [.66–.93]
	𝜅	.11 [−.21–.40]	.19 [−.12–.47]	.38 [.08–.60]

Similarly, the behavioral metrics derived from data simulated on the basis of the EM-MAP estimates of the DU-2𝜌𝛼 model and the DU-2𝛽2𝛼𝜅 model showed comparable model-calculated reliability to the reliability of our original behavioral metrics (Table 3). However, the reliability of behavior simulated based on the DU-2𝜌𝛼 model was substantially closer to the reliability found in the original data. For other reliability metrics, see Supplementary Tables 10 through 12.

**Table 3:** Mean model-calculated ICC(1) between raw behavioral performance indices for sessions 1 and 2 based on 100 datasets simulated using the fit parameters of the DU-2𝜌𝛼 model and the DU-2𝛽2𝛼𝜅 model, respectively

	DU-2𝜌𝛼	DU-2𝛽2𝛼𝜅
Accuracy	0.51	0.61
Perseveration	0.82	0.73
Switching overall	0.84	0.72
Switching after losses	0.94	0.83
Switching after wins	0.73	0.6

## Discussion

Our results show that the version of PRLT we examined (Deserno et al., 2020; Reiter et al., 2016, 2017) can yield metrics with high internal consistency and mostly good to excellent retest reliability, on the level of both raw behavior and computational modeling. This supports past and future use of this task in neurocognitive research on inter-individual differences, in particular in psychiatry. It suggests that correlations with other measures such as symptom scales or functional magnetic resonance imaging (fMRI) data can be trusted (if their reliability is similarly high, which we discuss later). However, our data also indicate that this conclusion depends heavily on how the output of the task is processed, modeled, and estimated. Thus, our results suggest that modeling approaches that involve partial pooling of data across individuals and sessions yield the highest reliabilities. The use of analytically calculated gradients to aid the optimization had relatively little effect, such that dropping them would not have made a qualitative difference in our findings.

Our findings make a strong case for the superiority of using hierarchical methods to derive indices of task performance and their underlying processes. On the level of raw behavioral performance measures, we found that the reliability of predicted values derived from trial-level mixed-effects models tended to be slightly higher than of averages. On the level of parameters derived from computational modeling, we found that reliability benefitted strongly from estimation approaches that employed empirical group-level priors rather than no or uninformative priors. Moreover, we saw that the reliability of both raw behavioral performance measures and computational model parameters was enhanced if the data from both sessions were modeled jointly rather than separately. In other words, the reliability of estimates was higher when the estimation procedure involved partial pooling (i.e., when using mixed-effects models or empirical priors), and improved further as more data were available for pooling (i.e., when fitting the data from both sessions together). Given previous research investigating the reliability of cognitive tasks (Brown et al., 2020; Rouder & Haaf, 2019), this pattern is not surprising. Hierarchical estimation, including Bayesian estimation, produces regularized individual values. They are shrunk towards the group mean, which, intuitively, would cause alarm because it may reduce valuable between-subject variance and thus undermine reliability (Hedge et al., 2018). However, our results suggest that instead, shrinkage tends to cut away error variance (Efron & Morris, 1977) (see Figs. 3 and 10), thus improving reliability. Specifically, when partitioning variance, we see that the proportion of error variance is highest in estimates involving no shrinkage and lowest in those where estimates are shrunk towards a cross-subject, cross-session mean. In line with previous work (Brown et al., 2020; Rouder & Haaf, 2019), the effects on reliability were quite striking in our sample, suggesting that behavioral performance measures and especially parameters derived from computational modeling on this task may achieve adequate reliability *only* when estimated using hierarchical models that account for data from all sessions at once. There is, of course, a legitimate worry that models where data are pooled across sessions might produce single-session predictions that are biased to be similar to one another, and thus yield inflated reliability estimates. As we show in our simulations, this worry is partially warranted: reliabilities based on predicted values from joint session models do indeed tend to slightly overestimate true correlations. However, if the reliability estimates are instead based on variances that are calculated as part of the models themselves, and thus incorporate estimation uncertainty around point estimates, they tend to be accurate and manifest no obvious bias. This is in contrast to the reliabilities of means and predicted values from single-session models: our simulations show that these metrics tend to substantially underestimate true reliabilities and yield less accurate reliability estimates than the metrics based on joint models. This latter point was most evident when the true underlying correlations were high, suggesting that the regularization caused by the joint modeling had its most drastic effect in those cases. Since the reliabilities we calculated based on model-derived variance were only marginally lower than those based on the predicted values of the joint model, they confirm and underscore the great advantage of hierarchical approaches when it comes to obtaining reliable metrics.

Though theoretically compelling, this comes with caveats in practice. It is not an issue where hierarchical modeling can be easily accommodated, for example in longitudinal designs that investigate development or interventions. It becomes a little trickier in cross-sectional studies, where, it seems, the current number of trials in one run of the task may be insufficient to produce reliable estimates. However, the most difficult situation arises under the hypothetical condition that the task is used as a test or diagnostic tool in a single individual—here, neither group-level data nor a large number of trials are available to inform the estimation, which, based on our results, is problematic in particular for the reliability of computational modeling parameters. One straightforward solution could be to obtain a normative reference sample and extract empirical priors from it. This would aid both estimation and the contextualization of results. Reference samples are standard practice in differential psychology as part of test development for good reason, and perhaps should be in neurocognitive psychiatry.

In addition to the crucial role of the estimation method, our results suggest that identifiability may influence the reliability of computational model parameters. We saw that the model which best accounted for the data and produced the parameters with the highest reliability was a relatively simple model, where choice stochasticity was parameterized using reinforcement sensitivities rather than softmax temperatures. Both of these characteristics—being relatively simple and employing a reinforcement sensitivity rather that a softmax temperature—tend to improve identifiability: the first by reducing the number of parameters, the second by reducing collinearity between parameters (specifically between the choice stochasticity parameter(s) and the learning rate(s)) (Daw, 2011). By contrast, the softmax model with the best evidence was more complex and perhaps less easy to identify, as indicated by its poor model recoverability. Logically, it stands to reason that models that are easier to identify are also more reliable because the individual parameter estimates are less noisy (Daw, 2011). Indeed, we see diminished reliability for more complex models. This is somewhat disappointing, because many of the more “interesting” models contain a greater number of parameters. One example is models containing kappa, a parameter that captures the extent to which individuals learn by inference to the unchosen option. This parameter has often differed between psychiatric patients and controls in previous studies (e.g., Deserno et al., 2020; Reiter et al., 2016). In our sample, models containing kappa produced low(er)-reliability estimates. It is quite possible that because those models’ parameters are partially collinear with one another, they were more difficult to identify—at least on this task—and therefore less reliable. While we cannot prove that this *causes* low reliability in our particular instance, it is an important reminder that identifiability should be ensured for any model whose parameters are to be used for interindividual difference research.

The reason that we cannot prove that poor identifiability caused low reliability in our complex models is that we cannot perfectly differentiate between the effects of identifiability and goodness of fit. They tend to coincide, so that some simpler models also fit the data better. This highlights a different, though equally important, problem complex models may have: parameters that do not capture meaningful variance in the data will hardly be reliable, even if they are perfectly identifiable. This could well have been an issue with some of our DU models, in particular within the softmax family. Healthy individuals usually perform rather well on the task and win frequently, so the loss-specific parameters may mop up much of the variance usually associated with double updating, causing poorer fit for models containing kappa. Though somewhat sobering, this is not necessarily a quietus for the investigation of the more intricate processes that some complex models reflect—it merely suggests that the specific PRLT we focused on might not be ideal to probe them. It is conceivable that tasks can be designed so as to orthogonalize the parameters to a more workable extent, and it may be well worth it if it comes with the added benefit of sufficient reliability.

Though we did not empirically show this, it is important to stress that even given high reliability of the PRLT, associations with other measures such as symptom scores and neuroimaging data will always depend to an equal degree on the reliability of those measures (Hedge et al., 2018). Indeed, the reliability of readouts from important imaging techniques such as fMRI are somewhat contentious. For example, a recent meta-analysis showed that the mean reliability of task-related blood oxygen level-dependent (BOLD) effects is rather poor (Elliott et al., 2020) (ICC(C,1) = .397). However, the authors suggest that this is not due to inherently inadequate reliability of the BOLD signal, but rather poor task reliability (Elliott et al., 2020). Given its high reliability, the PRLT thus seems to be a promising candidate for eliciting reliable BOLD effects, although this is yet to be shown. Regardless, special care should be taken with model-based fMRI readouts, which Elliott et al. (2020) did not consider. These come with their own pitfalls. It has been shown, on different tasks, that regressors derived from individually fit parameters from RL models—e.g., learning rates—can produce robust BOLD effects even if the model is poorly fit (Wilson & Niv, 2015). But, as Katahira et al. (2021) show, if parameter values are systematically over- or underestimated depending on group memberships (e.g., patients and controls) or a symptom scale, spurious differences in BOLD effects are likely to emerge, even if they are in fact similar across groups (Katahira & Toyama, 2021). More worrying still, even when parameters are perfectly fit, the magnitude of the BOLD response associated with a parameter-derived regressor can, under some circumstances, depend on the regressor’s variance—which, in turn, depends on the value the parameter takes (Katahira & Toyama, 2021; Lebreton et al., 2019). If parameter values are associated with symptom severity, say, this can again cause spurious effects. Therefore, the PRLT should be scrutinized in terms of the consequences that task design, model specification, parameter values, and data preprocessing might have on BOLD responses beyond the performance differences of interest. Ideally, the reliability and validity of PRLT-related BOLD responses should be ascertained in a bespoke study before interindividual differences in its neural correlates can be interpreted with confidence.

## Limitations

Because we used a small sample of younger and healthy individuals, it is unclear whether our results extend to more diverse populations. This is particularly relevant concerning individuals with psychopathologies, the target population of much research employing the PRLT. However, more homogeneous samples such as ours should theoretically have lower reliability due to lower between-subject variability. We would therefore expect to find even higher reliability in more heterogeneous samples. Similarly, it is unclear whether our findings generalize to other versions of the PRLT, with different rules for reversals occurring, as well as other RL tasks. It will be important to compare several versions in more diverse populations in order to tease out the best available instruments to investigate flexible behavioral adaptation. Finally, our version of the task was set up in such a way as to produce variable numbers of probabilistic events across participants and sessions. This means that for some participants, there were slight differences in task difficulty across sessions. Future investigations may further improve reliability by holding difficulty constant across sessions.

## Conclusion

In sum, our study indicates that the version of the PRLT we examined has suitable reliability to be employed in clinical research. Crucially, we show that employing hierarchical estimation has enormous benefits. In light of previous research that came to similar conclusions with respect to other tasks (Brown et al., 2020; Rouder & Haaf, 2019), we therefore recommend that this be the default approach. We further found that complex computational models had lower reliability than simpler ones; however, it is less clear whether this is due to poorer fit of these models or problems with identifiability. Nonetheless, we suggest adjusting models to reduce collinearity between parameters as much as possible to avoid noisy estimates. For more intricate neurocognitive processes, we encourage researchers to design tasks that explicitly manipulate those and emphasize that task design and optimization may benefit from using computational tools a priori.

## Supplementary Information

ESM 1 (DOCX 692 kb)

## Declarations

### Availability of data and materials

The raw data underlying this article are available on the Open Science Framework (osf.io/4ng3e).

### Code availability

The analysis scripts underlying the analyses in this article are available on the Open Science Framework (osf.io/4ng3e).

### Preregistration

This study was not preregistered.
