# Supplementary material for: Sufficient reliability of the behavioral and computational readouts of a probabilistic reversal learning task
Source: Behav Res Methods. 2022 Feb 15;54(6):2993–3014. doi: 10.3758/s13428-021-01739-7 (PMC9729159; doi:10.3758/s13428-021-01739-7)
Supplement: Supplementary file 1 — (DOCX 692 kb) [file 13428_2021_1739_MOESM1_ESM.docx]

# SUPPLEMENTARY MATERIAL

| **Supplementary Table 1** Retest reliability [ICC(A,1)] of the parameters of all computational models | | | | | | | |
| --- | --- | --- | --- | --- | --- | --- | --- |
|  |  | **ML** | | **MAP0** | | **EM-MAP** | |
|  |  | **Separate** | **Joint** | **Separate** | **Joint** | **Separate** | **Joint** |
| SU-𝜌𝛼 | 𝜌 | 0 | 0 | 0.26 | 0.26 | 0.42 | 0.58 |
|  |  | [−0.31–0.31] | [−0.32–0.32] | [−0.05–0.52] | [−0.05–0.52] | [0.14–0.65] | [0.32–0.76] |
|  | 𝛼 | 0.22 | 0.45 | 0.3 | 0.3 | 0.32 | 0.38 |
|  |  | [−0.11–0.51] | [0.15–0.67] | [−0.02–0.56] | [−0.02–0.56] | [0–0.58] | [0.08–0.63] |
| SU-2𝜌𝛼 | 𝜌_win_ | 0.01 | 0.01 | 0.26 | 0.26 | 0.53 | 0.7 |
|  |  | [−0.31–0.32] | [−0.29–0.32] | [−0.03–0.52] | [−0.03–0.52] | [0.21–0.74] | [0.26–0.87] |
|  | 𝜌_loss_ | −0.04 | −0.03 | 0.34 | 0.34 | 0.38 | 0.88 |
|  |  | [−0.36–0.29] | [−0.35–0.29] | [0.02–0.59] | [0.02–0.59] | [0.08–0.62] | [0.76–0.94] |
|  | 𝛼 | 0.03 | 0.11 | 0.33 | 0.33 | 0.26 | 0.37 |
|  |  | [−0.3–0.35] | [−0.22–0.42] | [0.02–0.59] | [0.02–0.59] | [−0.06–0.53] | [0.06–0.62] |
| SU-𝜌2𝛼 | 𝜌 | −0.06 | −0.04 | −0.07 | 0.41 | 0.57 | 0.76 |
|  |  | [−0.37–0.26] | [−0.36–0.28] | [−0.38–0.25] | [0.12–0.64] | [0.31–0.75] | [0.59–0.87] |
|  | 𝛼_win_ | 0.1 | 0.15 | −0.23 | 0.02 | −0.05 | 0.03 |
|  |  | [−0.23–0.41] | [−0.17–0.45] | [−0.52–0.1] | [−0.3–0.34] | [−0.33–0.25] | [−0.24–0.31] |
|  | 𝛼_loss_ | 0.27 | 0.41 | 0.31 | 0.38 | 0.4 | 0.48 |
|  |  | [−0.06–0.54] | [0.1–0.64] | [−0.01–0.57] | [0.07–0.62] | [0.09–0.64] | [0.19–0.69] |
| SU-2𝜌2𝛼 | 𝜌_win_ | 0 | −0.07 | 0.12 | 0.12 | 0.51 | 0.58 |
|  |  | [−0.31–0.31] | [−0.38–0.26] | [−0.18–0.4] | [−0.17–0.41] | [0.24–0.71] | [0.33–0.76] |
|  | 𝜌_loss_ | −0.08 | −0.05 | 0.39 | 0.36 | 0.26 | 0.66 |
|  |  | [−0.4–0.25] | [−0.36–0.27] | [0.08–0.63] | [0.04–0.61] | [−0.07–0.53] | [0.44–0.81] |
|  | 𝛼_win_ | −0.1 | −0.06 | 0.1 | 0.05 | 0.13 | −0.04 |
|  |  | [−0.42–0.23] | [−0.38–0.27] | [−0.22–0.4] | [−0.27–0.36] | [−0.19–0.43] | [−0.34–0.28] |
|  | 𝛼_loss_ | 0.19 | 0.2 | 0.52 | 0.44 | 0.68 | 0.84 |
|  |  | [−0.14–0.48] | [−0.13–0.49] | [0.25–0.72] | [0.14–0.66] | [0.47–0.82] | [0.7–0.91] |
| DU-𝜌𝛼 | 𝜌 | 0.61 | 0.63 | 0.61 | 0.61 | 0.61 | 0.74 |
|  |  | [0.36–0.78] | [0.36–0.8] | [0.36–0.78] | [0.36–0.78] | [0.34–0.78] | [0.48–0.87] |
|  | 𝛼 | 0.58 | 0.69 | 0.58 | 0.58 | 0.58 | 0.66 |
|  |  | [0.32–0.76] | [0.48–0.83] | [0.32–0.76] | [0.32–0.76] | [0.32–0.75] | [0.44–0.81] |
| DU-2𝜌𝛼 | 𝜌_win_ | −0.01 | −0.02 | 0.46 | 0.46 | 0.64 | 0.85 |
|  |  | [−0.33–0.31] | [−0.34–0.3] | [0.18–0.68] | [0.18–0.68] | [0.31–0.82] | [0.26–0.95] |
|  | 𝜌_loss_ | 0.2 | 0.2 | 0.52 | 0.52 | 0.42 | 0.84 |
|  |  | [−0.13–0.48] | [−0.13–0.48] | [0.25–0.72] | [0.25–0.72] | [0.08–0.66] | [0.25–0.95] |
|  | 𝛼 | 0.16 | 0.17 | 0.2 | 0.2 | 0.59 | 0.83 |
|  |  | [−0.17–0.46] | [−0.16–0.46] | [−0.13–0.49] | [−0.13–0.49] | [0.33–0.76] | [0.69–0.91] |
| DU-𝜌2𝛼 | 𝜌 | −0.03 | −0.02 | 0.34 | 0.45 | 0.44 | 0.84 |
|  |  | [−0.35–0.29] | [−0.35–0.3] | [0.03–0.59] | [0.15–0.67] | [0.14–0.67] | [0.72–0.92] |
|  | 𝛼_win_ | 0.38 | −0.06 | 0.33 | 0.35 | 0.57 | 0.04 |
|  |  | [0.08–0.62] | [−0.38–0.27] | [0.02–0.58] | [0.04–0.6] | [0.31–0.75] | [−0.16–0.28] |
|  | 𝛼_loss_ | 0.18 | 0.25 | 0.21 | 0.29 | −0.19 | 0.41 |
|  |  | [−0.14–0.47] | [−0.08–0.53] | [−0.12–0.49] | [−0.04–0.55] | [−0.49–0.14] | [0.09–0.65] |
| DU-2𝜌2𝛼 | 𝜌_win_ | 0.14 | 0.15 | 0.48 | 0.48 | 0.5 | 0.91 |
|  |  | [−0.2–0.44] | [−0.18–0.45] | [0.2–0.69] | [0.2–0.69] | [0.21–0.7] | [0.83–0.95] |
|  | 𝜌_loss_ | 0.35 | 0.28 | 0.49 | 0.49 | 0.08 | 0.89 |
|  |  | [0.03–0.6] | [−0.04–0.55] | [0.2–0.7] | [0.2–0.7] | [−0.25–0.39] | [0.79–0.94] |
|  | 𝛼_win_ | −0.22 | 0.12 | 0.24 | −0.18 | 0.07 | 0.03 |
|  |  | [−0.51–0.12] | [−0.21–0.42] | [−0.09–0.52] | [−0.49–0.15] | [−0.12–0.31] | [−0.12–0.22] |
|  | 𝛼_loss_ | 0.1 | 0.03 | 0.11 | 0.36 | 0.14 | 0.67 |
|  |  | [−0.22–0.4] | [−0.29–0.35] | [−0.22–0.42] | [0.05–0.61] | [−0.06–0.43] | [−0.08–0.9] |
| DU-𝜌𝛼𝜅 | 𝜌 | −0.02 | 0.19 | 0.53 | 0.53 | 0.57 | 0.72 |
|  |  | [−0.33–0.29] | [−0.12–0.47] | [0.26–0.72] | [0.26–0.72] | [0.3–0.75] | [0.46–0.85] |
|  | 𝛼 | 0.41 | 0.37 | 0.36 | 0.36 | 0.41 | 0.43 |
|  |  | [0.11–0.64] | [0.06–0.62] | [0.04–0.61] | [0.04–0.61] | [0.11–0.64] | [0.14–0.65] |
|  | 𝜅 | 0.41 | 0.26 | 0.28 | 0.28 | 0.45 | 0.78 |
|  |  | [0.11–0.64] | [−0.07–0.53] | [−0.04–0.55] | [−0.04–0.55] | [0.16–0.67] | [0.56–0.89] |
| DU-2𝜌𝛼𝜅 | 𝜌_win_ | −0.73 | 0.02 | 0.43 | 0.42 | 0.65 | 0.84 |
|  |  | [−0.87 to −0.5] | [−0.3–0.33] | [0.14–0.65] | [0.13–0.64] | [0.36–0.81] | [0.13–0.95] |
|  | 𝜌_loss_ | −0.7 | 0.01 | 0.4 | 0.48 | 0.47 | 0.94 |
|  |  | [−0.86 to −0.45] | [−0.31–0.32] | [0.09–0.64] | [0.19–0.69] | [0.18–0.68] | [0.4–0.98] |
|  | 𝛼 | 0.27 | 0.24 | 0.32 | 0.33 | 0.27 | 0.6 |
|  |  | [−0.05–0.55] | [−0.09–0.52] | [0–0.58] | [0.01–0.59] | [−0.03–0.53] | [0.33–0.78] |
|  | 𝜅 | 0.05 | −0.02 | 0.13 | 0 | −0.19 | −0.57 |
|  |  | [−0.27–0.37] | [−0.34–0.3] | [−0.2–0.43] | [−0.33–0.32] | [−0.48–0.14] | [−0.77 to −0.29] |
| DU-𝜌2𝛼𝜅 | 𝜌 | −0.07 | 0.08 | 0.34 | 0.5 | 0.5 | 0.69 |
|  |  | [−0.39–0.26] | [−0.25–0.39] | [0.02–0.59] | [0.21–0.7] | [0.23–0.71] | [0.47–0.83] |
|  | 𝛼_win_ | 0.21 | −0.16 | 0.01 | 0.1 | −0.03 | 0.16 |
|  |  | [−0.13–0.49] | [−0.46–0.17] | [−0.31–0.33] | [−0.23–0.41] | [−0.34–0.28] | [−0.17–0.45] |
|  | 𝛼_loss_ | 0.39 | 0.11 | 0.25 | 0.24 | 0.59 | 0.73 |
|  |  | [0.08–0.63] | [−0.22–0.42] | [−0.08–0.52] | [−0.09–0.52] | [0.34–0.76] | [0.54–0.85] |
|  | 𝜅 | 0.42 | 0.22 | 0.19 | 0.55 | 0.45 | 0.72 |
|  |  | [0.11–0.65] | [−0.08–0.5] | [−0.12–0.48] | [0.29–0.74] | [0.15–0.67] | [0.53–0.84] |
| DU-2𝜌2𝛼𝜅 | 𝜌_win_ | 0.1 | 0.2 | 0.47 | 0.48 | 0.55 | 0.88 |
|  |  | [−0.22–0.4] | [−0.14–0.49] | [0.19–0.68] | [0.2–0.69] | [0.28–0.73] | [0.48–0.95] |
|  | 𝜌_loss_ | −0.1 | −0.04 | 0.29 | 0.29 | 0.03 | 0.91 |
|  |  | [−0.4–0.23] | [−0.35–0.28] | [−0.03–0.56] | [−0.03–0.56] | [−0.29–0.35] | [0.79–0.96] |
|  | 𝛼_win_ | 0.18 | 0.21 | 0.1 | 0.29 | 0.04 | −0.12 |
|  |  | [−0.14–0.47] | [−0.11–0.49] | [−0.23–0.41] | [−0.03–0.56] | [−0.29–0.36] | [−0.38–0.19] |
|  | 𝛼_loss_ | 0.25 | 0.22 | 0.26 | 0.31 | 0.43 | 0.95 |
|  |  | [−0.08–0.52] | [−0.11–0.51] | [−0.06–0.53] | [−0.01–0.57] | [0.13–0.65] | [0.91–0.98] |
|  | 𝜅 | −0.22 | 0.22 | −0.02 | 0 | 0.05 | 0.33 |
|  |  | [−0.5–0.11] | [−0.1–0.51] | [−0.33–0.3] | [−0.32–0.32] | [−0.28–0.36] | [0.03–0.58] |
| SU-𝛽𝛼 | 𝛽 | 0 | 0 | 0.26 | 0.26 | 0.42 | 0.58 |
|  |  | [−0.31–0.31] | [−0.32–0.32] | [−0.05–0.52] | [−0.05–0.52] | [0.14–0.65] | [0.32–0.76] |
|  | 𝛼 | 0.3 | 0.16 | 0.3 | 0.3 | 0.32 | 0.38 |
|  |  | [−0.02–0.57] | [−0.17–0.45] | [−0.02–0.56] | [−0.02–0.56] | [0–0.58] | [0.08–0.63] |
| SU-2𝛽𝛼 | 𝛽_win_ | −0.02 | 0 | −0.03 | −0.03 | 0.29 | 0.34 |
|  |  | [−0.35–0.3] | [−0.29–0.3] | [−0.35–0.29] | [−0.35–0.29] | [−0.02–0.55] | [0.04–0.59] |
|  | 𝛽_loss_ | 0 | −0.03 | 0.04 | 0.04 | 0.47 | 0.55 |
|  |  | [−0.32–0.32] | [−0.35–0.29] | [−0.28–0.35] | [−0.28–0.35] | [0.18–0.68] | [0.28–0.74] |
|  | 𝛼 | 0.13 | 0.11 | 0.16 | 0.16 | 0.28 | 0.34 |
|  |  | [−0.2–0.43] | [−0.22–0.41] | [−0.17–0.46] | [−0.17–0.46] | [−0.04–0.55] | [0.03–0.59] |
| SU-𝛽2𝛼 | 𝛽 | 0 | 0 | −0.05 | 0.13 | 0.49 | 0.74 |
|  |  | [−0.32–0.32] | [−0.32–0.32] | [−0.35–0.26] | [−0.17–0.42] | [0.21–0.7] | [0.56–0.86] |
|  | 𝛼_win_ | 0.03 | 0.03 | −0.1 | 0.02 | −0.05 | 0.03 |
|  |  | [−0.29–0.35] | [−0.29–0.35] | [−0.41–0.23] | [−0.3–0.34] | [−0.33–0.25] | [−0.24–0.31] |
|  | 𝛼_loss_ | 0.37 | 0.37 | 0.34 | 0.38 | 0.4 | 0.48 |
|  |  | [0.05–0.61] | [0.05–0.61] | [0.03–0.6] | [0.07–0.62] | [0.09–0.64] | [0.19–0.69] |
| SU-2𝛽2𝛼 | 𝛽_win_ | 0 | 0 | −0.03 | −0.05 | 0.42 | 0.43 |
|  |  | [−0.32–0.32] | [−0.32–0.32] | [−0.33–0.29] | [−0.37–0.27] | [0.13–0.64] | [0.14–0.66] |
|  | 𝛽_loss_ | −0.03 | −0.01 | −0.05 | 0.06 | 0.54 | 0.71 |
|  |  | [−0.36–0.29] | [−0.32–0.3] | [−0.37–0.27] | [−0.27–0.37] | [0.27–0.73] | [0.51–0.84] |
|  | 𝛼_win_ | 0.12 | −0.02 | 0.1 | 0.03 | 0.06 | 0.01 |
|  |  | [−0.21–0.43] | [−0.34–0.3] | [−0.22–0.4] | [−0.29–0.35] | [−0.23–0.35] | [−0.29–0.32] |
|  | 𝛼_loss_ | 0.13 | 0.14 | 0.22 | 0.17 | 0.4 | 0.48 |
|  |  | [−0.2–0.43] | [−0.19–0.44] | [−0.11–0.51] | [−0.17–0.46] | [0.09–0.64] | [0.19–0.69] |
| DU-𝛽𝛼 | 𝛽 | 0.52 | 0.52 | 0.52 | 0.52 | 0.54 | 0.7 |
|  |  | [0.24–0.71] | [0.24–0.71] | [0.25–0.72] | [0.25–0.72] | [0.26–0.74] | [0.41–0.85] |
|  | 𝛼 | 0.58 | 0.58 | 0.58 | 0.58 | 0.58 | 0.66 |
|  |  | [0.32–0.76] | [0.32–0.76] | [0.32–0.76] | [0.32–0.76] | [0.32–0.75] | [0.44–0.81] |
| DU-2𝛽𝛼 | 𝛽_win_ | −0.03 | 0 | 0.01 | 0.02 | 0.52 | 0.68 |
|  |  | [−0.34–0.29] | [−0.32–0.32] | [−0.32–0.33] | [−0.31–0.34] | [0.24–0.72] | [0.42–0.83] |
|  | 𝛽_loss_ | 0.56 | 0.54 | 0.29 | 0.46 | 0.54 | 0.66 |
|  |  | [0.29–0.74] | [0.27–0.73] | [−0.02–0.55] | [0.18–0.68] | [0.27–0.73] | [0.41–0.81] |
|  | 𝛼 | 0.46 | 0.36 | 0.02 | 0.17 | 0.54 | 0.64 |
|  |  | [0.17–0.67] | [0.05–0.61] | [−0.31–0.34] | [−0.16–0.46] | [0.27–0.73] | [0.41–0.8] |
| DU-𝛽2𝛼 | 𝛽 | 0 | −0.04 | 0.04 | 0.12 | 0.33 | 0.86 |
|  |  | [−0.32–0.32] | [−0.35–0.28] | [−0.27–0.35] | [−0.2–0.42] | [0.01–0.59] | [0.74–0.92] |
|  | 𝛼_win_ | 0.28 | 0.2 | 0.07 | 0.38 | 0.32 | −0.09 |
|  |  | [−0.04–0.54] | [−0.13–0.49] | [−0.26–0.39] | [0.07–0.62] | [0.01–0.58] | [−0.4–0.23] |
|  | 𝛼_loss_ | 0.46 | 0.18 | 0.13 | 0.41 | 0.18 | 0.26 |
|  |  | [0.17–0.67] | [−0.15–0.47] | [−0.2–0.43] | [0.1–0.64] | [−0.08–0.44] | [−0.03–0.52] |
| DU-2𝛽2𝛼 | 𝛽_win_ | 0 | 0 | 0.08 | 0.1 | 0.28 | 0.87 |
|  |  | [−0.32–0.32] | [−0.31–0.31] | [−0.24–0.39] | [−0.22–0.4] | [−0.04–0.55] | [0.76–0.93] |
|  | 𝛽_loss_ | 0 | 0 | 0.08 | 0.09 | 0.4 | 0.75 |
|  |  | [−0.32–0.32] | [−0.32–0.32] | [−0.25–0.39] | [−0.24–0.4] | [0.1–0.63] | [0.57–0.86] |
|  | 𝛼_win_ | 0.44 | −0.08 | 0.48 | 0.14 | −0.04 | 0.5 |
|  |  | [0.15–0.66] | [−0.4–0.25] | [0.2–0.69] | [−0.18–0.44] | [−0.33–0.27] | [0.16–0.72] |
|  | 𝛼_loss_ | 0.33 | 0.4 | 0.23 | 0.15 | 0.6 | 0.74 |
|  |  | [0.01–0.58] | [0.1–0.64] | [−0.1–0.51] | [−0.17–0.44] | [0.35–0.77] | [0.55–0.85] |
| DU-𝛽𝛼𝜅 | 𝛽 | 0 | 0 | 0.36 | 0.41 | 0.49 | 0.78 |
|  |  | [−0.32–0.32] | [−0.32–0.31] | [0.07–0.61] | [0.12–0.64] | [0.21–0.7] | [0.55–0.89] |
|  | 𝛼 | 0.37 | 0.36 | 0.36 | 0.46 | 0.41 | 0.7 |
|  |  | [0.06–0.61] | [0.05–0.61] | [0.04–0.61] | [0.17–0.68] | [0.11–0.64] | [0.42–0.84] |
|  | 𝜅 | 0.26 | 0.21 | 0.28 | 0.29 | 0.45 | 0.68 |
|  |  | [−0.07–0.53] | [−0.11–0.5] | [−0.04–0.55] | [−0.03–0.55] | [0.16–0.67] | [0.42–0.83] |
| DU-2𝛽𝛼𝜅 | 𝛽_win_ | 0 | 0 | 0 | 0 | 0.46 | 0.56 |
|  |  | [−0.31–0.31] | [−0.3–0.31] | [−0.32–0.32] | [−0.32–0.32] | [0.17–0.67] | [0.3–0.74] |
|  | 𝛽_loss_ | 0 | 0 | 0.26 | 0.26 | 0.42 | 0.38 |
|  |  | [−0.32–0.32] | [−0.32–0.32] | [−0.05–0.52] | [−0.05–0.52] | [0.12–0.65] | [0.09–0.62] |
|  | 𝛼 | 0.2 | 0.13 | 0.2 | 0.2 | 0.35 | 0.28 |
|  |  | [−0.13–0.48] | [−0.2–0.43] | [−0.13–0.49] | [−0.13–0.49] | [0.05–0.59] | [−0.01–0.54] |
|  | 𝜅 | 0.27 | 0.15 | 0.19 | 0.19 | 0.38 | 0.44 |
|  |  | [−0.04–0.54] | [−0.16–0.44] | [−0.13–0.47] | [−0.13–0.47] | [0.08–0.62] | [−0.03–0.72] |
| DU-𝛽2𝛼𝜅 | 𝛽 | 0 | −0.02 | 0.09 | 0.15 | 0.36 | 0.78 |
|  |  | [−0.32–0.31] | [−0.34–0.3] | [−0.25–0.4] | [−0.18–0.45] | [0.05–0.61] | [0.62–0.88] |
|  | 𝛼_win_ | 0.09 | −0.04 | 0.11 | 0.11 | −0.03 | 0.41 |
|  |  | [−0.24–0.4] | [−0.36–0.29] | [−0.23–0.41] | [−0.21–0.42] | [−0.34–0.28] | [0.12–0.64] |
|  | 𝛼_loss_ | 0.34 | −0.15 | 0.38 | 0.4 | 0.59 | 0.77 |
|  |  | [0.02–0.59] | [−0.46–0.18] | [0.07–0.62] | [0.09–0.64] | [0.34–0.76] | [0.61–0.88] |
|  | 𝜅 | 0.35 | 0.29 | 0.21 | 0.22 | 0.45 | 0.2 |
|  |  | [0.04–0.6] | [−0.01–0.56] | [−0.09–0.49] | [−0.1–0.5] | [0.15–0.67] | [−0.12–0.49] |
| DU-2𝛽2𝛼𝜅 | 𝛽_win_ | 0 | 0 | 0.17 | −0.03 | 0.39 | 0.75 |
|  |  | [−0.32–0.32] | [−0.31–0.31] | [−0.15–0.45] | [−0.35–0.29] | [0.1–0.63] | [0.54–0.87] |
|  | 𝛽_loss_ | 0 | 0 | 0.41 | 0.15 | 0.39 | 0.46 |
|  |  | [−0.31–0.31] | [−0.32–0.32] | [0.12–0.64] | [−0.18–0.45] | [0.09–0.62] | [0.15–0.68] |
|  | 𝛼_win_ | 0.08 | 0.27 | −0.13 | 0.04 | 0 | −0.35 |
|  |  | [−0.25–0.39] | [−0.06–0.54] | [−0.44–0.2] | [−0.28–0.36] | [−0.32–0.33] | [−0.61 to −0.01] |
|  | 𝛼_loss_ | −0.18 | −0.04 | 0.05 | 0.06 | 0.49 | 0.95 |
|  |  | [−0.48–0.15] | [−0.36–0.28] | [−0.28–0.36] | [−0.27–0.38] | [0.21–0.7] | [0.91–0.97] |
|  | 𝜅 | 0.21 | 0.05 | 0.22 | 0.24 | 0.54 | 0.46 |
|  |  | [−0.1–0.49] | [−0.23–0.34] | [−0.09–0.5] | [−0.06–0.51] | [0.27–0.73] | [0.11–0.7] |

| **Supplementary Table 2** Retest reliability [ICC(1)] of the parameters of all computational models | | | | | | | |
| --- | --- | --- | --- | --- | --- | --- | --- |
|  |  | **ML** | | **MAP0** | | **EM-MAP** | |
|  |  | **Separate** | **Joint** | **Separate** | **Joint** | **Separate** | **Joint** |
| SU-𝜌𝛼 | 𝜌 | 0 | 0 | 0.24 | 0.24 | 0.41 | 0.57 |
|  |  | [−0.32–0.31] | [−0.31–0.31] | [−0.08–0.52] | [−0.08–0.52] | [0.11–0.64] | [0.31–0.75] |
|  | 𝛼 | 0.23 | 0.45 | 0.31 | 0.31 | 0.33 | 0.39 |
|  |  | [−0.09–0.51] | [0.16–0.67] | [−0.01–0.57] | [−0.01–0.57] | [0.01–0.58] | [0.08–0.63] |
| SU-2𝜌𝛼 | 𝜌_win_ | 0 | −0.01 | 0.23 | 0.23 | 0.51 | 0.68 |
|  |  | [−0.31–0.32] | [−0.32–0.31] | [−0.09–0.51] | [−0.09–0.51] | [0.23–0.71] | [0.47–0.82] |
|  | 𝜌_loss_ | −0.02 | −0.02 | 0.34 | 0.34 | 0.37 | 0.88 |
|  |  | [−0.33–0.3] | [−0.33–0.29] | [0.03–0.59] | [0.03–0.59] | [0.06–0.61] | [0.78–0.93] |
|  | 𝛼 | 0.04 | 0.12 | 0.34 | 0.34 | 0.27 | 0.37 |
|  |  | [−0.28–0.35] | [−0.2–0.42] | [0.03–0.59] | [0.03–0.59] | [−0.05–0.54] | [0.07–0.62] |
| SU-𝜌2𝛼 | 𝜌 | −0.06 | −0.04 | −0.08 | 0.4 | 0.56 | 0.76 |
|  |  | [−0.36–0.26] | [−0.35–0.28] | [−0.38–0.24] | [0.1–0.64] | [0.3–0.75] | [0.58–0.87] |
|  | 𝛼_win_ | 0.11 | 0.15 | −0.21 | 0.03 | −0.09 | −0.04 |
|  |  | [−0.21–0.41] | [−0.17–0.45] | [−0.49–0.11] | [−0.29–0.34] | [−0.39–0.23] | [−0.35–0.28] |
|  | 𝛼_loss_ | 0.27 | 0.41 | 0.32 | 0.39 | 0.4 | 0.48 |
|  |  | [−0.04–0.54] | [0.12–0.64] | [0.01–0.58] | [0.08–0.62] | [0.1–0.64] | [0.2–0.69] |
| SU-2𝜌2𝛼 | 𝜌_win_ | −0.02 | −0.06 | 0.08 | 0.09 | 0.51 | 0.58 |
|  |  | [−0.33–0.3] | [−0.37–0.26] | [−0.24–0.39] | [−0.23–0.4] | [0.23–0.71] | [0.32–0.76] |
|  | 𝜌_loss_ | −0.07 | −0.05 | 0.4 | 0.36 | 0.27 | 0.66 |
|  |  | [−0.37–0.25] | [−0.36–0.26] | [0.09–0.63] | [0.05–0.61] | [−0.05–0.54] | [0.44–0.81] |
|  | 𝛼_win_ | −0.08 | −0.05 | 0.1 | 0.06 | 0.13 | −0.05 |
|  |  | [−0.39–0.24] | [−0.35–0.27] | [−0.22–0.4] | [−0.26–0.36] | [−0.19–0.42] | [−0.36–0.27] |
|  | 𝛼_loss_ | 0.2 | 0.21 | 0.53 | 0.44 | 0.68 | 0.83 |
|  |  | [−0.12–0.49] | [−0.11–0.49] | [0.26–0.72] | [0.15–0.67] | [0.47–0.82] | [0.71–0.91] |
| DU-𝜌𝛼 | 𝜌 | 0.6 | 0.62 | 0.6 | 0.6 | 0.6 | 0.73 |
|  |  | [0.36–0.77] | [0.38–0.78] | [0.36–0.77] | [0.36–0.77] | [0.35–0.77] | [0.54–0.85] |
|  | 𝛼 | 0.58 | 0.69 | 0.58 | 0.58 | 0.58 | 0.66 |
|  |  | [0.33–0.76] | [0.49–0.83] | [0.33–0.76] | [0.33–0.76] | [0.32–0.75] | [0.44–0.81] |
| DU-2𝜌𝛼 | 𝜌_win_ | −0.01 | −0.01 | 0.45 | 0.45 | 0.63 | 0.84 |
|  |  | [−0.32–0.31] | [−0.32–0.3] | [0.16–0.67] | [0.16–0.67] | [0.39–0.79] | [0.72–0.91] |
|  | 𝜌_loss_ | 0.2 | 0.2 | 0.52 | 0.52 | 0.37 | 0.84 |
|  |  | [−0.12–0.49] | [−0.12–0.48] | [0.25–0.72] | [0.25–0.72] | [0.06–0.61] | [0.71–0.91] |
|  | 𝛼 | 0.17 | 0.18 | 0.21 | 0.21 | 0.59 | 0.83 |
|  |  | [−0.15–0.46] | [−0.14–0.47] | [−0.11–0.49] | [−0.11–0.49] | [0.34–0.76] | [0.69–0.91] |
| DU-𝜌2𝛼 | 𝜌 | −0.02 | −0.01 | 0.34 | 0.45 | 0.44 | 0.84 |
|  |  | [−0.33–0.3] | [−0.32–0.3] | [0.03–0.59] | [0.16–0.67] | [0.15–0.67] | [0.72–0.92] |
|  | 𝛼_win_ | 0.39 | −0.05 | 0.32 | 0.36 | 0.57 | −0.14 |
|  |  | [0.08–0.62] | [−0.36–0.27] | [0.01–0.58] | [0.05–0.6] | [0.32–0.75] | [−0.43–0.19] |
|  | 𝛼_loss_ | 0.19 | 0.26 | 0.21 | 0.29 | −0.18 | 0.37 |
|  |  | [−0.13–0.48] | [−0.06–0.53] | [−0.11–0.49] | [−0.02–0.55] | [−0.46–0.15] | [0.06–0.61] |
| DU-2𝜌2𝛼 | 𝜌_win_ | 0.15 | 0.16 | 0.48 | 0.48 | 0.5 | 0.91 |
|  |  | [−0.17–0.44] | [−0.16–0.45] | [0.19–0.69] | [0.19–0.69] | [0.22–0.7] | [0.83–0.95] |
|  | 𝜌_loss_ | 0.35 | 0.28 | 0.49 | 0.49 | 0.08 | 0.89 |
|  |  | [0.04–0.6] | [−0.03–0.55] | [0.21–0.7] | [0.21–0.7] | [−0.23–0.39] | [0.79–0.94] |
|  | 𝛼_win_ | −0.2 | 0.13 | 0.25 | −0.17 | −0.12 | −0.29 |
|  |  | [−0.48–0.12] | [−0.19–0.43] | [−0.07–0.52] | [−0.46–0.16] | [−0.42–0.2] | [−0.55–0.03] |
|  | 𝛼_loss_ | 0.1 | 0.04 | 0.12 | 0.37 | −0.36 | 0.63 |
|  |  | [−0.22–0.4] | [−0.28–0.35] | [−0.2–0.42] | [0.06–0.61] | [−0.6 to −0.05] | [0.39–0.79] |
| DU-𝜌𝛼𝜅 | 𝜌 | −0.03 | 0.17 | 0.52 | 0.52 | 0.56 | 0.71 |
|  |  | [−0.34–0.29] | [−0.15–0.46] | [0.25–0.72] | [0.25–0.72] | [0.3–0.74] | [0.51–0.84] |
|  | 𝛼 | 0.41 | 0.37 | 0.36 | 0.36 | 0.41 | 0.42 |
|  |  | [0.11–0.64] | [0.07–0.62] | [0.05–0.61] | [0.05–0.61] | [0.11–0.64] | [0.13–0.65] |
|  | 𝜅 | 0.41 | 0.26 | 0.29 | 0.29 | 0.45 | 0.78 |
|  |  | [0.11–0.64] | [−0.05–0.53] | [−0.03–0.55] | [−0.03–0.55] | [0.16–0.67] | [0.62–0.88] |
| DU-2𝜌𝛼𝜅 | 𝜌_win_ | −0.72 | 0.02 | 0.41 | 0.4 | 0.63 | 0.83 |
|  |  | [−0.84 to −0.52] | [−0.3–0.33] | [0.11–0.64] | [0.1–0.64] | [0.4–0.79] | [0.7–0.91] |
|  | 𝜌_loss_ | −0.7 | 0.01 | 0.41 | 0.49 | 0.46 | 0.94 |
|  |  | [−0.83 to −0.49] | [−0.3–0.33] | [0.11–0.64] | [0.2–0.69] | [0.17–0.68] | [0.89–0.97] |
|  | 𝛼 | 0.28 | 0.25 | 0.33 | 0.33 | 0.25 | 0.59 |
|  |  | [−0.04–0.55] | [−0.07–0.52] | [0.02–0.58] | [0.02–0.59] | [−0.07–0.52] | [0.34–0.76] |
|  | 𝜅 | 0.06 | −0.01 | 0.14 | 0.01 | −0.19 | −0.58 |
|  |  | [−0.26–0.37] | [−0.32–0.31] | [−0.18–0.43] | [−0.3–0.33] | [−0.47–0.13] | [−0.76 to −0.32] |
| DU-𝜌2𝛼𝜅 | 𝜌 | −0.05 | 0.08 | 0.34 | 0.5 | 0.5 | 0.69 |
|  |  | [−0.36–0.27] | [−0.23–0.39] | [0.03–0.59] | [0.22–0.7] | [0.22–0.71] | [0.47–0.82] |
|  | 𝛼_win_ | 0.21 | −0.14 | 0.02 | 0.11 | −0.04 | 0.16 |
|  |  | [−0.11–0.5] | [−0.44–0.18] | [−0.3–0.33] | [−0.21–0.41] | [−0.35–0.28] | [−0.16–0.46] |
|  | 𝛼_loss_ | 0.39 | 0.12 | 0.25 | 0.25 | 0.59 | 0.73 |
|  |  | [0.09–0.63] | [−0.2–0.42] | [−0.07–0.53] | [−0.07–0.52] | [0.34–0.76] | [0.55–0.85] |
|  | 𝜅 | 0.42 | 0.21 | 0.19 | 0.54 | 0.45 | 0.72 |
|  |  | [0.13–0.65] | [−0.12–0.49] | [−0.13–0.48] | [0.28–0.73] | [0.16–0.67] | [0.53–0.84] |
| DU-2𝜌2𝛼𝜅 | 𝜌_win_ | 0.1 | 0.2 | 0.46 | 0.48 | 0.54 | 0.87 |
|  |  | [−0.22–0.4] | [−0.12–0.49] | [0.17–0.68] | [0.19–0.69] | [0.28–0.73] | [0.77–0.93] |
|  | 𝜌_loss_ | −0.1 | −0.04 | 0.3 | 0.3 | 0.04 | 0.91 |
|  |  | [−0.4–0.22] | [−0.35–0.28] | [−0.01–0.56] | [−0.02–0.56] | [−0.27–0.35] | [0.83–0.95] |
|  | 𝛼_win_ | 0.18 | 0.2 | 0.11 | 0.3 | 0.05 | −0.18 |
|  |  | [−0.14–0.47] | [−0.12–0.48] | [−0.21–0.41] | [−0.02–0.56] | [−0.27–0.36] | [−0.46–0.15] |
|  | 𝛼_loss_ | 0.25 | 0.23 | 0.26 | 0.31 | 0.43 | 0.95 |
|  |  | [−0.07–0.53] | [−0.09–0.51] | [−0.06–0.53] | [0–0.57] | [0.13–0.65] | [0.92–0.98] |
|  | 𝜅 | −0.22 | 0.23 | −0.02 | 0.01 | 0.05 | 0.28 |
|  |  | [−0.5–0.1] | [−0.09–0.51] | [−0.33–0.3] | [−0.31–0.32] | [−0.27–0.36] | [−0.04–0.55] |
| SU-𝛽𝛼 | 𝛽 | 0 | 0 | 0.24 | 0.24 | 0.41 | 0.57 |
|  |  | [−0.32–0.31] | [−0.31–0.31] | [−0.08–0.52] | [−0.08–0.52] | [0.11–0.64] | [0.31–0.75] |
|  | 𝛼 | 0.31 | 0.16 | 0.31 | 0.31 | 0.33 | 0.39 |
|  |  | [−0.01–0.57] | [−0.16–0.45] | [−0.01–0.57] | [−0.01–0.57] | [0.01–0.58] | [0.08–0.63] |
| SU-2𝛽𝛼 | 𝛽_win_ | −0.01 | −0.03 | −0.03 | −0.03 | 0.28 | 0.34 |
|  |  | [−0.33–0.3] | [−0.34–0.29] | [−0.34–0.29] | [−0.34–0.29] | [−0.03–0.55] | [0.03–0.59] |
|  | 𝛽_loss_ | 0 | −0.02 | 0.05 | 0.05 | 0.45 | 0.54 |
|  |  | [−0.31–0.31] | [−0.34–0.29] | [−0.27–0.36] | [−0.27–0.36] | [0.16–0.67] | [0.27–0.73] |
|  | 𝛼 | 0.14 | 0.12 | 0.17 | 0.17 | 0.28 | 0.34 |
|  |  | [−0.18–0.44] | [−0.2–0.42] | [−0.15–0.46] | [−0.15–0.46] | [−0.04–0.55] | [0.03–0.59] |
| SU-𝛽2𝛼 | 𝛽 | 0 | 0 | −0.07 | 0.11 | 0.48 | 0.74 |
|  |  | [−0.31–0.31] | [−0.31–0.31] | [−0.38–0.25] | [−0.21–0.41] | [0.2–0.69] | [0.56–0.86] |
|  | 𝛼_win_ | 0.04 | 0.04 | −0.08 | 0.03 | −0.09 | −0.04 |
|  |  | [−0.27–0.35] | [−0.27–0.35] | [−0.38–0.24] | [−0.29–0.34] | [−0.39–0.23] | [−0.35–0.28] |
|  | 𝛼_loss_ | 0.37 | 0.37 | 0.35 | 0.39 | 0.4 | 0.48 |
|  |  | [0.06–0.61] | [0.06–0.61] | [0.04–0.6] | [0.08–0.62] | [0.1–0.64] | [0.2–0.69] |
| SU-2𝛽2𝛼 | 𝛽_win_ | 0 | 0 | −0.04 | −0.05 | 0.4 | 0.43 |
|  |  | [−0.31–0.31] | [−0.31–0.31] | [−0.35–0.28] | [−0.36–0.27] | [0.1–0.64] | [0.13–0.65] |
|  | 𝛽_loss_ | −0.02 | −0.02 | −0.05 | 0.07 | 0.53 | 0.71 |
|  |  | [−0.33–0.3] | [−0.33–0.3] | [−0.36–0.27] | [−0.25–0.38] | [0.26–0.72] | [0.51–0.84] |
|  | 𝛼_win_ | 0.13 | −0.01 | 0.1 | 0.04 | 0.02 | −0.01 |
|  |  | [−0.19–0.43] | [−0.33–0.3] | [−0.22–0.4] | [−0.28–0.35] | [−0.29–0.34] | [−0.32–0.31] |
|  | 𝛼_loss_ | 0.14 | 0.15 | 0.23 | 0.18 | 0.41 | 0.48 |
|  |  | [−0.18–0.44] | [−0.17–0.44] | [−0.09–0.51] | [−0.15–0.46] | [0.11–0.64] | [0.2–0.69] |
| DU-𝛽𝛼 | 𝛽 | 0.5 | 0.5 | 0.51 | 0.51 | 0.53 | 0.69 |
|  |  | [0.23–0.71] | [0.23–0.71] | [0.23–0.71] | [0.23–0.71] | [0.26–0.72] | [0.48–0.83] |
|  | 𝛼 | 0.58 | 0.58 | 0.58 | 0.58 | 0.58 | 0.66 |
|  |  | [0.33–0.76] | [0.33–0.76] | [0.33–0.76] | [0.33–0.76] | [0.32–0.75] | [0.44–0.81] |
| DU-2𝛽𝛼 | 𝛽_win_ | −0.03 | 0 | 0.02 | 0.03 | 0.5 | 0.67 |
|  |  | [−0.34–0.29] | [−0.31–0.31] | [−0.29–0.34] | [−0.29–0.34] | [0.23–0.71] | [0.45–0.81] |
|  | 𝛽_loss_ | 0.56 | 0.54 | 0.29 | 0.47 | 0.53 | 0.65 |
|  |  | [0.29–0.74] | [0.28–0.73] | [−0.03–0.55] | [0.18–0.68] | [0.26–0.72] | [0.43–0.8] |
|  | 𝛼 | 0.46 | 0.36 | 0.04 | 0.17 | 0.54 | 0.64 |
|  |  | [0.17–0.67] | [0.06–0.61] | [−0.28–0.35] | [−0.15–0.46] | [0.27–0.73] | [0.41–0.8] |
| DU-𝛽2𝛼 | 𝛽 | 0 | −0.03 | 0.03 | 0.12 | 0.34 | 0.86 |
|  |  | [-0.31–0.31] | [−0.34–0.28] | [−0.28–0.34] | [−0.2–0.42] | [0.03–0.59] | [0.75–0.92] |
|  | 𝛼_win_ | 0.28 | 0.21 | 0.08 | 0.39 | 0.26 | −0.09 |
|  |  | [−0.04–0.54] | [−0.11–0.49] | [−0.24–0.39] | [0.08–0.62] | [−0.06–0.53] | [−0.39–0.23] |
|  | 𝛼_loss_ | 0.46 | 0.19 | 0.13 | 0.41 | 0.08 | 0.21 |
|  |  | [0.17–0.67] | [−0.13–0.47] | [−0.19–0.43] | [0.11–0.64] | [−0.24–0.39] | [−0.11–0.5] |
| DU-2𝛽2𝛼 | 𝛽_win_ | 0 | 0 | 0.08 | 0.1 | 0.28 | 0.87 |
|  |  | [−0.31–0.31] | [−0.32–0.31] | [−0.24–0.38] | [−0.22–0.4] | [−0.03–0.55] | [0.76–0.93] |
|  | 𝛽_loss_ | 0 | 0 | 0.09 | 0.1 | 0.4 | 0.75 |
|  |  | [−0.31–0.31] | [−0.31–0.31] | [−0.23–0.4] | [−0.22–0.4] | [0.1–0.63] | [0.57–0.86] |
|  | 𝛼_win_ | 0.43 | −0.07 | 0.48 | 0.14 | −0.07 | 0.47 |
|  |  | [0.13–0.66] | [−0.38–0.25] | [0.2–0.69] | [−0.18–0.43] | [−0.37–0.25] | [0.18–0.68] |
|  | 𝛼_loss_ | 0.33 | 0.41 | 0.24 | 0.14 | 0.61 | 0.74 |
|  |  | [0.02–0.58] | [0.11–0.64] | [−0.08–0.51] | [−0.18–0.44] | [0.36–0.77] | [0.55–0.85] |
| DU-𝛽𝛼𝜅 | 𝛽 | 0 | −0.01 | 0.35 | 0.4 | 0.48 | 0.78 |
|  |  | [−0.31–0.31] | [−0.32–0.31] | [0.04–0.6] | [0.1–0.64] | [0.19–0.69] | [0.61–0.88] |
|  | 𝛼 | 0.37 | 0.37 | 0.36 | 0.46 | 0.41 | 0.69 |
|  |  | [0.07–0.62] | [0.06–0.61] | [0.05–0.61] | [0.17–0.68] | [0.11–0.64] | [0.48–0.82] |
|  | 𝜅 | 0.26 | 0.21 | 0.29 | 0.29 | 0.45 | 0.67 |
|  |  | [−0.06–0.53] | [−0.11–0.49] | [−0.03–0.55] | [−0.03–0.55] | [0.16–0.67] | [0.45–0.81] |
| DU-2𝛽𝛼𝜅 | 𝛽_win_ | −0.02 | −0.02 | 0 | 0 | 0.45 | 0.56 |
|  |  | [−0.33–0.3] | [−0.33–0.3] | [−0.31–0.32] | [−0.31–0.32] | [0.16–0.67] | [0.3–0.74] |
|  | 𝛽_loss_ | 0 | 0 | 0.24 | 0.24 | 0.39 | 0.35 |
|  |  | [−0.31–0.31] | [−0.31–0.31] | [−0.08–0.52] | [−0.08–0.52] | [0.08–0.62] | [0.04–0.6] |
|  | 𝛼 | 0.2 | 0.14 | 0.2 | 0.2 | 0.33 | 0.24 |
|  |  | [−0.12–0.49] | [−0.18–0.43] | [−0.12–0.49] | [−0.12–0.49] | [0.02–0.58] | [−0.08–0.51] |
|  | 𝜅 | 0.27 | 0.13 | 0.18 | 0.18 | 0.35 | 0.36 |
|  |  | [−0.05–0.54] | [−0.19–0.43] | [−0.14–0.47] | [−0.14–0.47] | [0.04–0.6] | [0.05–0.61] |
| DU-𝛽2𝛼𝜅 | 𝛽 | 0 | −0.01 | 0.1 | 0.16 | 0.37 | 0.78 |
|  |  | [−0.31–0.31] | [−0.32–0.31] | [−0.22–0.4] | [−0.16–0.45] | [0.06–0.61] | [0.62–0.88] |
|  | 𝛼_win_ | 0.1 | −0.02 | 0.12 | 0.12 | −0.04 | 0.41 |
|  |  | [−0.22–0.4] | [−0.33–0.29] | [−0.2–0.42] | [−0.2–0.42] | [−0.35–0.28] | [0.11–0.64] |
|  | 𝛼_loss_ | 0.34 | −0.13 | 0.38 | 0.41 | 0.59 | 0.77 |
|  |  | [0.03–0.59] | [−0.43–0.19] | [0.07–0.62] | [0.11–0.64] | [0.34–0.76] | [0.61–0.87] |
|  | 𝜅 | 0.36 | 0.29 | 0.19 | 0.21 | 0.45 | 0.2 |
|  |  | [0.05–0.6] | [−0.03–0.55] | [−0.13–0.48] | [−0.11–0.5] | [0.16–0.67] | [−0.12–0.49] |
| DU-2𝛽2𝛼𝜅 | 𝛽_win_ | 0 | −0.01 | 0.16 | −0.02 | 0.39 | 0.74 |
|  |  | [−0.31–0.31] | [−0.32–0.31] | [−0.16–0.45] | [−0.33–0.3] | [0.08–0.63] | [0.56–0.86] |
|  | 𝛽_loss_ | −0.01 | 0 | 0.41 | 0.16 | 0.35 | 0.43 |
|  |  | [−0.32–0.31] | [−0.31–0.31] | [0.11–0.64] | [−0.17–0.45] | [0.05–0.6] | [0.13–0.66] |
|  | 𝛼_win_ | 0.09 | 0.28 | −0.12 | 0.05 | 0.02 | −0.5 |
|  |  | [−0.23–0.4] | [−0.04–0.55] | [−0.42–0.2] | [−0.27–0.36] | [−0.3–0.33] | [−0.7 to −0.22] |
|  | 𝛼_loss_ | −0.16 | −0.03 | 0.06 | 0.08 | 0.49 | 0.95 |
|  |  | [−0.45–0.16] | [−0.34–0.29] | [−0.26–0.37] | [−0.24–0.38] | [0.21–0.7] | [0.91–0.97] |
|  | 𝜅 | 0.21 | 0 | 0.21 | 0.22 | 0.53 | 0.42 |
|  |  | [−0.12–0.49] | [−0.31–0.32] | [−0.11–0.49] | [−0.1–0.5] | [0.26–0.73] | [0.12–0.65] |

| **Supplementary Table 3** Retest reliability (model-calculated *r*) of the parameters of all computational models | | |
| --- | --- | --- |
|  |  | **EM-MAP** |
|  |  | **Joint** |
| SU-𝜌𝛼 | 𝜌 | 0.58 |
|  | 𝛼 | 0.3 |
| SU-2𝜌𝛼 | 𝜌_win_ | 0.64 |
|  | 𝜌_loss_ | 0.6 |
|  | 𝛼 | 0.3 |
| SU-𝜌2𝛼 | 𝜌 | 0.72 |
|  | 𝛼_win_ | 0 |
|  | 𝛼_loss_ | 0.39 |
| SU-2𝜌2𝛼 | 𝜌_win_ | 0.43 |
|  | 𝜌_loss_ | 0.5 |
|  | 𝛼_win_ | −0.03 |
|  | 𝛼_loss_ | 0.76 |
| DU-𝜌𝛼 | 𝜌 | 0.72 |
|  | 𝛼 | 0.46 |
| DU-2𝜌𝛼 | 𝜌_win_ | 0.86 |
|  | 𝜌_loss_ | 0.86 |
|  | 𝛼 | 0.74 |
| DU-𝜌2𝛼 | 𝜌 | 0.87 |
|  | 𝛼_win_ | 0.1 |
|  | 𝛼_loss_ | 0.58 |
| DU-2𝜌2𝛼 | 𝜌_win_ | 0.86 |
|  | 𝜌_loss_ | 0.86 |
|  | 𝛼_win_ | 0.15 |
|  | 𝛼_loss_ | 0.9 |
| DU-𝜌𝛼𝜅 | 𝜌 | 0.7 |
|  | 𝛼 | 0.29 |
|  | 𝜅 | 0.74 |
| DU-2𝜌𝛼𝜅 | 𝜌_win_ | 0.86 |
|  | 𝜌_loss_ | 0.9 |
|  | 𝛼 | 0.67 |
|  | 𝜅 | −0.76 |
| DU-𝜌2𝛼𝜅 | 𝜌 | 0.66 |
|  | 𝛼_win_ | 0.19 |
|  | 𝛼_loss_ | 0.68 |
|  | 𝜅 | 0.64 |
| DU-2𝜌2𝛼𝜅 | 𝜌_win_ | 0.87 |
|  | 𝜌_loss_ | 0.86 |
|  | 𝛼_win_ | −0.05 |
|  | 𝛼_loss_ | 0.92 |
|  | 𝜅 | 0.38 |
| SU-𝛽𝛼 | 𝛽 | 0.58 |
|  | 𝛼 | 0.3 |
| SU-2𝛽𝛼 | 𝛽_win_ | 0.4 |
|  | 𝛽_loss_ | 0.48 |
|  | 𝛼 | 0.28 |
| SU-𝛽2𝛼 | 𝛽 | 0.72 |
|  | 𝛼_win_ | 0 |
|  | 𝛼_loss_ | 0.39 |
| SU-2𝛽2𝛼 | 𝛽_win_ | 0.45 |
|  | 𝛽_loss_ | 0.62 |
|  | 𝛼_win_ | 0.04 |
|  | 𝛼_loss_ | 0.4 |
| DU-𝛽𝛼 | 𝛽 | 0.72 |
|  | 𝛼 | 0.46 |
| DU-2𝛽𝛼 | 𝛽_win_ | 0.67 |
|  | 𝛽_loss_ | 0.59 |
|  | 𝛼 | 0.44 |
| DU-𝛽2𝛼 | 𝛽 | 0.87 |
|  | 𝛼_win_ | −0.21 |
|  | 𝛼_loss_ | 0.35 |
| DU-2𝛽2𝛼 | 𝛽_win_ | 0.83 |
|  | 𝛽_loss_ | 0.72 |
|  | 𝛼_win_ | 0.6 |
|  | 𝛼_loss_ | 0.71 |
| DU-𝛽𝛼𝜅 | 𝛽 | 0.77 |
|  | 𝛼 | 0.61 |
|  | 𝜅 | 0.61 |
| DU-2𝛽𝛼𝜅 | 𝛽_win_ | 0.56 |
|  | 𝛽_loss_ | 0.55 |
|  | 𝛼 | 0.24 |
|  | 𝜅 | 0.73 |
| DU-𝛽2𝛼𝜅 | 𝛽 | 0.85 |
|  | 𝛼_win_ | 0.55 |
|  | 𝛼_loss_ | 0.7 |
|  | 𝜅 | 0.3 |
| DU-2𝛽2𝛼𝜅 | 𝛽_win_ | 0.79 |
|  | 𝛽_loss_ | 0.69 |
|  | 𝛼_win_ | −0.34 |
|  | 𝛼_loss_ | 0.93 |
|  | 𝜅 | 0.58 |


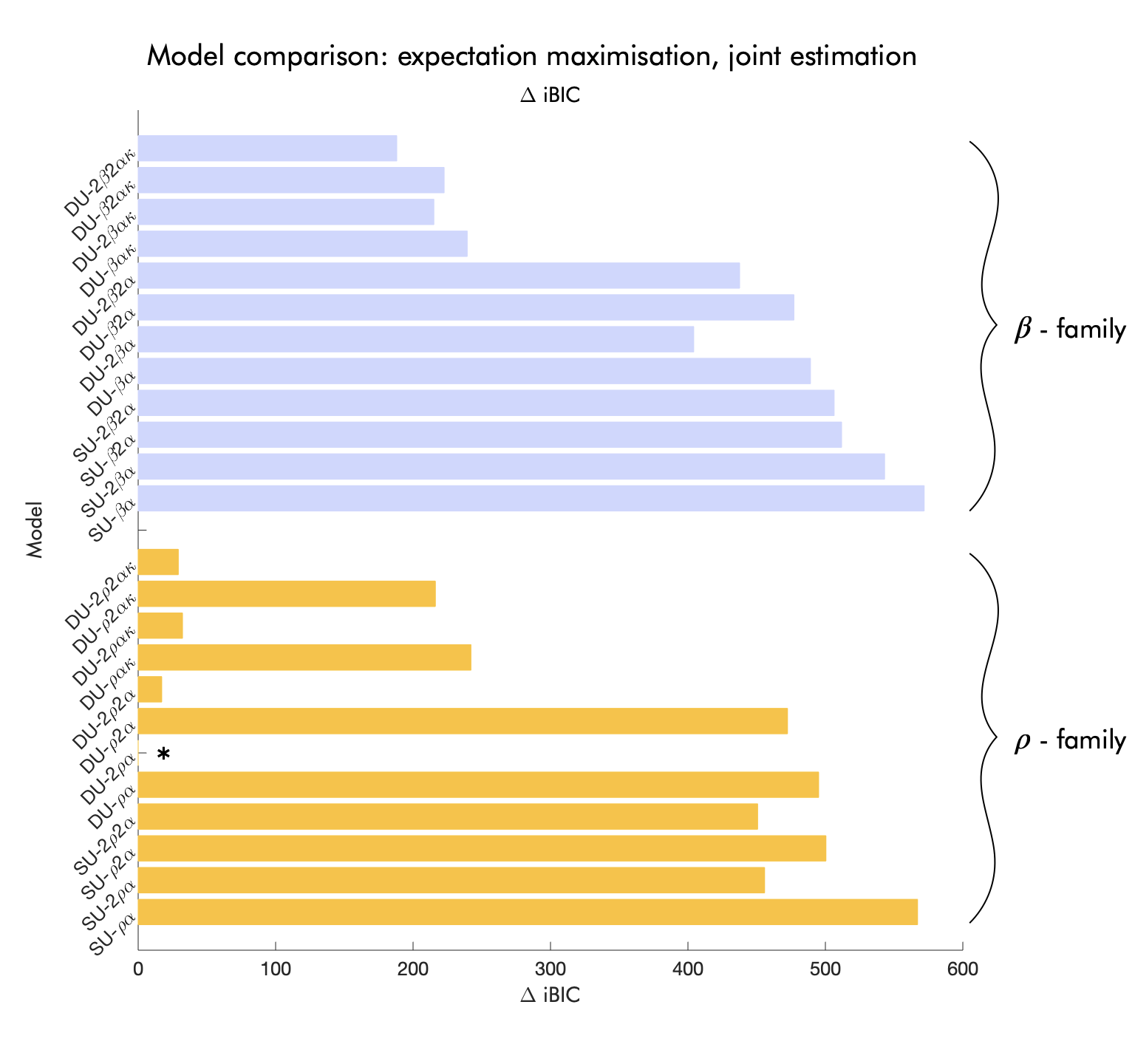


**Supplementary Fig. 1** Model comparison based on integrated BICs. Model comparison based on the integrated Bayesian information criterion (iBIC). Bars represent the distance from the model with the best evidence (lowest iBIC). Blue bars at the top of the plot represent models from the softmax (𝛽) family; yellow bars at the bottom of the plot represent models from the reinforcement sensitivity (𝜌) family. As represented by an asterisk, the overall winning model is a double update model with separate reinforcement sensitivities for wins and losses and a single learning rate (DU-2𝜌𝛼). Within the softmax family, the most complex model, a weighted double update with separate softmax temperatures and learning rates for wins and losses (DU-2𝛽2𝛼𝜅) had the best evidence. SU, single update; DU, double update; 𝛽 , softmax temperature; 𝛼, learning rate; 𝜅, double update weight; 𝜌, reinforcement sensitivity.

| **Supplementary Table 4** Retest reliability [ICC(A,1)] of the parameters of all computational models when estimated using a quasi-Newtonian optimization algorithm | | | | | | | |
| --- | --- | --- | --- | --- | --- | --- | --- |
|  |  | **ML** | | **MAP0** | | **EM-MAP** | |
|  |  | **Separate** | **Joint** | **Separate** | **Joint** | **Separate** | **Joint** |
| SU-𝜌𝛼 | 𝜌 | 0 | 0 | 0.26 | 0.3 | 0.42 | 0.75 |
|  |  | [−0.32–0.32] | [−0.32–0.32] | [−0.05–0.52] | [−0.01–0.56] | [0.14–0.65] | [0.55–0.86] |
|  | 𝛼 | 0.3 | 0.44 | 0.3 | 0.41 | 0.32 | 0.58 |
|  |  | [−0.02–0.57] | [0.14–0.67] | [−0.02–0.56] | [0.11–0.64] | [0–0.58] | [0.33–0.76] |
| SU-2𝜌𝛼 | 𝜌_win_ | 0.03 | 0 | 0.26 | 0.26 | 0.53 | 0.7 |
|  |  | [−0.28–0.33] | [−0.31–0.32] | [−0.03–0.52] | [−0.03–0.52] | [0.21–0.74] | [0.26–0.87] |
|  | 𝜌_loss_ | 0 | 0 | 0.34 | 0.34 | 0.38 | 0.88 |
|  |  | [−0.32–0.31] | [−0.32–0.31] | [0.02–0.59] | [0.02–0.59] | [0.08–0.62] | [0.76–0.94] |
|  | 𝛼 | 0.2 | 0.29 | 0.33 | 0.33 | 0.26 | 0.37 |
|  |  | [−0.13–0.48] | [−0.04–0.55] | [0.02–0.59] | [0.02–0.59] | [−0.06–0.53] | [0.06–0.62] |
| SU-𝜌2𝛼 | 𝜌 | 0.13 | −0.02 | 0.22 | 0.41 | 0.57 | 0.76 |
|  |  | [−0.19–0.43] | [−0.33–0.29] | [−0.1–0.51] | [0.12–0.64] | [0.31–0.75] | [0.59–0.87] |
|  | 𝛼_win_ | 0.02 | 0.04 | 0.06 | 0.02 | −0.05 | 0.03 |
|  |  | [−0.3–0.34] | [−0.28–0.35] | [−0.27–0.37] | [−0.3–0.34] | [−0.33–0.25] | [−0.24–0.31] |
|  | 𝛼_loss_ | 0.4 | 0.34 | 0.38 | 0.38 | 0.4 | 0.48 |
|  |  | [0.09–0.64] | [0.02–0.59] | [0.07–0.62] | [0.07–0.62] | [0.09–0.64] | [0.19–0.69] |
| SU-2𝜌2𝛼 | 𝜌_win_ | 0 | −0.03 | 0.12 | 0.12 | 0.45 | 0.58 |
|  |  | [−0.3–0.31] | [−0.35–0.3] | [−0.17–0.41] | [−0.17–0.41] | [0.17–0.67] | [0.33–0.76] |
|  | 𝜌_loss_ | −0.02 | −0.06 | 0.36 | 0.36 | 0.25 | 0.66 |
|  |  | [−0.34–0.3] | [−0.37–0.27] | [0.04–0.61] | [0.04–0.61] | [−0.07–0.53] | [0.44–0.81] |
|  | 𝛼_win_ | 0.01 | −0.07 | 0.05 | 0.05 | 0.03 | −0.04 |
|  |  | [−0.32–0.33] | [−0.39–0.26] | [−0.27–0.36] | [−0.27–0.36] | [−0.29–0.34] | [−0.34–0.28] |
|  | 𝛼_loss_ | 0.25 | 0.22 | 0.44 | 0.44 | 0.54 | 0.84 |
|  |  | [−0.08–0.52] | [−0.11–0.5] | [0.14–0.66] | [0.14–0.66] | [0.28–0.73] | [0.7–0.91] |
| DU-𝜌𝛼 | 𝜌 | 0.61 | 0.61 | 0.61 | 0.61 | 0.61 | 0.74 |
|  |  | [0.36–0.78] | [0.36–0.78] | [0.36–0.78] | [0.36–0.78] | [0.34–0.78] | [0.48–0.87] |
|  | 𝛼 | 0.58 | 0.58 | 0.58 | 0.58 | 0.58 | 0.66 |
|  |  | [0.32–0.76] | [0.32–0.76] | [0.32–0.76] | [0.32–0.76] | [0.32–0.75] | [0.44–0.81] |
| DU-2𝜌𝛼 | 𝜌_win_ | 0 | 0 | 0.46 | 0.46 | 0.64 | 0.85 |
|  |  | [−0.32–0.32] | [−0.32–0.31] | [0.18–0.68] | [0.18–0.68] | [0.31–0.82] | [0.26–0.95] |
|  | 𝜌_loss_ | 0.22 | 0.2 | 0.52 | 0.52 | 0.42 | 0.84 |
|  |  | [−0.11–0.5] | [−0.13–0.49] | [0.25–0.72] | [0.25–0.72] | [0.08–0.66] | [0.25–0.95] |
|  | 𝛼 | 0.16 | 0.16 | 0.2 | 0.2 | 0.59 | 0.83 |
|  |  | [−0.17–0.46] | [−0.18–0.46] | [−0.13–0.49] | [−0.13–0.49] | [0.33–0.76] | [0.69–0.91] |
| DU-𝜌2𝛼 | 𝜌 | −0.08 | −0.03 | 0.34 | 0.34 | 0.44 | 0.95 |
|  |  | [−0.4–0.25] | [−0.36–0.29] | [0.03–0.59] | [0.03–0.59] | [0.14–0.67] | [0.91–0.97] |
|  | 𝛼_win_ | 0.27 | 0.4 | 0.44 | 0.45 | −0.09 | 0.67 |
|  |  | [−0.06–0.54] | [0.09–0.63] | [0.14–0.67] | [0.15–0.67] | [−0.31–0.17] | [0.45–0.81] |
|  | 𝛼_loss_ | 0.39 | 0.21 | 0.28 | 0.31 | 0.28 | 0.82 |
|  |  | [0.08–0.63] | [−0.11–0.5] | [−0.04–0.55] | [−0.01–0.57] | [−0.01–0.54] | [0.67–0.9] |
| DU-2𝜌2𝛼 | 𝜌_win_ | 0.04 | 0.45 | 0.48 | 0.48 | 0.51 | 0.87 |
|  |  | [−0.28–0.35] | [0.15–0.67] | [0.2–0.69] | [0.2–0.69] | [0.22–0.71] | [0.69–0.94] |
|  | 𝜌_loss_ | 0.66 | 0.48 | 0.49 | 0.49 | 0.06 | 0.91 |
|  |  | [0.43–0.81] | [0.2–0.69] | [0.2–0.7] | [0.2–0.7] | [−0.27–0.37] | [0.81–0.95] |
|  | 𝛼_win_ | 0.29 | −0.11 | 0.14 | 0.11 | 0.19 | −0.28 |
|  |  | [−0.04–0.55] | [−0.4–0.2] | [−0.19–0.44] | [−0.23–0.41] | [−0.08–0.45] | [−0.56–0.04] |
|  | 𝛼_loss_ | −0.07 | −0.13 | 0.23 | 0.38 | 0.08 | −0.4 |
|  |  | [−0.38–0.25] | [−0.43–0.19] | [−0.1–0.51] | [0.07–0.62] | [−0.16–0.35] | [−0.65 to −0.08] |
| DU-𝜌𝛼𝜅 | 𝜌 | −0.03 | −0.07 | 0.53 | 0.58 | 0.57 | 0.8 |
|  |  | [−0.34–0.29] | [−0.36–0.24] | [0.26–0.72] | [0.33–0.76] | [0.3–0.75] | [0.59–0.9] |
|  | 𝛼 | 0.31 | 0.24 | 0.36 | 0.46 | 0.41 | 0.7 |
|  |  | [0–0.57] | [−0.09–0.52] | [0.04–0.61] | [0.17–0.68] | [0.11–0.64] | [0.42–0.84] |
|  | 𝜅 | 0.13 | 0.23 | 0.28 | 0.29 | 0.45 | 0.68 |
|  |  | [−0.19–0.42] | [−0.08–0.51] | [−0.04–0.55] | [−0.03–0.55] | [0.16–0.67] | [0.42–0.83] |
| DU-2𝜌𝛼𝜅 | 𝜌_win_ | 0.06 | 0.08 | 0.42 | 0.44 | 0.62 | 0.86 |
|  |  | [−0.26–0.37] | [−0.25–0.39] | [0.13–0.65] | [0.16–0.66] | [0.32–0.8] | [0.16–0.96] |
|  | 𝜌_loss_ | 0.71 | 0.22 | 0.29 | 0.56 | 0.47 | 0.94 |
|  |  | [0.51–0.84] | [−0.11–0.5] | [−0.03–0.55] | [0.29–0.74] | [0.19–0.68] | [0.49–0.98] |
|  | 𝛼 | 0.12 | 0.32 | 0.32 | 0.34 | 0.29 | 0.58 |
|  |  | [−0.21–0.42] | [−0.01–0.58] | [0–0.58] | [0.02–0.59] | [0–0.55] | [0.22–0.78] |
|  | 𝜅 | 0.17 | 0.07 | 0.07 | 0.31 | −0.17 | −0.5 |
|  |  | [−0.15–0.46] | [−0.26–0.38] | [−0.25–0.38] | [−0.01–0.57] | [−0.45–0.15] | [−0.73 to −0.18] |
| DU-𝜌2𝛼𝜅 | 𝜌 | −0.1 | 0 | 0.39 | 0.45 | 0.5 | 0.83 |
|  |  | [−0.41–0.23] | [−0.33–0.32] | [0.09–0.63] | [0.16–0.67] | [0.23–0.71] | [0.71–0.91] |
|  | 𝛼_win_ | −0.09 | −0.02 | −0.02 | 0.07 | −0.03 | 0.17 |
|  |  | [−0.4–0.24] | [−0.34–0.3] | [−0.34–0.3] | [−0.25–0.38] | [−0.34–0.28] | [−0.13–0.45] |
|  | 𝛼_loss_ | 0.45 | 0.31 | 0.41 | 0.41 | 0.59 | 0.91 |
|  |  | [0.15–0.67] | [−0.01–0.57] | [0.1–0.64] | [0.1–0.64] | [0.34–0.76] | [0.83–0.95] |
|  | 𝜅 | 0.08 | 0.42 | 0.29 | 0.32 | 0.45 | 0.67 |
|  |  | [−0.24–0.38] | [0.13–0.65] | [−0.03–0.55] | [0.02–0.57] | [0.15–0.67] | [0.45–0.81] |
| DU-2𝜌2𝛼𝜅 | 𝜌_win_ | 0 | 0 | 0.48 | 0.48 | 0.55 | 0.82 |
|  |  | [−0.32–0.32] | [−0.32–0.32] | [0.2–0.69] | [0.2–0.69] | [0.28–0.73] | [0.56–0.92] |
|  | 𝜌_loss_ | −0.03 | −0.07 | 0.23 | 0.28 | 0.03 | 0.87 |
|  |  | [−0.31–0.27] | [−0.37–0.24] | [−0.09–0.51] | [−0.05–0.55] | [−0.29–0.35] | [0.74–0.94] |
|  | 𝛼_win_ | −0.07 | 0.04 | −0.02 | 0.05 | 0.04 | 0 |
|  |  | [−0.38–0.25] | [−0.27–0.34] | [−0.34–0.31] | [−0.28–0.37] | [−0.29–0.36] | [−0.33–0.32] |
|  | 𝛼_loss_ | 0.26 | 0.34 | 0.3 | 0.37 | 0.43 | 0.97 |
|  |  | [−0.07–0.54] | [0.02–0.59] | [−0.02–0.56] | [0.06–0.61] | [0.13–0.65] | [0.94–0.98] |
|  | 𝜅 | 0.1 | 0.16 | −0.04 | −0.01 | 0.05 | −0.45 |
|  |  | [−0.23–0.41] | [−0.15–0.44] | [−0.34–0.28] | [−0.32–0.31] | [−0.28–0.36] | [−0.69 to −0.14] |
| SU-𝛽𝛼 | 𝛽 | 0 | 0 | 0.26 | 0.3 | 0.42 | 0.75 |
|  |  | [−0.32–0.32] | [−0.32–0.32] | [−0.05–0.52] | [−0.01–0.56] | [0.14–0.65] | [0.55–0.86] |
|  | 𝛼 | 0.3 | 0.3 | 0.3 | 0.41 | 0.32 | 0.58 |
|  |  | [−0.02–0.57] | [−0.02–0.57] | [−0.02–0.56] | [0.11–0.64] | [0–0.58] | [0.33–0.76] |
| SU-2𝛽𝛼 | 𝛽_win_ | 0 | 0 | −0.03 | −0.01 | 0.29 | 0.52 |
|  |  | [−0.29–0.3] | [−0.32–0.32] | [−0.35–0.29] | [−0.33–0.31] | [−0.02–0.55] | [0.24–0.72] |
|  | 𝛽_loss_ | 0 | −0.03 | 0.04 | 0.06 | 0.47 | 0.65 |
|  |  | [−0.32–0.32] | [−0.36–0.29] | [−0.28–0.35] | [−0.26–0.37] | [0.18–0.68] | [0.41–0.8] |
|  | 𝛼 | 0.05 | 0.05 | 0.16 | 0.27 | 0.28 | 0.52 |
|  |  | [−0.28–0.36] | [−0.28–0.36] | [−0.17–0.46] | [−0.05–0.54] | [−0.04–0.55] | [0.25–0.71] |
| SU-𝛽2𝛼 | 𝛽 | 0 | 0 | −0.02 | 0.29 | 0.49 | 0.74 |
|  |  | [−0.32–0.32] | [−0.32–0.32] | [−0.34–0.3] | [−0.02–0.55] | [0.21–0.7] | [0.56–0.86] |
|  | 𝛼_win_ | 0.01 | 0.02 | 0.06 | 0.12 | −0.05 | 0.03 |
|  |  | [−0.31–0.33] | [−0.31–0.33] | [−0.27–0.37] | [−0.2–0.42] | [−0.33–0.25] | [−0.24–0.31] |
|  | 𝛼_loss_ | 0.37 | 0.42 | 0.38 | 0.37 | 0.4 | 0.48 |
|  |  | [0.06–0.62] | [0.12–0.65] | [0.07–0.62] | [0.05–0.61] | [0.09–0.64] | [0.19–0.69] |
| SU-2𝛽2𝛼 | 𝛽_win_ | −0.02 | 0 | 0 | −0.05 | 0.42 | 0.43 |
|  |  | [−0.34–0.3] | [−0.32–0.32] | [−0.31–0.32] | [−0.37–0.27] | [0.13–0.64] | [0.14–0.66] |
|  | 𝛽_loss_ | −0.04 | 0 | 0.06 | 0.06 | 0.54 | 0.71 |
|  |  | [−0.35–0.28] | [−0.32–0.32] | [−0.27–0.37] | [−0.27–0.37] | [0.27–0.73] | [0.51–0.84] |
|  | 𝛼_win_ | 0.11 | 0.16 | 0.1 | 0.03 | 0.06 | 0.01 |
|  |  | [−0.21–0.41] | [−0.16–0.46] | [−0.23–0.4] | [−0.29–0.35] | [−0.23–0.35] | [−0.29–0.32] |
|  | 𝛼_loss_ | 0.02 | 0.19 | 0.21 | 0.17 | 0.4 | 0.48 |
|  |  | [−0.3–0.34] | [−0.15–0.48] | [−0.11–0.5] | [−0.17–0.46] | [0.09–0.64] | [0.19–0.69] |
| DU-𝛽𝛼 | 𝛽 | 0.52 | 0.52 | 0.52 | 0.52 | 0.54 | 0.7 |
|  |  | [0.24–0.71] | [0.24–0.71] | [0.25–0.72] | [0.25–0.72] | [0.26–0.74] | [0.41–0.85] |
|  | 𝛼 | 0.58 | 0.58 | 0.58 | 0.58 | 0.58 | 0.66 |
|  |  | [0.32–0.76] | [0.32–0.76] | [0.32–0.76] | [0.32–0.76] | [0.32–0.75] | [0.44–0.81] |
| DU-2𝛽𝛼 | 𝛽_win_ | 0 | 0 | 0.03 | 0.03 | 0.52 | 0.68 |
|  |  | [NaN–NaN] | [−0.32–0.32] | [−0.3–0.35] | [−0.3–0.34] | [0.24–0.72] | [0.42–0.83] |
|  | 𝛽_loss_ | 0.4 | 0.15 | 0.5 | 0.45 | 0.54 | 0.66 |
|  |  | [0.09–0.64] | [−0.18–0.45] | [0.22–0.7] | [0.16–0.67] | [0.27–0.73] | [0.41–0.81] |
|  | 𝛼 | 0.24 | −0.07 | 0.19 | 0.13 | 0.54 | 0.64 |
|  |  | [−0.08–0.52] | [−0.38–0.26] | [−0.14–0.48] | [−0.21–0.43] | [0.27–0.73] | [0.41–0.8] |
| DU-𝛽2𝛼 | 𝛽 | −0.01 | 0 | 0.04 | 0.04 | 0.31 | 0.79 |
|  |  | [−0.33–0.31] | [−0.32–0.32] | [−0.27–0.35] | [−0.27–0.35] | [−0.01–0.58] | [0.59–0.89] |
|  | 𝛼_win_ | −0.03 | 0.4 | 0.35 | 0.13 | −0.09 | −0.46 |
|  |  | [−0.35–0.29] | [0.1–0.63] | [0.03–0.6] | [−0.2–0.44] | [−0.31–0.17] | [−0.65–0.03] |
|  | 𝛼_loss_ | 0.16 | 0.02 | 0.08 | 0.58 | 0.28 | 0.95 |
|  |  | [−0.16–0.45] | [−0.29–0.33] | [−0.25–0.39] | [0.33–0.76] | [−0.01–0.54] | [0.81–0.98] |
| DU-2𝛽2𝛼 | 𝛽_win_ | 0 | 0 | 0.05 | 0.09 | 0.28 | 0.92 |
|  |  | [−0.32–0.32] | [−0.32–0.32] | [−0.27–0.36] | [−0.23–0.39] | [−0.04–0.55] | [0.85–0.96] |
|  | 𝛽_loss_ | 0 | 0 | 0.06 | 0.08 | 0.4 | 0.74 |
|  |  | [−0.32–0.32] | [−0.32–0.31] | [−0.27–0.38] | [−0.25–0.39] | [0.1–0.63] | [0.55–0.86] |
|  | 𝛼_win_ | 0 | −0.17 | −0.07 | −0.09 | 0.6 | 0.41 |
|  |  | [−0.32–0.33] | [−0.48–0.16] | [−0.39–0.26] | [−0.38–0.23] | [0.35–0.77] | [0.12–0.64] |
|  | 𝛼_loss_ | 0.02 | 0.19 | −0.17 | 0.11 | −0.04 | 0.72 |
|  |  | [−0.29–0.33] | [−0.14–0.48] | [−0.47–0.16] | [−0.23–0.41] | [−0.33–0.27] | [0.53–0.85] |
| DU-𝛽𝛼𝜅 | 𝛽 | 0 | 0 | 0.36 | 0.41 | 0.49 | 0.78 |
|  |  | [−0.32–0.32] | [−0.32–0.32] | [0.07–0.61] | [0.12–0.64] | [0.21–0.7] | [0.55–0.89] |
|  | 𝛼 | 0.44 | 0.36 | 0.36 | 0.46 | 0.41 | 0.7 |
|  |  | [0.15–0.66] | [0.05–0.61] | [0.04–0.61] | [0.17–0.68] | [0.11–0.64] | [0.42–0.84] |
|  | 𝜅 | 0.34 | 0.16 | 0.28 | 0.29 | 0.45 | 0.68 |
|  |  | [0.03–0.59] | [−0.16–0.45] | [−0.04–0.55] | [−0.03–0.55] | [0.16–0.67] | [0.42–0.83] |
| DU-2𝛽𝛼𝜅 | 𝛽_win_ | 0 | −0.01 | 0 | 0.02 | 0.46 | 0.76 |
|  |  | [−0.32–0.31] | [−0.33–0.31] | [−0.32–0.32] | [−0.3–0.34] | [0.17–0.67] | [0.58–0.87] |
|  | 𝛽_loss_ | 0 | 0 | 0.26 | 0.28 | 0.42 | 0.44 |
|  |  | [−0.32–0.32] | [−0.32–0.32] | [−0.04–0.53] | [−0.03–0.54] | [0.12–0.65] | [0.15–0.66] |
|  | 𝛼 | 0.04 | 0.07 | 0.17 | 0.42 | 0.35 | 0.49 |
|  |  | [−0.28–0.36] | [−0.26–0.38] | [−0.16–0.47] | [0.13–0.65] | [0.05–0.59] | [0.11–0.72] |
|  | 𝜅 | 0.37 | 0.15 | 0.12 | 0.33 | 0.38 | 0.46 |
|  |  | [0.08–0.61] | [−0.18–0.45] | [−0.19–0.41] | [0.02–0.59] | [0.08–0.62] | [0.09–0.7] |
| DU-𝛽2𝛼𝜅 | 𝛽 | 0 | 0 | 0.14 | 0.11 | 0.36 | 0.71 |
|  |  | [−0.32–0.32] | [−0.32–0.32] | [−0.2–0.44] | [−0.22–0.41] | [0.05–0.61] | [0.5–0.84] |
|  | 𝛼_win_ | −0.1 | 0.01 | −0.1 | −0.16 | −0.03 | 0.17 |
|  |  | [−0.41–0.23] | [−0.32–0.33] | [−0.41–0.23] | [−0.47–0.17] | [−0.34–0.28] | [−0.13–0.45] |
|  | 𝛼_loss_ | 0.34 | 0.42 | 0.38 | 0.43 | 0.59 | 0.91 |
|  |  | [0.02–0.6] | [0.12–0.65] | [0.07–0.63] | [0.13–0.66] | [0.34–0.76] | [0.83–0.95] |
|  | 𝜅 | 0.33 | 0.4 | 0.4 | 0.4 | 0.45 | 0.67 |
|  |  | [0.02–0.58] | [0.1–0.64] | [0.1–0.63] | [0.1–0.63] | [0.15–0.67] | [0.45–0.81] |
| DU-2𝛽2𝛼𝜅 | 𝛽_win_ | 0 | −0.01 | 0.11 | −0.11 | 0.32 | 0.68 |
|  |  | [−0.32–0.32] | [−0.32–0.31] | [−0.2–0.41] | [−0.42–0.22] | [0–0.58] | [0.47–0.82] |
|  | 𝛽_loss_ | 0 | 0 | 0.15 | 0.26 | 0.32 | 0.47 |
|  |  | [−0.31–0.32] | [−0.32–0.32] | [−0.18–0.45] | [−0.04–0.52] | [0.03–0.57] | [0.18–0.68] |
|  | 𝛼_win_ | 0.32 | 0.1 | 0.15 | −0.01 | 0.03 | −0.05 |
|  |  | [0.01–0.57] | [−0.24–0.4] | [−0.18–0.44] | [−0.33–0.32] | [−0.28–0.34] | [−0.32–0.25] |
|  | 𝛼_loss_ | −0.18 | −0.18 | 0.29 | 0.09 | 0.13 | 0.8 |
|  |  | [−0.49–0.15] | [−0.48–0.15] | [−0.02–0.55] | [−0.25–0.4] | [−0.1–0.37] | [0.65–0.89] |
|  | 𝜅 | −0.15 | −0.24 | 0.31 | 0.14 | 0.37 | 0.54 |
|  |  | [−0.42–0.15] | [−0.5–0.07] | [−0.01–0.57] | [−0.16–0.42] | [0.04–0.63] | [0.18–0.75] |

| **Supplementary Table 5** Retest reliability [ICC(1)] of the parameters of all computational models when estimated using a quasi-Newtonian optimization algorithm | | | | | | | |
| --- | --- | --- | --- | --- | --- | --- | --- |
|  |  | **ML** | | **MAP0** | | **EM-MAP** | |
|  |  | **Separate** | **Joint** | **Separate** | **Joint** | **Separate** | **Joint** |
| SU-𝜌𝛼 | 𝜌 | 0 | 0 | 0.26 | 0.3 | 0.42 | 0.75 |
|  |  | [−0.32–0.32] | [−0.32–0.32] | [−0.05–0.52] | [−0.01–0.56] | [0.14–0.65] | [0.55–0.86] |
|  | 𝛼 | 0.3 | 0.44 | 0.3 | 0.41 | 0.32 | 0.58 |
|  |  | [−0.02–0.57] | [0.14–0.67] | [−0.02–0.56] | [0.11–0.64] | [0–0.58] | [0.33–0.76] |
| SU-2𝜌𝛼 | 𝜌_win_ | 0.03 | 0 | 0.26 | 0.26 | 0.53 | 0.7 |
|  |  | [−0.28–0.33] | [−0.31–0.32] | [−0.03–0.52] | [−0.03–0.52] | [0.21–0.74] | [0.26–0.87] |
|  | 𝜌_loss_ | 0 | 0 | 0.34 | 0.34 | 0.38 | 0.88 |
|  |  | [−0.32–0.31] | [−0.32–0.31] | [0.02–0.59] | [0.02–0.59] | [0.08–0.62] | [0.76–0.94] |
|  | 𝛼 | 0.2 | 0.29 | 0.33 | 0.33 | 0.26 | 0.37 |
|  |  | [−0.13–0.48] | [−0.04–0.55] | [0.02–0.59] | [0.02–0.59] | [−0.06–0.53] | [0.06–0.62] |
| SU-𝜌2𝛼 | 𝜌 | 0.13 | −0.02 | 0.22 | 0.41 | 0.57 | 0.76 |
|  |  | [−0.19–0.43] | [−0.33–0.29] | [−0.1–0.51] | [0.12–0.64] | [0.31–0.75] | [0.59–0.87] |
|  | 𝛼_win_ | 0.02 | 0.04 | 0.06 | 0.02 | −0.05 | 0.03 |
|  |  | [−0.3–0.34] | [−0.28–0.35] | [−0.27–0.37] | [−0.3–0.34] | [−0.33–0.25] | [−0.24–0.31] |
|  | 𝛼_loss_ | 0.4 | 0.34 | 0.38 | 0.38 | 0.4 | 0.48 |
|  |  | [0.09–0.64] | [0.02–0.59] | [0.07–0.62] | [0.07–0.62] | [0.09–0.64] | [0.19–0.69] |
| SU-2𝜌2𝛼 | 𝜌_win_ | 0 | −0.03 | 0.12 | 0.12 | 0.45 | 0.58 |
|  |  | [−0.3–0.31] | [−0.35–0.3] | [−0.17–0.41] | [−0.17–0.41] | [0.17–0.67] | [0.33–0.76] |
|  | 𝜌_loss_ | −0.02 | −0.06 | 0.36 | 0.36 | 0.25 | 0.66 |
|  |  | [−0.34–0.3] | [−0.37–0.27] | [0.04–0.61] | [0.04–0.61] | [−0.07–0.53] | [0.44–0.81] |
|  | 𝛼_win_ | 0.01 | −0.07 | 0.05 | 0.05 | 0.03 | −0.04 |
|  |  | [−0.32–0.33] | [−0.39–0.26] | [−0.27–0.36] | [−0.27–0.36] | [−0.29–0.34] | [−0.34–0.28] |
|  | 𝛼_loss_ | 0.25 | 0.22 | 0.44 | 0.44 | 0.54 | 0.84 |
|  |  | [−0.08–0.52] | [−0.11–0.5] | [0.14–0.66] | [0.14–0.66] | [0.28–0.73] | [0.7–0.91] |
| DU-𝜌𝛼 | 𝜌 | 0.61 | 0.61 | 0.61 | 0.61 | 0.61 | 0.74 |
|  |  | [0.36–0.78] | [0.36–0.78] | [0.36–0.78] | [0.36–0.78] | [0.34–0.78] | [0.48–0.87] |
|  | 𝛼 | 0.58 | 0.58 | 0.58 | 0.58 | 0.58 | 0.66 |
|  |  | [0.32–0.76] | [0.32–0.76] | [0.32–0.76] | [0.32–0.76] | [0.32–0.75] | [0.44–0.81] |
| DU-2𝜌𝛼 | 𝜌_win_ | 0 | 0 | 0.46 | 0.46 | 0.64 | 0.85 |
|  |  | [−0.32–0.32] | [−0.32–0.31] | [0.18–0.68] | [0.18–0.68] | [0.31–0.82] | [0.26–0.95] |
|  | 𝜌_loss_ | 0.22 | 0.2 | 0.52 | 0.52 | 0.42 | 0.84 |
|  |  | [−0.11–0.5] | [−0.13–0.49] | [0.25–0.72] | [0.25–0.72] | [0.08–0.66] | [0.25–0.95] |
|  | 𝛼 | 0.16 | 0.16 | 0.2 | 0.2 | 0.59 | 0.83 |
|  |  | [−0.17–0.46] | [−0.18–0.46] | [−0.13–0.49] | [−0.13–0.49] | [0.33–0.76] | [0.69–0.91] |
| DU-𝜌2𝛼 | 𝜌 | −0.08 | −0.03 | 0.34 | 0.34 | 0.44 | 0.95 |
|  |  | [−0.4–0.25] | [−0.36–0.29] | [0.03–0.59] | [0.03–0.59] | [0.14–0.67] | [0.91–0.97] |
|  | 𝛼_win_ | 0.27 | 0.4 | 0.44 | 0.45 | −0.09 | 0.67 |
|  |  | [−0.06–0.54] | [0.09–0.63] | [0.14–0.67] | [0.15–0.67] | [−0.31–0.17] | [0.45–0.81] |
|  | 𝛼_loss_ | 0.39 | 0.21 | 0.28 | 0.31 | 0.28 | 0.82 |
|  |  | [0.08–0.63] | [−0.11–0.5] | [−0.04–0.55] | [−0.01–0.57] | [−0.01–0.54] | [0.67–0.9] |
| DU-2𝜌2𝛼 | 𝜌_win_ | 0.04 | 0.45 | 0.48 | 0.48 | 0.51 | 0.87 |
|  |  | [−0.28–0.35] | [0.15–0.67] | [0.2–0.69] | [0.2–0.69] | [0.22–0.71] | [0.69–0.94] |
|  | 𝜌_loss_ | 0.66 | 0.48 | 0.49 | 0.49 | 0.06 | 0.91 |
|  |  | [0.43–0.81] | [0.2–0.69] | [0.2–0.7] | [0.2–0.7] | [−0.27–0.37] | [0.81–0.95] |
|  | 𝛼_win_ | 0.29 | −0.11 | 0.14 | 0.11 | 0.19 | −0.28 |
|  |  | [−0.04–0.55] | [−0.4–0.2] | [−0.19–0.44] | [−0.23–0.41] | [−0.08–0.45] | [−0.56–0.04] |
|  | 𝛼_loss_ | −0.07 | −0.13 | 0.23 | 0.38 | 0.08 | −0.4 |
|  |  | [−0.38–0.25] | [−0.43–0.19] | [−0.1–0.51] | [0.07–0.62] | [−0.16–0.35] | [−0.65 to −0.08] |
| DU-𝜌𝛼𝜅 | 𝜌 | −0.03 | −0.07 | 0.53 | 0.58 | 0.57 | 0.8 |
|  |  | [−0.34–0.29] | [−0.36–0.24] | [0.26–0.72] | [0.33–0.76] | [0.3–0.75] | [0.59–0.9] |
|  | 𝛼 | 0.31 | 0.24 | 0.36 | 0.46 | 0.41 | 0.7 |
|  |  | [0–0.57] | [−0.09–0.52] | [0.04–0.61] | [0.17–0.68] | [0.11–0.64] | [0.42–0.84] |
|  | 𝜅 | 0.13 | 0.23 | 0.28 | 0.29 | 0.45 | 0.68 |
|  |  | [−0.19–0.42] | [−0.08–0.51] | [−0.04–0.55] | [−0.03–0.55] | [0.16–0.67] | [0.42–0.83] |
| DU-2𝜌𝛼𝜅 | 𝜌_win_ | 0.06 | 0.08 | 0.42 | 0.44 | 0.62 | 0.86 |
|  |  | [−0.26–0.37] | [−0.25–0.39] | [0.13–0.65] | [0.16–0.66] | [0.32–0.8] | [0.16–0.96] |
|  | 𝜌_loss_ | 0.71 | 0.22 | 0.29 | 0.56 | 0.47 | 0.94 |
|  |  | [0.51–0.84] | [−0.11–0.5] | [−0.03–0.55] | [0.29–0.74] | [0.19–0.68] | [0.49–0.98] |
|  | 𝛼 | 0.12 | 0.32 | 0.32 | 0.34 | 0.29 | 0.58 |
|  |  | [−0.21–0.42] | [−0.01–0.58] | [0–0.58] | [0.02–0.59] | [0–0.55] | [0.22–0.78] |
|  | 𝜅 | 0.17 | 0.07 | 0.07 | 0.31 | −0.17 | −0.5 |
|  |  | [−0.15–0.46] | [−0.26–0.38] | [−0.25–0.38] | [−0.01–0.57] | [−0.45–0.15] | [−0.73 to −0.18] |
| DU-𝜌2𝛼𝜅 | 𝜌 | −0.1 | 0 | 0.39 | 0.45 | 0.5 | 0.83 |
|  |  | [−0.41–0.23] | [−0.33–0.32] | [0.09–0.63] | [0.16–0.67] | [0.23–0.71] | [0.71–0.91] |
|  | 𝛼_win_ | −0.09 | −0.02 | −0.02 | 0.07 | −0.03 | 0.17 |
|  |  | [−0.4–0.24] | [−0.34–0.3] | [−0.34–0.3] | [−0.25–0.38] | [−0.34–0.28] | [−0.13–0.45] |
|  | 𝛼_loss_ | 0.45 | 0.31 | 0.41 | 0.41 | 0.59 | 0.91 |
|  |  | [0.15–0.67] | [−0.01–0.57] | [0.1–0.64] | [0.1–0.64] | [0.34–0.76] | [0.83–0.95] |
|  | 𝜅 | 0.08 | 0.42 | 0.29 | 0.32 | 0.45 | 0.67 |
|  |  | [−0.24–0.38] | [0.13–0.65] | [−0.03–0.55] | [0.02–0.57] | [0.15–0.67] | [0.45–0.81] |
| DU-2𝜌2𝛼𝜅 | 𝜌_win_ | 0 | 0 | 0.48 | 0.48 | 0.55 | 0.82 |
|  |  | [−0.32–0.32] | [−0.32–0.32] | [0.2–0.69] | [0.2–0.69] | [0.28–0.73] | [0.56–0.92] |
|  | 𝜌_loss_ | −0.03 | −0.07 | 0.23 | 0.28 | 0.03 | 0.87 |
|  |  | [−0.31–0.27] | [−0.37–0.24] | [−0.09–0.51] | [−0.05–0.55] | [−0.29–0.35] | [0.74–0.94] |
|  | 𝛼_win_ | −0.07 | 0.04 | −0.02 | 0.05 | 0.04 | 0 |
|  |  | [−0.38–0.25] | [−0.27–0.34] | [−0.34–0.31] | [−0.28–0.37] | [−0.29–0.36] | [−0.33–0.32] |
|  | 𝛼_loss_ | 0.26 | 0.34 | 0.3 | 0.37 | 0.43 | 0.97 |
|  |  | [−0.07–0.54] | [0.02–0.59] | [−0.02–0.56] | [0.06–0.61] | [0.13–0.65] | [0.94–0.98] |
|  | 𝜅 | 0.1 | 0.16 | −0.04 | −0.01 | 0.05 | −0.45 |
|  |  | [−0.23–0.41] | [−0.15–0.44] | [−0.34–0.28] | [−0.32–0.31] | [−0.28–0.36] | [−0.69 to −0.14] |
| SU-𝛽𝛼 | 𝛽 | 0 | 0 | 0.26 | 0.3 | 0.42 | 0.75 |
|  |  | [−0.32–0.32] | [−0.32–0.32] | [−0.05–0.52] | [−0.01–0.56] | [0.14–0.65] | [0.55–0.86] |
|  | 𝛼 | 0.3 | 0.3 | 0.3 | 0.41 | 0.32 | 0.58 |
|  |  | [−0.02–0.57] | [−0.02–0.57] | [−0.02–0.56] | [0.11–0.64] | [0–0.58] | [0.33–0.76] |
| SU-2𝛽𝛼 | 𝛽_win_ | 0 | 0 | −0.03 | −0.01 | 0.29 | 0.52 |
|  |  | [−0.31–0.31] | [−0.32–0.32] | [−0.35–0.29] | [−0.33–0.31] | [−0.02–0.55] | [0.24–0.72] |
|  | 𝛽_loss_ | 0 | −0.03 | 0.04 | 0.06 | 0.47 | 0.65 |
|  |  | [−0.32–0.32] | [−0.36–0.29] | [−0.28–0.35] | [−0.26–0.37] | [0.18–0.68] | [0.41–0.8] |
|  | 𝛼 | 0.17 | 0.05 | 0.16 | 0.27 | 0.28 | 0.52 |
|  |  | [−0.17–0.46] | [−0.28–0.36] | [−0.17–0.46] | [−0.05–0.54] | [−0.04–0.55] | [0.25–0.71] |
| SU-𝛽2𝛼 | 𝛽 | 0 | 0 | 0.13 | 0.29 | 0.49 | 0.74 |
|  |  | [−0.32–0.32] | [−0.32–0.32] | [−0.17–0.42] | [−0.02–0.55] | [0.21–0.7] | [0.56–0.86] |
|  | 𝛼_win_ | 0.02 | 0.02 | 0.06 | 0.12 | −0.05 | 0.03 |
|  |  | [−0.31–0.33] | [−0.31–0.33] | [−0.27–0.37] | [−0.2–0.42] | [−0.33–0.25] | [−0.24–0.31] |
|  | 𝛼_loss_ | 0.37 | 0.42 | 0.38 | 0.37 | 0.4 | 0.48 |
|  |  | [0.06–0.62] | [0.12–0.65] | [0.07–0.62] | [0.05–0.61] | [0.09–0.64] | [0.19–0.69] |
| SU-2𝛽2𝛼 | 𝛽_win_ | 0 | 0 | −0.05 | −0.05 | 0.42 | 0.43 |
|  |  | [−0.32–0.32] | [−0.32–0.32] | [−0.35–0.27] | [−0.37–0.27] | [0.13–0.64] | [0.14–0.66] |
|  | 𝛽_loss_ | −0.01 | 0 | 0.06 | 0.06 | 0.54 | 0.71 |
|  |  | [−0.31–0.31] | [−0.32–0.32] | [−0.27–0.37] | [−0.27–0.37] | [0.27–0.73] | [0.51–0.84] |
|  | 𝛼_win_ | 0.08 | 0.16 | 0.11 | 0.03 | 0.06 | 0.01 |
|  |  | [−0.25–0.39] | [−0.16–0.46] | [−0.21–0.41] | [−0.29–0.35] | [−0.23–0.35] | [−0.29–0.32] |
|  | 𝛼_loss_ | 0.06 | 0.19 | 0.22 | 0.17 | 0.4 | 0.48 |
|  |  | [−0.26–0.37] | [−0.15–0.48] | [−0.11–0.5] | [−0.17–0.46] | [0.09–0.64] | [0.19–0.69] |
| DU-𝛽𝛼 | 𝛽 | 0.52 | 0.52 | 0.52 | 0.52 | 0.54 | 0.7 |
|  |  | [0.24–0.71] | [0.24–0.71] | [0.25–0.72] | [0.25–0.72] | [0.26–0.74] | [0.41–0.85] |
|  | 𝛼 | 0.58 | 0.58 | 0.58 | 0.58 | 0.58 | 0.66 |
|  |  | [0.32–0.76] | [0.32–0.76] | [0.32–0.76] | [0.32–0.76] | [0.32–0.75] | [0.44–0.81] |
| DU-2𝛽𝛼 | 𝛽_win_ | 0 | 0 | 0.02 | 0.03 | 0.52 | 0.68 |
|  |  | [NaN–NaN] | [−0.32–0.32] | [−0.31–0.34] | [−0.3–0.34] | [0.24–0.72] | [0.42–0.83] |
|  | 𝛽_loss_ | 0.25 | 0.15 | 0.47 | 0.45 | 0.54 | 0.66 |
|  |  | [−0.08–0.53] | [−0.18–0.45] | [0.19–0.68] | [0.16–0.67] | [0.27–0.73] | [0.41–0.81] |
|  | 𝛼 | 0.17 | −0.07 | 0.24 | 0.13 | 0.54 | 0.64 |
|  |  | [−0.16–0.46] | [−0.38–0.26] | [−0.09–0.52] | [−0.21–0.43] | [0.27–0.73] | [0.41–0.8] |
| DU-𝛽2𝛼 | 𝛽 | 0 | 0 | 0.04 | 0.04 | 0.31 | 0.79 |
|  |  | [−0.32–0.32] | [−0.32–0.32] | [−0.27–0.35] | [−0.27–0.35] | [−0.01–0.58] | [0.59–0.89] |
|  | 𝛼_win_ | −0.01 | 0.4 | 0.17 | 0.13 | 0.57 | −0.46 |
|  |  | [−0.33–0.31] | [0.1–0.63] | [−0.11–0.45] | [−0.2–0.44] | [0.31–0.75] | [−0.65–0.03] |
|  | 𝛼_loss_ | 0.03 | 0.02 | 0.42 | 0.58 | −0.19 | 0.95 |
|  |  | [−0.3–0.35] | [−0.29–0.33] | [0.14–0.65] | [0.33–0.76] | [−0.49–0.14] | [0.81–0.98] |
| DU-2𝛽2𝛼 | 𝛽_win_ | 0 | 0 | 0.06 | 0.09 | 0.28 | 0.92 |
|  |  | [−0.32–0.32] | [−0.32–0.32] | [−0.25–0.37] | [−0.23–0.39] | [−0.04–0.55] | [0.85–0.96] |
|  | 𝛽_loss_ | 0 | 0 | 0.08 | 0.08 | 0.4 | 0.74 |
|  |  | [−0.32–0.32] | [−0.32–0.31] | [−0.25–0.39] | [−0.25–0.39] | [0.1–0.63] | [0.55–0.86] |
|  | 𝛼_win_ | 0.37 | −0.17 | 0.25 | −0.09 | 0.02 | 0.41 |
|  |  | [0.05–0.61] | [−0.48–0.16] | [−0.08–0.53] | [−0.38–0.23] | [−0.2–0.28] | [0.12–0.64] |
|  | 𝛼_loss_ | 0.33 | 0.19 | 0.17 | 0.11 | 0.25 | 0.72 |
|  |  | [0.02–0.58] | [−0.14–0.48] | [−0.16–0.46] | [−0.23–0.41] | [−0.04–0.51] | [0.53–0.85] |
| DU-𝛽𝛼𝜅 | 𝛽 | 0 | 0 | 0.36 | 0.41 | 0.49 | 0.78 |
|  |  | [−0.32–0.32] | [−0.32–0.32] | [0.07–0.61] | [0.12–0.64] | [0.21–0.7] | [0.55–0.89] |
|  | 𝛼 | 0.34 | 0.36 | 0.36 | 0.46 | 0.41 | 0.7 |
|  |  | [0.03–0.59] | [0.05–0.61] | [0.04–0.61] | [0.17–0.68] | [0.11–0.64] | [0.42–0.84] |
|  | 𝜅 | 0.29 | 0.16 | 0.28 | 0.29 | 0.45 | 0.68 |
|  |  | [−0.02–0.56] | [−0.16–0.45] | [−0.04–0.55] | [−0.03–0.55] | [0.16–0.67] | [0.42–0.83] |
| DU-2𝛽𝛼𝜅 | 𝛽_win_ | 0 | −0.01 | 0 | 0.02 | 0.46 | 0.76 |
|  |  | [−0.32–0.32] | [−0.33–0.31] | [−0.32–0.32] | [−0.3–0.34] | [0.17–0.67] | [0.58–0.87] |
|  | 𝛽_loss_ | 0 | 0 | 0.26 | 0.28 | 0.42 | 0.44 |
|  |  | [−0.32–0.31] | [−0.32–0.32] | [−0.04–0.53] | [−0.03–0.54] | [0.12–0.65] | [0.15–0.66] |
|  | 𝛼 | 0.11 | 0.07 | 0.17 | 0.42 | 0.35 | 0.49 |
|  |  | [−0.21–0.41] | [−0.26–0.38] | [−0.16–0.47] | [0.13–0.65] | [0.05–0.59] | [0.11–0.72] |
|  | 𝜅 | 0.23 | 0.15 | 0.12 | 0.33 | 0.38 | 0.46 |
|  |  | [−0.07–0.5] | [−0.18–0.45] | [−0.19–0.41] | [0.02–0.59] | [0.08–0.62] | [0.09–0.7] |
| DU-𝛽2𝛼𝜅 | 𝛽 | 0 | 0 | 0.26 | 0.11 | 0.27 | 0.71 |
|  |  | [−0.32–0.32] | [−0.32–0.32] | [−0.06–0.54] | [−0.22–0.41] | [−0.06–0.54] | [0.5–0.84] |
|  | 𝛼_win_ | −0.15 | 0.01 | 0 | −0.16 | −0.02 | 0.17 |
|  |  | [−0.45–0.17] | [−0.32–0.33] | [−0.32–0.32] | [−0.47–0.17] | [−0.34–0.3] | [−0.13–0.45] |
|  | 𝛼_loss_ | 0.37 | 0.42 | 0.24 | 0.43 | 0.64 | 0.91 |
|  |  | [0.06–0.61] | [0.12–0.65] | [−0.08–0.52] | [0.13–0.66] | [0.41–0.8] | [0.83–0.95] |
|  | 𝜅 | 0.31 | 0.4 | 0.39 | 0.4 | 0.43 | 0.67 |
|  |  | [0–0.56] | [0.1–0.64] | [0.1–0.63] | [0.1–0.63] | [0.12–0.66] | [0.45–0.81] |
| DU-2𝛽2𝛼𝜅 | 𝛽_win_ | 0 | −0.01 | 0.18 | −0.11 | 0.26 | 0.68 |
|  |  | [−0.32–0.32] | [−0.32–0.31] | [−0.14–0.46] | [−0.42–0.22] | [−0.06–0.53] | [0.47–0.82] |
|  | 𝛽_loss_ | 0 | 0 | 0.43 | 0.26 | 0.35 | 0.47 |
|  |  | [−0.32–0.31] | [−0.32–0.32] | [0.13–0.65] | [−0.04–0.52] | [0.05–0.6] | [0.18–0.68] |
|  | 𝛼_win_ | 0.25 | 0.1 | 0.16 | −0.01 | 0.01 | −0.05 |
|  |  | [−0.07–0.53] | [−0.24–0.4] | [−0.16–0.46] | [−0.33–0.32] | [−0.32–0.33] | [−0.32–0.25] |
|  | 𝛼_loss_ | −0.11 | −0.18 | 0.32 | 0.09 | 0.5 | 0.8 |
|  |  | [−0.42–0.22] | [−0.48–0.15] | [0.01–0.57] | [−0.25–0.4] | [0.22–0.7] | [0.65–0.89] |
|  | 𝜅 | −0.02 | −0.24 | 0.38 | 0.14 | 0.44 | 0.54 |
|  |  | [−0.29–0.27] | [−0.5–0.07] | [0.07–0.62] | [−0.16–0.42] | [0.15–0.66] | [0.18–0.75] |

| **Supplementary Table 6** Retest reliability (model-calculated *r*) of the parameters of all computational models when estimated using a quasi-Newtonian optimization algorithm | | |
| --- | --- | --- |
|  |  | **EM-MAP** |
|  |  | **Joint** |
| SU-𝜌𝛼 | 𝜌 | 0.69 |
|  | 𝛼 | 0.51 |
| SU-2𝜌𝛼 | 𝜌_win_ | 0.64 |
|  | 𝜌_loss_ | 0.6 |
|  | 𝛼 | 0.3 |
| SU-𝜌2𝛼 | 𝜌 | 0.72 |
|  | 𝛼_win_ | 0 |
|  | 𝛼_loss_ | 0.39 |
| SU-2𝜌2𝛼 | 𝜌_win_ | 0.43 |
|  | 𝜌_loss_ | 0.5 |
|  | 𝛼_win_ | −0.03 |
|  | 𝛼_loss_ | 0.76 |
| DU-𝜌𝛼 | 𝜌 | 0.72 |
|  | 𝛼 | 0.46 |
| DU-2𝜌𝛼 | 𝜌_win_ | 0.86 |
|  | 𝜌_loss_ | 0.86 |
|  | 𝛼 | 0.74 |
| DU-𝜌2𝛼 | 𝜌 | 0.91 |
|  | 𝛼_win_ | 0.71 |
|  | 𝛼_loss_ | 0.82 |
| DU-2𝜌2𝛼 | 𝜌_win_ | 0.85 |
|  | 𝜌_loss_ | 0.87 |
|  | 𝛼_win_ | −0.43 |
|  | 𝛼_loss_ | −0.39 |
| DU-𝜌𝛼𝜅 | 𝜌 | 0.77 |
|  | 𝛼 | 0.61 |
|  | 𝜅 | 0.61 |
| DU-2𝜌𝛼𝜅 | 𝜌_win_ | 0.89 |
|  | 𝜌_loss_ | 0.92 |
|  | 𝛼 | 0.68 |
|  | 𝜅 | −0.74 |
| DU-𝜌2𝛼𝜅 | 𝜌 | 0.8 |
|  | 𝛼_win_ | 0.21 |
|  | 𝛼_loss_ | 0.82 |
|  | 𝜅 | 0.57 |
| DU-2𝜌2𝛼𝜅 | 𝜌_win_ | 0.81 |
|  | 𝜌_loss_ | 0.89 |
|  | 𝛼_win_ | −0.01 |
|  | 𝛼_loss_ | 0.95 |
|  | 𝜅 | −0.46 |
| SU-𝛽𝛼 | 𝛽 | 0.69 |
|  | 𝛼 | 0.51 |
| SU-2𝛽𝛼 | 𝛽_win_ | 0.5 |
|  | 𝛽_loss_ | 0.57 |
|  | 𝛼 | 0.47 |
| SU-𝛽2𝛼 | 𝛽 | 0.72 |
|  | 𝛼_win_ | 0 |
|  | 𝛼_loss_ | 0.39 |
| SU-2𝛽2𝛼 | 𝛽_win_ | 0.45 |
|  | 𝛽_loss_ | 0.62 |
|  | 𝛼_win_ | 0.04 |
|  | 𝛼_loss_ | 0.4 |
| DU-𝛽𝛼 | 𝛽 | 0.72 |
|  | 𝛼 | 0.46 |
| DU-2𝛽𝛼 | 𝛽_win_ | 0.67 |
|  | 𝛽_loss_ | 0.59 |
|  | 𝛼 | 0.44 |
| DU-𝛽2𝛼 | 𝛽 | 0.87 |
|  | 𝛼_win_ | −0.6 |
|  | 𝛼_loss_ | 0.97 |
| DU-2𝛽2𝛼 | 𝛽_win_ | 0.86 |
|  | 𝛽_loss_ | 0.69 |
|  | 𝛼_win_ | 0.48 |
|  | 𝛼_loss_ | 0.7 |
| DU-𝛽𝛼𝜅 | 𝛽 | 0.77 |
|  | 𝛼 | 0.61 |
|  | 𝜅 | 0.61 |
| DU-2𝛽𝛼𝜅 | 𝛽_win_ | 0.68 |
|  | 𝛽_loss_ | 0.61 |
|  | 𝛼 | 0.45 |
|  | 𝜅 | 0.56 |
| DU-𝛽2𝛼𝜅 | 𝛽 | 0.8 |
|  | 𝛼_win_ | 0.21 |
|  | 𝛼_loss_ | 0.82 |
|  | 𝜅 | 0.57 |
| DU-2𝛽2𝛼𝜅 | 𝛽_win_ | 0.66 |
|  | 𝛽_loss_ | 0.67 |
|  | 𝛼_win_ | 0.09 |
|  | 𝛼_loss_ | 0.76 |
|  | 𝜅 | 0.7 |

**
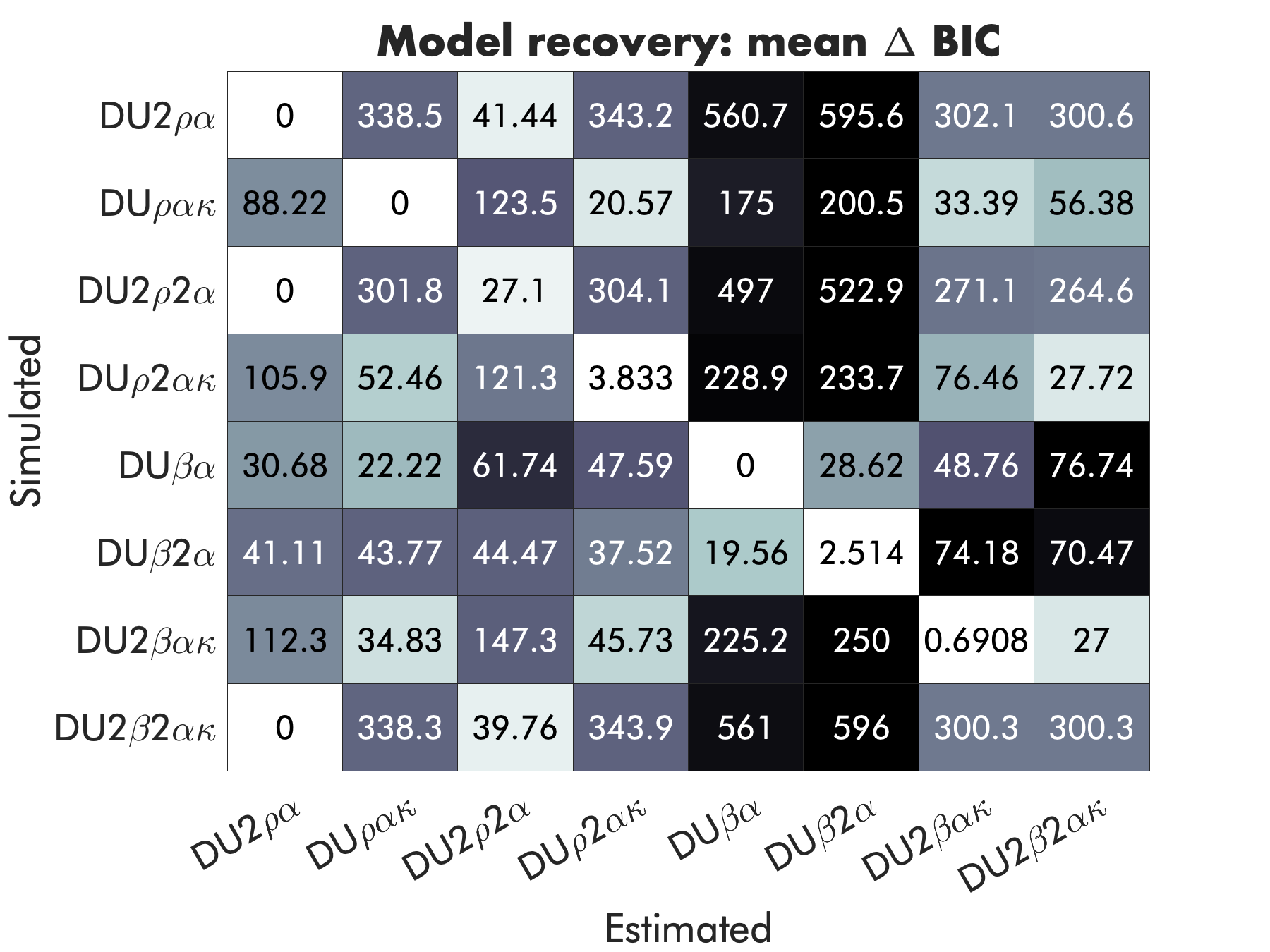
**

**Supplementary Fig. 2** Model recoverability. The confusion matrix shows, for each model, the average deviation of each fitted model’s integrated BIC from the integrated BIC of the model with the best evidence. Lighter colors indicate smaller deviation. Perfect recoverability would be indicated by zeros along the diagonal.

| **Supplementary Table 7** Average correlation between true and recovered parameters based on 10 simulated samples per model | | | |
| --- | --- | --- | --- |
|  |  | **Session 1** | **Session 2** |
| DU-2𝜌𝛼 | 𝜌_win_ | 0.94 | 0.91 |
|  | 𝜌_loss_ | 0.81 | 0.81 |
|  | 𝛼 | 0.93 | 0.91 |
| DU-𝜌𝛼𝜅 | 𝜌 | 0.84 | 0.89 |
|  | 𝛼 | 0.65 | 0.7 |
|  | 𝜅 | 0.73 | 0.78 |
| DU-2𝜌2𝛼 | 𝜌_win_ | 0.38 | 0.5 |
|  | 𝜌_loss_ | 0.27 | 0.02 |
|  | 𝛼_win_ | 0.35 | 0.46 |
|  | 𝛼_loss_ | 0.46 | 0.73 |
| DU-𝜌2𝛼𝜅 | 𝜌 | 0.44 | 0.59 |
|  | 𝛼_win_ | 0.74 | 0.83 |
|  | 𝛼_loss_ | 0.7 | 0.55 |
|  | 𝜅 | 0.91 | 0.92 |
| DU-𝛽𝛼 | 𝛽 | 0.94 | 0.95 |
|  | 𝛼 | 0.81 | 0.63 |
| DU-𝛽2𝛼 | 𝛽 | 0.37 | 0.5 |
|  | 𝛼_win_ | 0.04 | 0.47 |
|  | 𝛼_loss_ | 0.91 | 0.9 |
| DU-2𝛽𝛼𝜅 | 𝛽_win_ | 0.89 | 0.93 |
|  | 𝛽_loss_ | 0.75 | 0.85 |
|  | 𝛼 | 0.85 | 0.73 |
|  | 𝜅 | 0.57 | 0.32 |
| DU-2𝛽2𝛼𝜅 | 𝛽_win_ | 0.59 | 0.59 |
|  | 𝛽_loss_ | −0.33 | 0.31 |
|  | 𝛼_win_ | 0.34 | 0.52 |
|  | 𝛼_loss_ | 0.48 | 0.43 |
|  | 𝜅 | 0.94 | 0.91 |

| **Supplementary Table 8** Mean ICC(1) between parameters for sessions 1 and 2, recovered using ML, MAP0, and EM-MAP estimation, based on 100 simulated datasets | | | | |
| --- | --- | --- | --- | --- |
|  |  | **ML** | **MAP0** | **EM-MAP** |
| DU-2𝜌𝛼 | 𝜌_win_ | 0.26 [−0.03–0.52] | 0.63 [0.4–0.79] | 0.86 [0.76–0.93] |
|  | 𝜌_loss_ | 0.17 [−0.14–0.46] | 0.29 [−0.01–0.55] | 0.75 [0.58–0.86] |
|  | 𝛼 | 0.19 [−0.12–0.47] | 0.24 [−0.07–0.51] | 0.7 [0.51–0.83] |
| DU-2𝛽2𝛼𝜅 | 𝛽_win_ | 0.01 [−0.3–0.32] | 0.27 [−0.04–0.53] | 0.73 [0.56–0.85] |
|  | 𝛽_loss_ | 0.04 [−0.27–0.34] | 0.33 [0.03–0.58] | 0.65 [0.43–0.8] |
|  | 𝛼_win_ | −0.04 [−0.35–0.27] | −0.11 [−0.4–0.2] | −0.31 [−0.54 to −0.03] |
|  | 𝛼_loss_ | 0.29 [−0.02–0.55] | 0.48 [0.2–0.69] | 0.86 [0.77–0.92] |
|  | 𝜅 | 0.1 [−0.21–0.4] | 0.19 [−0.13–0.47] | 0.33 [0.04–0.57] |

| **Supplementary Table 9** Mean model-calculated Pearson correlations between parameters for sessions 1 and 2, recovered using EM-MAP estimation, based on 100 simulated datasets | | |
| --- | --- | --- |
|  |  | **EM-MAP** |
| DU-2𝜌𝛼 | 𝜌_win_ | 0.92 |
|  | 𝜌_loss_ | 0.76 |
|  | 𝛼 | 0.63 |
| DU-2𝛽2𝛼𝜅 | 𝛽_win_ | 0.76 |
|  | 𝛽_loss_ | 0.69 |
|  | 𝛼_win_ | −0.17 |
|  | 𝛼_loss_ | 0.86 |
|  | 𝜅 | 0.43 |

| **Supplementary Table 10** Mean ICC(A,1) between raw behavioral performance indices for sessions 1 and 2 based on 100 datasets simulated using the fit parameters of the DU-2𝜌𝛼 model and the DU-2𝛽2𝛼𝜅 model, respectively | | |
| --- | --- | --- |
|  | DU-2𝜌𝛼 | DU-2𝛽2𝛼𝜅 |
| Accuracy | 0.64 (0.42–0.79) | 0.75 (0.58–0.86) |
| Perseveration | 0.9 (0.78–0.93) | 0.81 (0.65–0.87) |
| Switching overall | 0.86 (0.71–0.93) | 0.72 (0.47–0.85) |
| Switching after losses | 0.97 (0.94–0.98) | 0.9 (0.81–0.95) |
| Switching after wins | 0.79 (0.61–0.89) | 0.63 (0.36–0.79) |

| **Supplementary Table 11** Mean ICC(1) between raw behavioral performance indices for sessions 1 and 2 based on 100 datasets simulated using the fit parameters of the DU-2𝜌𝛼 model and the DU-2𝛽2𝛼𝜅 model, respectively | | |
| --- | --- | --- |
|  | DU-2𝜌𝛼 | DU-2𝛽2𝛼𝜅 |
| Accuracy | 0.64 (0.42–0.79) | 0.75 (0.58–0.86) |
| Perseveration | 0.9 (0.79–0.93) | 0.81 (0.65–0.87) |
| Switching overall | 0.85 (0.74–0.92) | 0.71 (0.52–0.84) |
| Switching after losses | 0.97 (0.93–0.98) | 0.9 (0.81–0.94) |
| Switching after wins | 0.78 (0.63–0.88) | 0.62 (0.38–0.78) |

| **Supplementary Table 12** Mean Pearson correlation between raw behavioral performance indices for sessions 1 and 2 based on 100 datasets simulated using the fit parameters of the DU-2𝜌𝛼 model and the DU-2𝛽2𝛼𝜅 model, respectively. | | |
| --- | --- | --- |
|  | DU-2𝜌𝛼 | DU-2𝛽2𝛼𝜅 |
| Accuracy | 0.64 (0.42–0.79) | 0.75 (0.58–0.86) |
| Perseveration | 0.9 (0.83–0.95) | 0.81 (0.7–0.89) |
| Switching overall | 0.89 (0.81–0.94) | 0.79 (0.63–0.88) |
| Switching after losses | 0.97 (0.95–0.99) | 0.91 (0.83–0.95) |
| Switching after wins | 0.85 (0.73–0.92) | 0.73 (0.54–0.85) |
